# scCDC: a computational method for gene-specific contamination detection and correction in single-cell and single-nucleus RNA-seq data

**DOI:** 10.1186/s13059-024-03284-w

**Published:** 2024-05-23

**Authors:** Weijian Wang, Yihui Cen, Zezhen Lu, Yueqing Xu, Tianyi Sun, Ying Xiao, Wanlu Liu, Jingyi Jessica Li, Chaochen Wang

**Affiliations:** 1 Centre of Biomedical Systems and Informatics, International Campus, ZJU-UoE Institute, Zhejiang University School of Medicine, Zhejiang University, Haining, Zhejiang 314400 China; 2 Department of Statistics and Data Science, University of California, Los Angeles, CA 90095 USA; 3 Sir Run Run Shaw Hospital, School of Medicine, Zhejiang University, Hangzhou, Zhejiang 310020 China; 4 Department of Gynecology, The Second Affiliated Hospital, Zhejiang University School of Medicine, Zhejiang University, Hangzhou, Zhejiang 310020 China; 5 Biomedical and Health Translational Research Centre, Zhejiang University, Haining, Zhejiang 314400 China

## Abstract

In droplet-based single-cell and single-nucleus RNA-seq assays, systematic contamination of ambient RNA molecules biases the quantification of gene expression levels. Existing methods correct the contamination for all genes globally. However, there lacks specific evaluation of correction efficacy for varying contamination levels. Here, we show that DecontX and CellBender under-correct highly contaminating genes, while SoupX and scAR over-correct lowly/non-contaminating genes. Here, we develop scCDC as the first method to detect the contamination-causing genes and only correct expression levels of these genes, some of which are cell-type markers. Compared with existing decontamination methods, scCDC excels in decontaminating highly contaminating genes while avoiding over-correction of other genes.

**Supplementary Information:**

The online version contains supplementary material available at 10.1186/s13059-024-03284-w.

## Background

Single-cell RNA-seq (scRNA-seq) is a widely used technique for studying cell heterogeneity in organs. Various studies and large databases, such as the human cell atlas, have taken advantage of scRNA-seq, especially droplet-based platforms such as Chromium X, BD Rhapsody, and inDrop [1–3]. Droplet-based scRNA-seq requires every cell to be sealed with a barcoded bead in a droplet so that the cell’s mRNAs can be labeled by the specific barcode. However, ambient RNA contamination is ubiquitous [4–7]: ambient RNA molecules in the solution would cause systematic contamination by inflating the measured expression levels of endogenous genes in cells, thus impeding the identification of cell-type marker genes. In parallel to scRNA-seq, single-nucleus RNA-seq (snRNA-seq) has been developed to investigate cells that are too fragile or difficult to dissociate into single cells [8, 9]. Yet, ambient RNA contamination is likely more common in snRNA-seq than in scRNA-seq because the nuclei extraction procedure would cause many RNAs in the cytoplasm to be released into the solution. Although enzymatic degradation is theoretically possible to remove ambient RNAs, it is often too challenging to perform experimentally, especially for snRNA-seq, because endogenous RNAs are difficult to protect against degradation. Hence, ambient RNA contamination needs to be corrected in a post hoc manner in most cases.

Various experimental and computational strategies have been developed to correct the contamination in scRNA-seq and snRNA-seq data. Sanchez et al. developed an experimental approach that uses spike-in cells as a reference to correct the contamination [6]. However, this approach complicates the experimental procedure and has not been integrated into common commercial platforms. Several computational methods have been developed for decontamination, including SoupX [5], CellBender [10], and scAR [11], whose common idea is to first estimate the distribution of ambient RNA levels from empty droplets and then use the estimated distribution to correct the gene expression levels in cells. However, since SoupX, CellBender, and scAR require empty-droplet data, they are inapplicable to processed data in which empty droplets have been removed. Although another computational method, DecontX [4], does not require empty-droplet data, it and the three above methods alter all genes’ expression levels, possibly leading to an over-correction of the genes that did not cause the contamination. Such over-correction, especially for lowly expressed genes, will likely result in the missingness of informative genes in relevant cell types. However, the field lacks a comprehensive evaluation of computational decontamination methods for correcting genes at varying contamination levels.

**Fig. 1:**
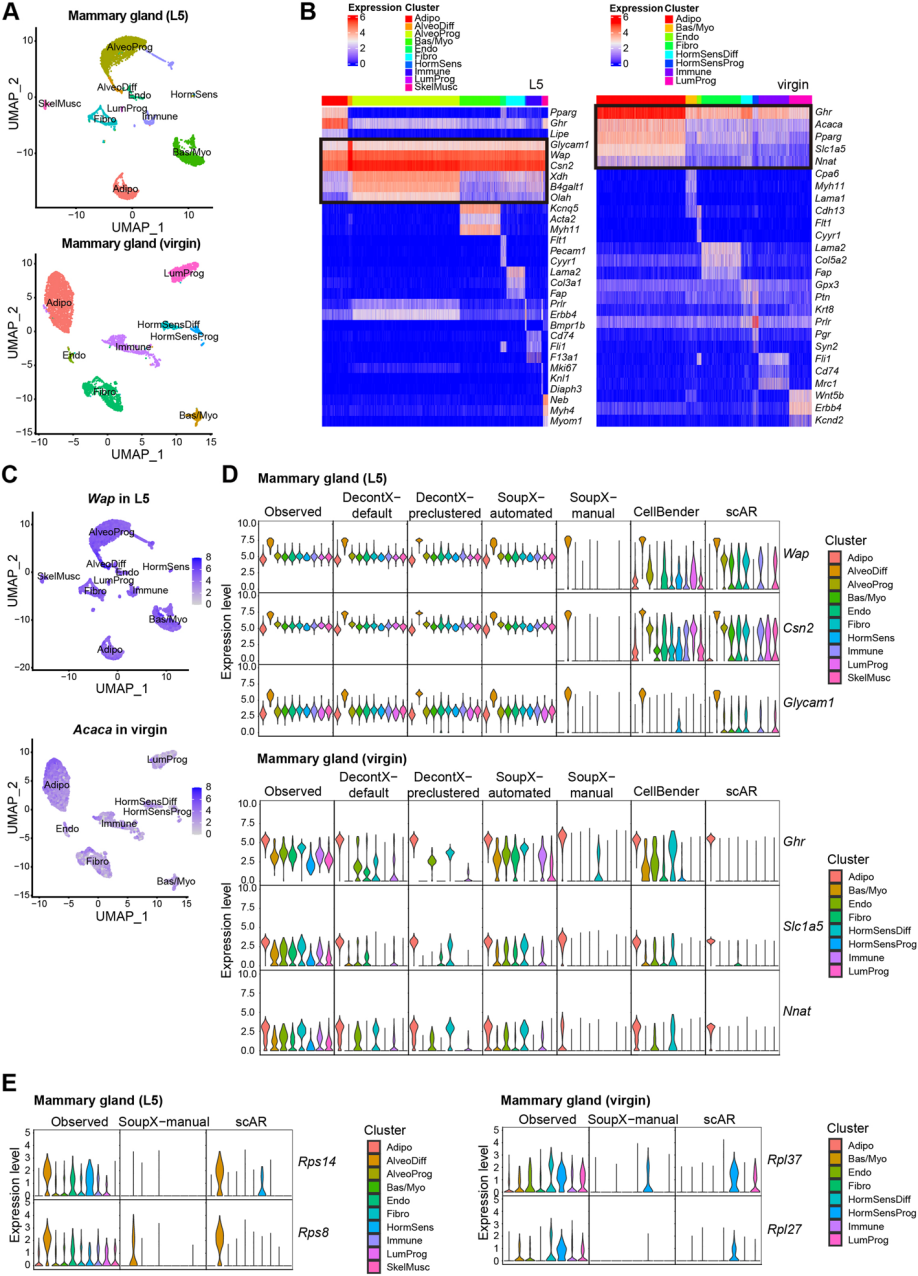
Performance evaluation of existing methods on correcting contaminated mammary gland snRNA-seq data. **A** The cell clusters identified in L5 and virgin mammary gland datasets are shown in UMAP plots. **B** Heatmap of the expression of selected marker genes in L5 and virgin mammary gland datasets. Notably, highlighted genes supposed to express exclusively in a cluster are widely detected in all the cells. **C** The expression of *Wap* and *Acaca* in the nucleus are shown in UMAP plots. **D**, **E** The violin plots show the normalized expression levels of the selected marker genes (**D**) and housekeeping genes (**E**) before and after correction using the indicated methods by the default Seurat (V3). Adipo, adipocytes; AlveoProg, alveolar progenitors; AlveoDiff, differentiated alveolar cells; Bas/Myo, basal cells/myoepithelial cells; Endo, endothelial cells; Fibro, fibroblasts; HormSens, hormone sensing cells; HormSensDiff, differentiated hormone sensing cells; HormSensProg, hormone sensing progenitors; Immune, immune cells; LumProg, luminal progenitors; SkelMusc, skeleton muscle cells

In this study, we performed snRNA-seq assays in mouse mammary glands at the virgin and lactation stages. In our snRNA-seq datasets, we observed sample-specific contamination by ambient RNAs. To correct the contamination, we applied the above computational methods but found that DecontX and CellBender exhibited an under-correction of highly contaminating genes, while SoupX and scAR over-corrected many genes, including housekeeping genes (Fig. 1).

Motivated by this result, we developed scCDC (single-cell Contamination Detection and Correction), which first detects the “contamination-causing genes,” which encode the most abundant ambient RNAs, and then only corrects these genes’ measured expression levels. We show that scCDC successfully corrected the contamination in our in-house snRNA-seq datasets. Moreover, scCDC improved the accuracy of identifying cell-type marker genes and constructing gene co-expression networks. Compared with DecontX, SoupX, CellBender, and scAR on synthetic datasets and real datasets, scCDC excelled in robustness and decontamination accuracy for correcting highly contaminating genes, while it avoids over-correction for lowly/non-contaminating genes. Not requiring empty-droplet data, scCDC has general applicability to all processed scRNA-seq and snRNA-seq datasets in public repositories. In addition, scCDC can be used in combination with DecontX to remove the remaining low contamination not caused by the contamination-causing genes scCDC identifies, by leveraging the complementary advantages of the two methods.

## Results

### Ambient RNA contamination in snRNA-seq data of mouse mammary glands

The mammary gland is a unique mammalian organ whose sole function is to feed the young. Hence, the mammary gland undergoes dramatic developmental changes during pregnancy and lactation. To investigate mammary gland development, several studies have performed scRNA-seq on epithelial cells of mammary glands [12–15]. However, the development of mammary glands also requires the interplay between epithelial cells and cells in the niche, including adipocytes, fibroblasts, and immune cells [16–18].

Instead of scRNA-seq, we employed snRNA-seq to profile a complete cellular map of virgin and lactating (lactation day 5, denoted by L5) mouse mammary glands. In addition to epithelial cells and subsets of luminal and basal cells, we successfully identified adipocytes, fibroblasts, and immune cells, which had not been efficiently captured by previous scRNA-seq studies (Fig. 1A). However, we found several well-known cell-type marker genes unexpectedly detected in nearly all cell types. For example, the genes *Wap* and *Csn2* encode the whey acidic and casein proteins, respectively. Previous studies employing genetic reporter mouse strain and RNA in situ hybridization demonstrated that *Wap* and *Csn2* are expressed exclusively in the differentiated alveolar epithelial cells (AlveoDiff) during lactation [19, 20], whereas the gene *Acaca*, which encodes the acetyl-CoA carboxylase for fatty acid synthesis, is expected to be expressed exclusively in adipocytes (Adipo). Surprisingly, however, these genes’ mRNAs were also detected in nearly all the other cell types. Similarly, AlveoDiff marker *Glycam1* and Adipo marker *Ghr*, *Slc1a5*, and *Nnat* were detected globally in lactating and virgin datasets, respectively (Fig. 1B, C). These data suggest the presence of systematic contamination caused by ambient RNAs. In addition, comparing the data generated from experiments with or without the nuclei sorting procedure, we observed that contamination was slightly lower but still noticeable when nuclei sorting was added (Additional file 1: Fig. S1).

### Performance evaluation of four existing decontamination methods on in-house mouse mammary gland snRNA-seq datasets

The four aforementioned computational methods—DecontX, SoupX, CellBender, and scAR—were developed to correct contaminated scRNA-seq and snRNA-seq data [4–6, 10, 11]. Here, we evaluated the performance of these methods in correcting our in-house snRNA-seq data of mouse mammary glands.

Applied to the lactating dataset, DecontX barely removed any contamination of AlveoDiff markers *Wap*, *Csn2*, and *Glycam1* in both the “default” mode (DecontX-default) and the “pre-clustered” mode that takes user-specified cell clusters (DecontX-preclustered). Similarly, SoupX failed to correct the three genes’ contamination in the “automated” mode (SoupX-automated), and only SoupX “manual” mode (SoupX-manual, which takes user-defined contamination-causing genes) achieved a reasonable correction performance. Moreover, CellBender and scAR under-corrected the contamination of the three genes (Fig. 1D, upper panel).

Applied to the virgin dataset, only scAR successfully corrected the contamination of Adipo markers *Ghr*, *Slc1a5*, and *Nnat*. Specifically, SoupX-automated failed to correct these three genes’ contamination; DecontX-default, DecontX-preclustered, and CellBender all under-corrected these three genes’ contamination; SoupX-manual under-corrected *Ghr*’s contamination (Fig. 1D, lower panel).

Since DecontX, SoupX, CellBender, and scAR alter all genes’ counts, we also checked how they changed the counts of the genes other than the above cell-type marker genes. Although SoupX-manual and scAR had less of an under-correction issue for cell-type marker genes, they undesirably removed the counts of some housekeeping genes, such as *Rps14*, *Rps8*, *Rpl37*, and *Rplp27*, in multiple cell types (Fig. 1E). Examining the counts of 66 housekeeping genes before and after each method’s correction, we found that SoupX-manual and scAR undesirably removed the counts of many housekeeping genes in more than 95% of cells (Additional file 1: Fig. 2). These results revealed the over-correction issue of SoupX-manual and scAR.

**Fig. 2:**
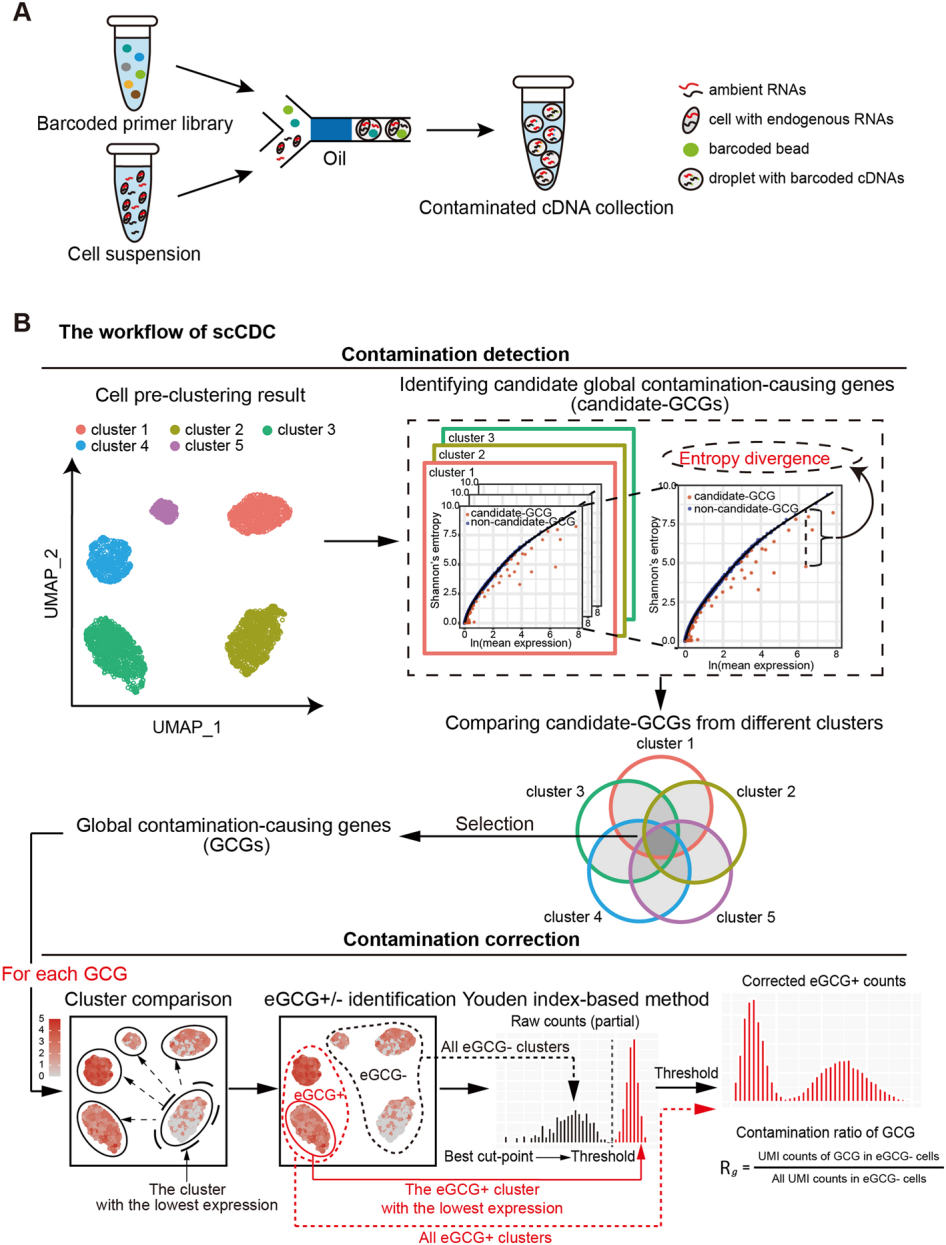
An overview of scCDC workflow. **A** The diagram of contamination shows ambient RNAs cause contaminated profiles for scRNA-seq (or snRNA-seq). **B** Workflow of scCDC. The theoretical entropy-expression curves of endogenous RNAs are simulated and the divergence of observed and expected entropy are calculated. Genes with significant entropy divergence were selected in each cluster and the common genes were defined as GCGs. For contamination correction, the clusters of cells do or do not express endogenous GCGs were first defined (eGCG + and eGCG − cells). Youden index-based method was used to correct the contaminated counts based on the count distribution of the eGCG + cluster with the lowest expression and the count distribution of all eGCG − clusters. The contamination ratio of a GCG is calculated based on the proportion of the GCG’s total UMI count among all genes’ total UMI count in eGCG − cells (details in “Methods”)

Taken together, our evaluation results show that DecontX, SoupX-automated, and CellBender under-corrected the genes that caused extensive contamination (usually cell-type marker genes), while SoupX-manual and scAR over-corrected some other genes, including housekeeping genes.

### Overview of scCDC

Motivated by these decontamination methods’ unsatisfactory performance on our in-house datasets, in particular, SoupX-manual and scAR’s over-correction, we investigated the composition of ambient RNA contamination; that is, whether the contamination was caused by a small number of genes or all genes globally. Previous research has used RNA abundance in empty droplets to estimate ambient RNA contamination [5, 11]. Accordingly, we examined the empty droplets in our in-house datasets, public scRNA-seq datasets of PBMCs (peripheral blood mononuclear cells) and pancreas [6], and a public snRNA-seq dataset of skeletal muscle [21]. We found in each dataset that the ambient RNAs in the empty droplets are mainly contributed by a small group of genes, which we refer to as “super-contaminating genes” in Additional file 1: Fig. S3 and Method Appendix [22] (See Additional file 1). For example, in our in-house lactating mammary gland dataset, RNAs in the empty droplets were dominantly from *Wap* and other milk protein-coding genes, such as *Csn2* and *Csn1s1*, while the counts of housekeeping genes *Rps14* and *Rps8* were less than 1% of *Csn2*’s. Similarly, in our in-house virgin mammary gland dataset, the majority of RNAs in the empty droplets were from a small group of genes, including *Gm42418*, *Malat*, and *Ghr*, while housekeeping genes like *Rpl37* and *Rplp27* only contributed small amounts of RNAs (Additional file 1: Fig. S3). These results suggest that ambient RNA contamination is mainly caused by a small number of genes.

Based on this finding, we devised a gene-specific strategy to identify contamination-causing genes without requiring empty droplets and correct only these genes’ contamination. In contrast to the global strategy used by the existing correction methods to correct all genes’ possible contamination, our gene-specific strategy can better avoid the under-correction of contamination-causing genes and the over-correction of other genes. Following this strategy, we developed a new method, scCDC, which has two functionalities: contamination detection and correction.

A contamination-causing gene has abundant ambient RNAs, so its observed count in a droplet is the sum of the counts from its endogenous and ambient RNAs (Fig. 2A). The method scCDC is designed to identify each of such genes first and then correct the gene’s observed counts. Because the gene’s ambient RNAs are abundant but less variable in droplets, the existence of ambient RNA counts would deflate the entropy of the gene’s observed counts in droplets. Following this rationale, scCDC’s contamination detection functionality consists of three steps (Fig. 2B). (Note that scCDC requires cells to be pre-clustered, an issue we will discuss in the Discussion and Method Appendix. See also Additional file 1.) First, under the assumption that most genes produce little or no ambient RNAs (defined as “endogenous genes”), scCDC estimates the expected entropy-expression curve of endogenous genes within each cell cluster (see “Methods”). Second, in each cell cluster, scCDC calculates the “entropy divergence,” defined as a gene’s expected entropy (which is calculated based on the gene’s expression and the expected entropy-expression curve) minus its observed entropy, to represent the gene’s contamination level. Third, scCDC identifies the “global contamination-causing genes” (GCGs; details in “Methods”) as the genes with statistically significant entropy divergences in more than 50% of the cell clusters. (Note that 50% is the default value of the “restriction factor,” a tuning parameter that can be user-specified: the larger the restriction factor, the fewer GCGs scCDC identifies; we set the default restriction factor to 50% based on empirical results—details are in the “Methods,” Method Appendix, and Additional file 1).

After detecting the GCGs, scCDC’s contamination correction functionality corrects these GCGs’ observed counts. For each GCG, scCDC corrects the observed counts in two steps. First, scCDC finds the cell clusters in which the GCG is unlikely expressed and labels these clusters as eGCG − and the remaining clusters as eGCG + (Fig. 2B). Technically, scCDC locates the cell cluster in which the GCG has the lowest mean expression; then, scCDC groups the cell cluster with similar clusters, with the similarity defined based on the GCG’s count distribution in each cluster (details in “Methods”). The justification is that the GCG should have similar count distributions in the clusters where it is unexpressed because its count distributions in these clusters are all determined by its ambient RNAs. Second, scCDC corrects the GCG’s counts in the eGCG + clusters by subtracting from the GCG’s observed counts a positive value, which is determined by the Youden index-based method using two distributions: (1) the GCG’s count distribution in the eGCG + cell cluster that has the highest similarity with the eGCG − clusters and (2) the GCG’s count distribution in the pooled eGCG − cell clusters (Fig. 2B and “Methods”).

In addition, for each GCG, scCDC calculates the “contamination ratio” (defined as the GCG’s total count in the eGCG − cells over the total count of all genes in the eGCG − cells) to evaluate the level of contamination caused by the GCG (Fig. 2B; details in “Methods”).

### Contamination detection by scCDC for scRNA-seq or snRNA-seq data

We first validated the contamination detection functionality of scCDC using simulation. We simulated an uncontaminated PBMC scRNA-seq dataset using a realistic simulator scDesign2 [19] and then artificially contaminated the data with three contaminating genes *LYZ*, *S100A8*, and *S100A9*. The three genes’ entropy divergences increased in all cell clusters after artificial contamination was added (Additional file 1: Fig. S4A), and their mean entropy divergences (across clusters) correlated positively with the artificial contamination levels (i.e., the proportions of “ambient” counts) (Additional file 1: Fig. S4B). All three genes were successfully identified by scCDC as GCGs, and their contamination ratios calculated by scCDC correlate positively with their artificial contamination levels, suggesting that the contamination ratio is a reasonable measure of a gene’s contamination level (Additional file 1: Fig. S4C). These results supported scCDC’s contamination detection functionality.

Next, we applied scCDC to our two in-house mammary gland datasets and thirteen public scRNA-seq and snRNA-seq datasets of various mouse and human tissues (Additional file 2: Table S1) [6, 21, 23–30]. From these datasets, scCDC identified various numbers of GCGs. Of note, the identified GCGs are generally the most abundant genes in the empty droplets (Additional file 1: Fig. S3) and were detected in all cell types, supporting the contamination detection functionality of scCDC. More importantly, the GCGs are mostly known as cell-type marker genes instead of housekeeping genes, so the detection and decontamination of GCGs is necessary for cell-type discovery. Below, we describe the GCG detection results of scCDC on five exemplar datasets.

**Fig. 3:**
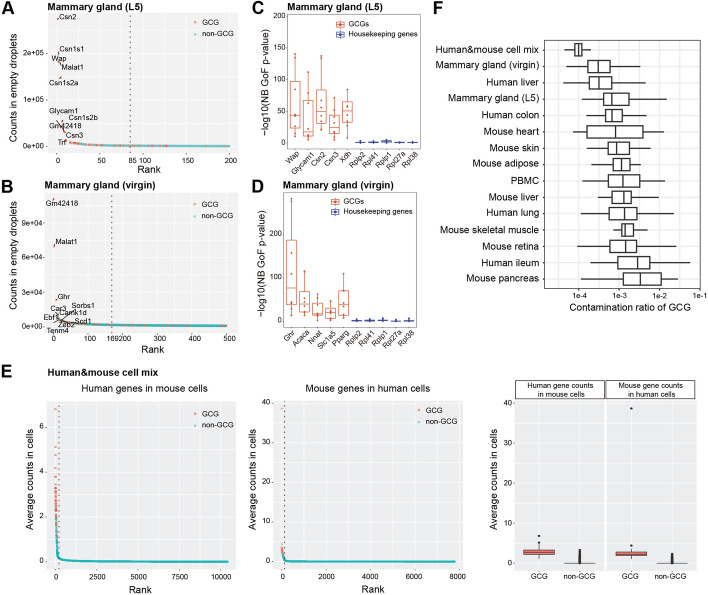
Contamination detection by scCDC in snRNA-seq and scRNA-seq datasets. **A**, **B** The plots show the counts vs rank in empty droplets in the snRNA-seq datasets in mammary glands. The top ranked 200 and 500 genes are shown. Selected GCGs are highlighted and labeled on the plot. The dash line separated the “super-contaminating genes” and the other genes (details in Method Appendix. See Additional file 1). **C**, **D** Distribution of GCGs is significantly deviated from negative binomial (NB) distribution. Box plots of the *p*-values of NB distribution goodness-of-fit test of GCGs and housekeeping genes. **E** scCDC identifies highly contaminating genes in the “barnyard” scRNA-seq dataset of mixed human 293 T cells and mouse 3T3 cells. The scatter plots show the average counts vs. ranks of cross-species contaminating genes in the indicated cells. The average cross-species contaminative counts of GCG and non-GCGs are shown in the boxplots on the right. **F** Box plots show the contamination ratios of GCGs in the indicated datasets

From our in-house snRNA-seq datasets of mouse lactating and virgin mammary glands, scCDC identified 72 and 106 GCGs, respectively (Additional file 2: Table S1). The identified GCGs included the top contaminating genes in empty droplets (Fig. 3A, B). Bulk RNA-seq data of the same tissues confirmed the high expression of these GCGs (Additional file 1: Fig. S5A, B). Moreover, we observed that the count distributions of these GCGs deviated significantly from the negative binominal (NB) distribution, i.e., the expected count distribution in a cell cluster without contamination [19, 20] (Fig. 3C,D), confirming the heavy contamination caused by these genes. These GCGs included known cell-type marker genes. For example, AlveoDiff marker genes, including *Wap*, *Csn2*, and *Glycam1*, were identified as GCGs in the lactating mammary gland; Adipo marker genes, such as *Acaca*, *Ghr*, *Slc1a5*, and *Nnat*, were found as GCGs in the virgin mammary gland (Fig. 3A and Additional file 2: Table S1).

In a highly contaminated scRNA-seq dataset of mouse pancreas [6], insulin encoding genes *Ins1* and *Ins2* should be expressed exclusively and abundantly in Beta cells [31], but they were unexpectedly detected in almost all cells (Additional file 1: Fig. S6A). Applying scCDC to the dataset, we identified 12 GCGs, including *Ins1* and *Ins2* (Additional file 1: Fig. S6B and Additional file 2: Table S1). Among the GCGs identified by scCDC, 11 GCGs were confirmed as highly expressed by bulk RNA-seq (Additional file 1: Fig. S6C; the only GCG not found in the bulk RNA-seq data was a pseudogene). Similar to our observation from our in-house mammary gland datasets, the GCGs’ count distributions in each cell cluster deviated significantly from the NB distribution; in contrast, the housekeeping genes’ count distributions in each cell cluster were approximately NB (Additional file 1: Fig. S6D).

Applying scCDC to a commonly used benchmark “barnyard” dataset of mixed human 293 T and mouse 3T3 cells [4], we found that the cross-species contamination was dominantly from the GCGs scCDC identified, although the GCGs had low contamination ratios in general (Fig. 3E and Additional file 1: Fig. S7).

In the PBMC-4 K dataset, it has been widely reported that the gene *LYZ*, a marker gene of mononuclear phagocytes (MNPs), is a contamination-causing gene [4]. Applied to the dataset, scCDC successfully identified *LYZ* as a GCG (Additional file 1: Fig. S8A).

Notably, examining the contamination ratios of the GCGs scCDC identified from these datasets, we found that the contamination ratios could vary significantly in one dataset (Fig. 3F), consistent with the fact that genes’ ambient RNA levels could vary in a wide range in empty droplets (Additional file 1: Fig. S3). Moreover, the mean contamination ratio of GCGs varied greatly from dataset to dataset. In particular, the mean contamination ratio of GCGs was significantly lower in the “barnyard” dataset, which was commonly used for benchmarking decontamination methods, than in the other 14 datasets (Fig. 3F). In contrast, the mouse pancreas dataset had the highest mean contamination ratio of GCGs (Fig. 3F). Hence, it is insufficient to use the “barnyard” dataset only for benchmarking decontamination methods because its contamination level is not representative of many datasets.

### Four existing methods under-corrected GCGs or over-corrected lowly/non-contaminating genes

Regarding contamination correction, we compared scCDC with four existing methods: DecontX, SoupX, CellBender, and scAR. Table 1 summarizes the five methods in five aspects: (1) whether a method can work without empty-droplet data, (2) whether a method can run with CPU only, (3) whether a method corrects all genes, (4) whether a method evaluates a gene’s contamination within each cell cluster, and (5) whether a method requires preclustering. For scCDC, the answers are yes, yes, no, yes, and yes.

**Table 1:** Comparison of scCDC with DecontX, SoupX, CellBender and scAR in five aspects

	**Only a filtered gene-by-cell matrix needed (no empty droplets)**	**Only CPU needed**	**Data correction**	**Contamination evaluation in individual cluster**	**Preclustering required**
scCDC	√	√	GCGs only	√	√
DecontX-default	√	√	Globally	×	×
DecontX-preclustered	√	√	Globally	×	√
SoupX-automated	×	√	Globally	×	×
SoupX-manual	×	√	Globally	×	×
CellBender	×	×	Globally	×	×
scAR	×	√	Globally	×	×

Using eight out of the fifteen scRNA-seq or snRNA-seq datasets we applied scCDC to (Additional file 3: Table S2), we evaluated the four existing methods’ decontamination efficacy on the GCGs detected by scCDC. We were only able to apply the four methods to the eight datasets that contained empty droplets because SoupX, CellBender, and scAR required empty droplets as input.

**Fig. 4:**
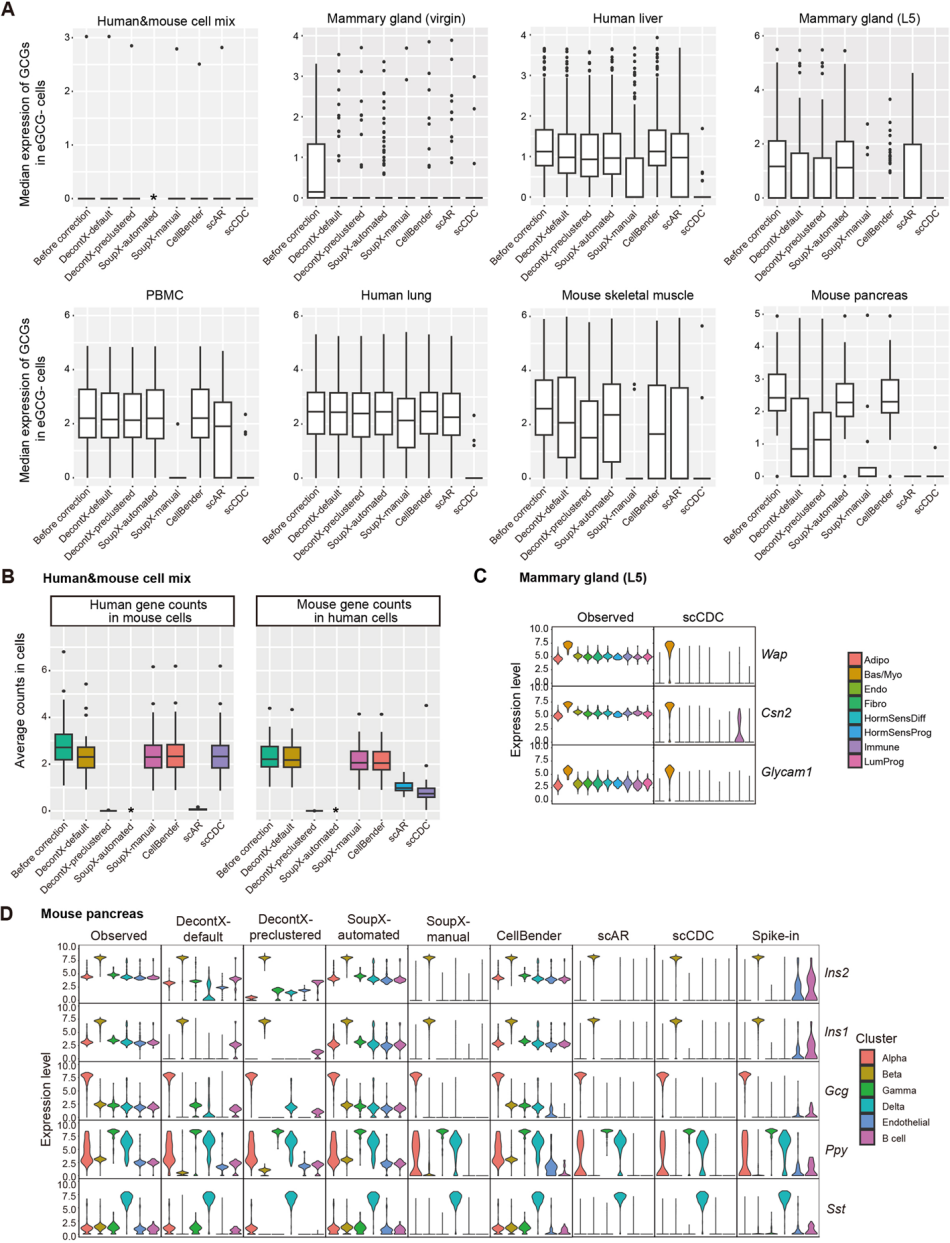
scCDC is a robust method for correcting highly contaminating GCGs in real datasets. **A** The box plots show the medium expression of GCGs in eGCG − cells before and after correction in the indicated datasets. **B** Box plots show the counts of GCGs before and after correction by the indicated method in the “barnyard” scRNA-seq dataset of mixed human 293 T cells and mouse 3T3 cells. Of note, SoupX-automated fails to run on the data. *, no output. **C** scCDC efficiently corrects highly contaminating genes in the snRNA-seq dataset of the lactating mammary gland. The violin plots show the normalized expression levels of the indicated GCGs before and after correction by scCDC. **D** DecontX and SoupX-automated displayed under-correction in the scRNA-seq data in mouse pancreas islet with spike-ins. The violin plots show the normalized expression levels of the indicated GCGs before and after correction using the indicated methods. The plots were made by the default Seurat (V3)

We first verified the decontamination efficacy of scCDC on the GCGs, which we had confirmed to be likely contaminated in our previous analysis. For each GCG, we calculated its median expression level in the eGCG − cells after the correction (Fig. 4A), and a low level would indicate an effective decontamination of the GCG. The results showed that scCDC effectively corrected the GCGs in all eight datasets (Fig. 4A). Since we previously observed the over-correction of some of the 66 housekeeping genes by SoupX-manual and scAR in our in-house mammary gland datasets, we examined if scCDC over-corrected the 66 housekeeping genes in the eight datasets. Since scCDC by design only corrects its detected GCGs, which did not include the housekeeping genes in general (Additional file 2: Table S1), we did not observe the over-correction of housekeeping genes by scCDC (Additional file 4: Table S3).

Next, we evaluated the decontamination efficacy of the four existing methods for the GCGs detected by scCDC in the eight datasets (Fig. 4A). The results showed that the four methods had overall good decontamination efficacy for the GCGs exhibiting low contamination ratios, such as the GCGs in the “barnyard” dataset (Fig. 4A). However, we observed that DecontX, SoupX-automated, and CellBender under-corrected the GCGs exhibiting high contamination ratios, such as the GCGs in the human liver and mouse pancreas datasets (Fig. 4A). These results suggested that DecontX, SoupX-automated, and CellBender provided insufficient correction for highly contaminating GCGs (Additional file 4: Table S3). Compared to DecontX, SoupX-automated and CellBender, although SoupX-manual and scAR had a much less under-correction issue, they still under-corrected a subset of GCGs in the lactating mammary gland and mouse pancreas datasets (Fig. 4A and Additional file 4: Table S3).

Based on our previous observation of the over-correction issue of SoupX-manual and scAR, we examined if the two methods over-corrected any of 66 housekeeping genes in the eight datasets. The results showed that SoupX-manual over-corrected some housekeeping genes and lowly contaminating GCGs in four datasets, and scAR exhibited an over-correction of some of these genes in five datasets (Additional file 4: Table S3).

In summary, Additional file 4: Table S3 lists the observed under-correction of GCGs and the over-correction of housekeeping genes and lowly contaminating GCGs by each of the five methods in the eight datasets.

Below we discuss in detail the decontamination performance of the five methods on five exemplar datasets, ordered by an increasing decontamination level (measured as the mean contamination ratio of the GCGs): (1) the “barnyard” dataset of mixed human 293 T and mouse 3T3 cells, (2) the human liver dataset, (3) our in-house data from the lactating mammary gland, (4) the PBMC-4 K dataset, and (5) the mouse pancreas dataset.

In the “barnyard” dataset with lowly contaminating GCGs only [4], DecontX-preclustered performed the best among the methods, removing almost all the contamination from the dataset; however, DecontX-default barely corrected the contamination. Interestingly, scAR removed the contamination completely in mouse cells but not in human cells (Fig. 4B), and it over-corrected lowly expressed mouse genes in mouse cells and housekeeping genes in both mouse and human cells (Additional file 1: Fig. S9). For SoupX, SoupX-automated could not successfully run on this dataset. Notably, scCDC was secondary to DecontX-preclustered in correcting the contamination in human cells, and it was secondary to DecontX-preclustered and scAR in decontaminating mouse cells (with performance similar to SoupX-manual and CellBender) (Fig. 4B).

In the liver dataset [32], contamination was detected for ambient RNAs of *HBB* from erythroid cells and those of *ALB* and *APOA2* from hepatocytes (Additional file 1: Fig. S10A). SoupX-manual and scAR provided sufficient correction for these genes, while DecontX and CellBender exhibited an under-correction (Additional file 1: Fig. S10B). For housekeeping genes, scAR had a slight over-correction (Additional file 1: Fig. S10C). In contrast, scCDC effectively corrected the contamination of *HBB*, *ALB*, and *APOA2*, and it did not exhibit an over-correction of housekeeping genes (Additional file 1: Fig. S10B).

As previously shown in Fig. 1, in our snRNA-seq dataset of the lactating mammary gland, DecontX, SoupX-automated, and CellBender under-corrected the contamination on AlveoDiff marker genes *Wap*, *Csn2*, and *Glycam1.* Although SoupX-manual and scAR provided sufficient correction for the three genes, they over-corrected a few housekeeping genes. Unlike these methods, scCDC effectively corrected *Wap*, *Csn2*, and *Glycam1* (Fig. 4C) but did not over-correct the housekeeping genes (Additional file 1: Fig. S2).

In the PBMC-4 K dataset, all five methods successfully corrected the contamination of *LYZ* (Additional file 1: Fig. S8B). Nevertheless, we observed an under-correction of the highly contaminating gene *CD74* by DecontX, SoupX-manual, and CellBender (Additional file 1: Fig. S8B). In addition, we found a severe over-correction of housekeeping genes by SoupX-manual and scAR (Additional file 1: Fig. S8C).

Lastly, we examined the five methods on the mouse pancreas dataset that exhibited the highest level of contamination among the eight datasets (Fig. 3F). In the study that generated this dataset, Sanchez et al. estimated genes’ contamination fractions using spike-in cells in the experiment and corrected the data based on the estimated contamination fractions [6]. Accordingly, we used the spike-in data as a reference. In our results, DecontX, SoupX-automated, and CellBender failed to sufficiently correct the highly contaminating GCGs, such as the known Beta cell marker *Ins2*, Alpha cell marker *Gcg*, and Delta cell marker *Sst* (Fig. 4D) [6]. In contrast, SoupX-manual and scAR removed the contaminative counts of GCGs, resulting in even cleaner decontamination results than the spike-in-based correction in the original study, which did not remove all contaminative counts in the endothelial cells and B cells (Fig. 4D and Additional file 1: Fig. S11A). Among these methods, SoupX-manual and scAR achieved the overall best correlations between their corrected counts and the spike-in-based corrected counts (Additional file 1: Fig. S11B). Again, we assessed if SoupX-manual and scAR over-corrected any lowly or non-contaminating genes in the dataset. Similar to the observations in our in-house snRNA-seq datasets and PBMC dataset, SoupX-manual and scAR, but not the other methods, undesirably removed the counts of many housekeeping genes in more than 95% of cells (Additional file 1: Fig. S11C). In addition, we found that the counts of *Irx1* and *Irx2*, the marker genes of Alpha cells [33], were removed by scAR in Alpha cells (Additional file 1: Fig. S11D), while these two genes were not identified as GCGs by scCDC and showed very low contamination levels in empty droplets (Additional file 1: Fig. S6B). These results were also in line with a previous report about potential gene loss caused by scAR [11]. Similar to the results in other datasets, scCDC provided sufficient correction for the highly contaminating cell-type marker genes like *Ins1*, *Ins2*, and *Gcg* (Fig. 4D), and it did not over-correct the lowly or non-contaminating genes like *Irx1* and *Irx2* (Additional file 2: Table S1).

Collectively, our thorough analysis of real datasets has revealed the under-correction of highly contaminating GCGs, including cell-type marker genes, by DecontX, SoupX-automated, and CellBender. Additionally, we have observed the over-correction of lowly/non-contaminating genes, particularly housekeeping genes, by SoupX-manual and scAR. Addressing these limitations, scCDC provided robust correction on highly contaminating GCGs and avoided over-correction on the other genes.

### Simulation confirmed the effectiveness of scCDC in correcting highly contaminating genes and suggested a combined use of scCDC and DecontX

**Fig. 5:**
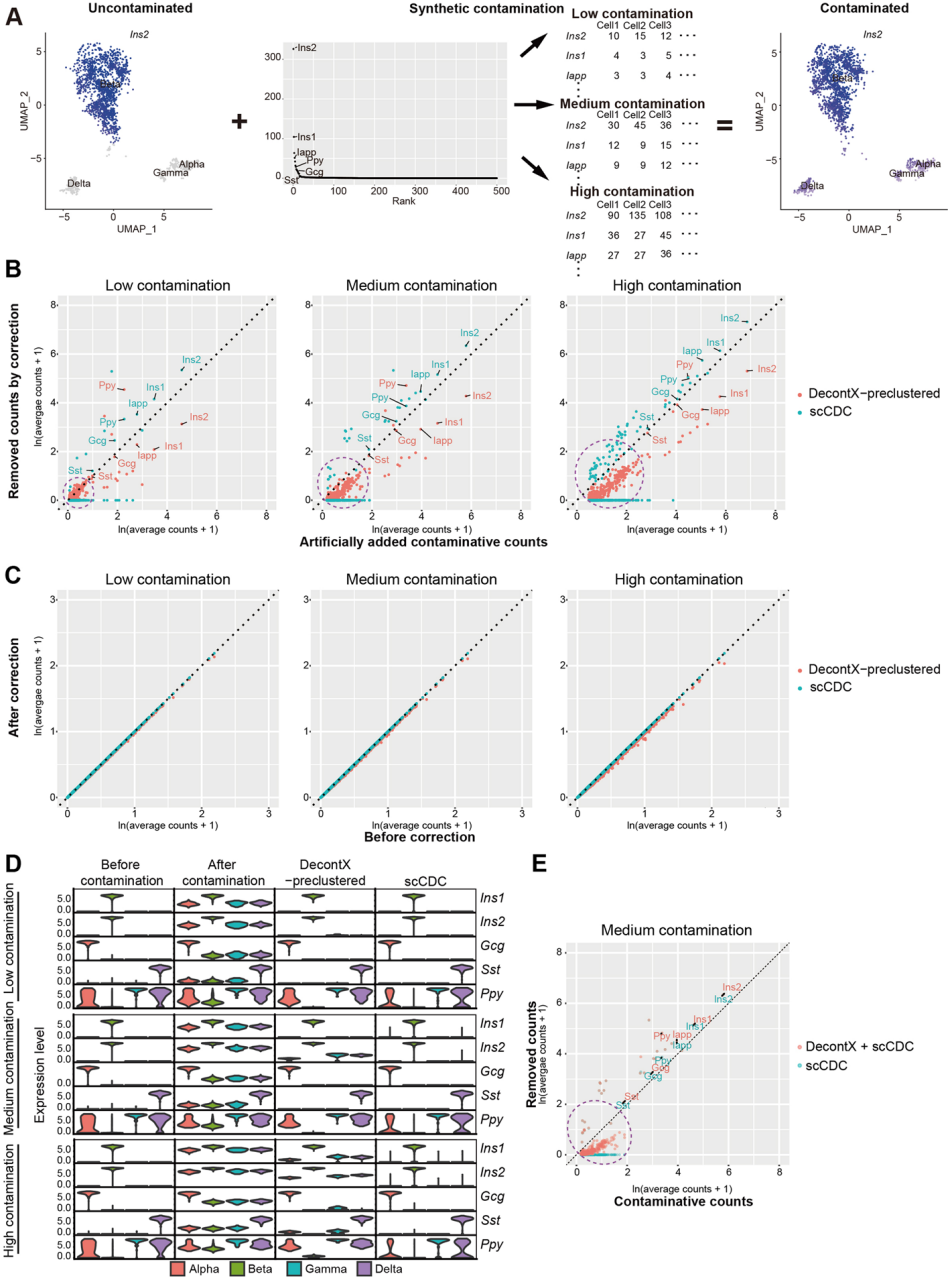
Simulation confirms the effectiveness of scCDC in correcting highly contaminating genes and suggests a combined use of scCDC and DecontX. **A** The diagram shows the simulation strategy of artificial contamination with varying levels. Artificial contamination of 500 genes at low, medium, and high levels is added to the uncontaminated simulated data. **B** Scatter plots show the artificial contaminative counts vs. corrected counts of the 500 artificial contaminating genes in (**A**) by indicated methods. Top contaminating genes are labeled. Lowly contaminating genes are circled. **C** DecontX slightly over-corrects non-contaminating genes in highly contaminative data. Scatter plots show the counts before and after correction of the genes that are not artificially contaminated. **D** The violin plots show the normalized expression levels of indicated top GCGs before and after correction by different computational methods. **E** DecontX helps scCDC correct lowly contaminating genes. Scatter plots show the artificial contaminative counts vs. corrected counts of the 500 artificial contaminating genes as in (**B**)

To better validate the robustness of scCDC in a scenario with ground truths, we simulated artificially contaminated pancreas datasets by mimicking the real data in Sanchez et al.’s study [6]. First, we simulated an uncontaminated single-cell dataset by a realistic simulator scDesign2 [34] trained on the filtered, spike-in-corrected real data (Additional file 1: Fig. S12A). Second, to mimic the real-data contamination of genes with varying contamination levels, we simulated artificial contaminative counts of the 500 genes that had the most counts in the empty droplets of the real data. Specifically, the 500 genes’ simulated contaminative levels (i.e., the contaminative level of each gene is the mean parameter of the NB distribution for contaminative counts) were proportional to their total counts in empty droplets, under each of three contamination scenarios (low, medium, and high) (Fig. 5A). Then we generated contaminated datasets with varying contamination levels by adding each of the 500 genes’ uncontaminated counts (simulated in the first step) and artificial contaminative counts (simulated in the second step). We used these contaminated datasets to evaluate the robustness of scCDC in correcting highly and lowly contaminating genes. We also compared scCDC with DecontX-preclustered, the better-performing mode of DecontX and the only one that does not require empty droplets among the four existing methods.

In the low contamination scenario, DecontX-preclustered successfully removed the artificial contaminative counts, although it had an under-correction of the highly contaminating genes. In the medium and high contamination scenarios, DecontX exhibited an obvious under-correction for all contaminating genes (Fig. 5B, D). These results confirmed our finding that DecontX is sensitive to the contamination levels of contaminating genes: it provides a sufficient correction for lowly contaminating genes but an insufficient correction for highly contaminating genes. Interestingly, DecontX also removed a small number of non-contaminating genes (i.e., over-correcting these genes) when the overall contamination level was high (Fig. 5C).

In contrast, under all three contamination scenarios, scCDC successfully identified highly contaminating genes as GCGs (Additional file 1: Fig. S12B) and effectively corrected their contaminative counts. Moreover, scCDC did not alter the counts of non-contaminating genes as designed (Fig. 5B-D). Consistent with what we observed from the aforementioned real datasets, scCDC was insensitive to the contamination levels of GCGs and provided an effective correction for GCGs (Fig. 5B–D). These results confirmed that scCDC is a robust correction method for highly contaminating genes.

We noticed that scCDC did not identify some lowly contaminating genes among the 500 artificial contaminating genes as GCGs, so it did not provide correction for these genes (circled in Fig. 5B, Additional file 1: Fig. S12B). Together with the results in the “barnyard” dataset of mixed human 293 T and mouse 3T3 cells (Fig. 4B), these results suggested that scCDC is more effective in the “highly contaminating” scenario than the “low-contaminating” scenario. Given the effectiveness of DecontX for correcting lowly contaminating genes, we explored the combined use of scCDC and DecontX (that is, DecontX is applied to the data corrected by scCDC) for their comparative advantages. Indeed, scCDC followed by DecontX corrected the counts of both highly and lowly contaminating genes (circled in Fig. 4E). These results suggested that the combined use of scCDC and DecontX could lead to more effective decontamination than each method alone. Based on our results in real and simulated datasets, we found that under-correction by DecontX occurred when a GCG’s contamination ratio exceeds 3.16 × 10^−4^. Therefore, we recommend employing the combined approach of scCDC and DecontX-preclustered for decontaminating datasets that contain GCGs with contamination ratios exceeding 3.16 × 10^−4^.

Furthermore, we used the simulated data to investigate if an iterative application of scCDC (i.e., applying scCDC again to its corrected data from the previous iteration) could improve the decontamination accuracy. Our results from three iterations showed that the number of GCGs dramatically decreased after the first iteration and became stable (Additional file 1: Fig. S13A). Of note, few genes were identified as GCGs in more than one iteration, and their corrected expression remained stable after the first iteration (Additional file 1: Fig. S13B). Meanwhile, housekeeping genes were not found as GCGs in all iterations, as expected (Additional file 1: Fig. S13C). Similar results were observed when scCDC was iteratively applied to our in-house dataset of lactating mammary glands (Additional file 5: Table S4). Hence, one iteration of scCDC (GCG detection followed by correction) is sufficient.

**Fig. 6:**
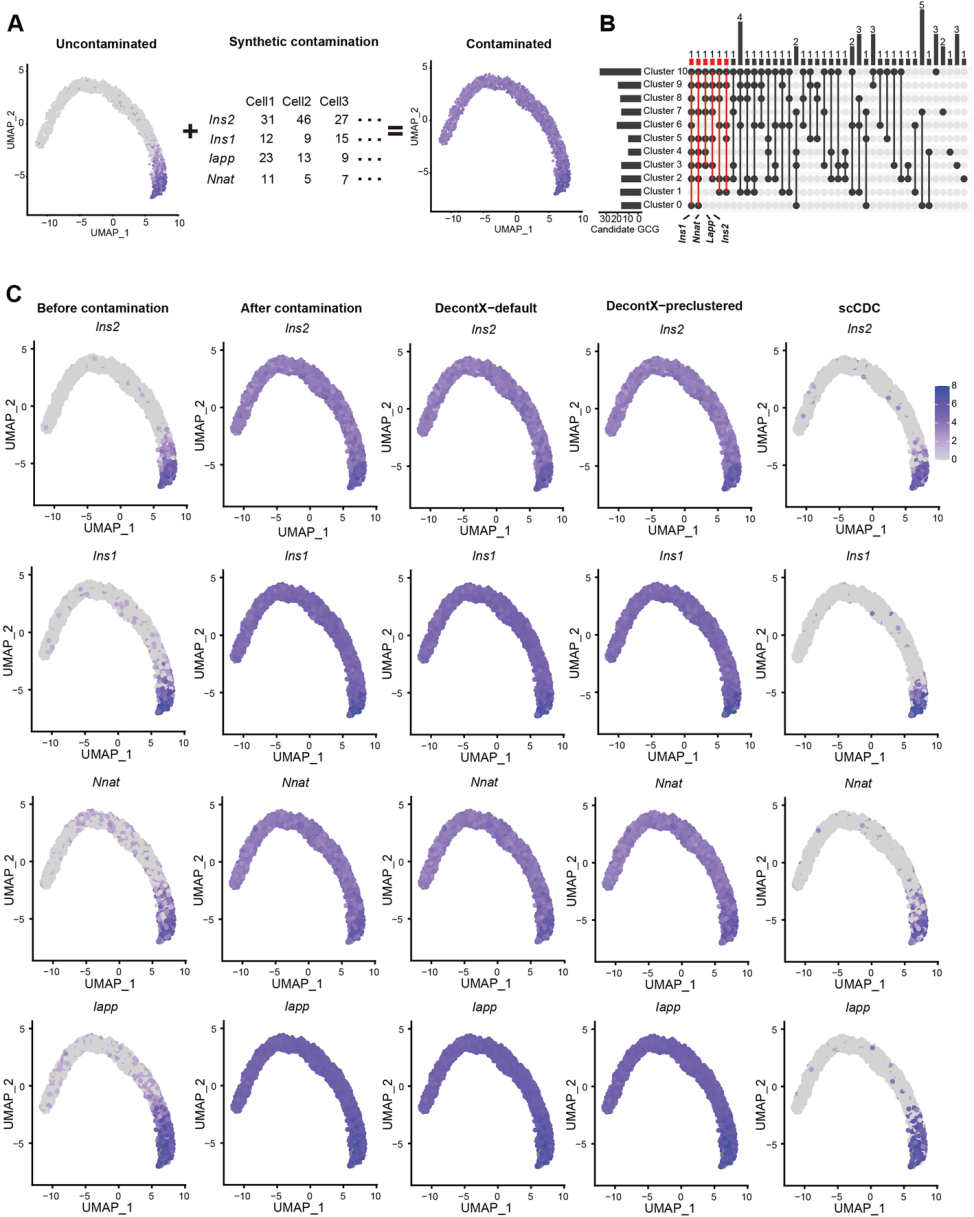
scCDC effectively corrects contamination in synthetic scRNA-seq trajectory dataset. **A** The diagram shows the simulation strategy of artificial contamination. **B** scCDC successfully identifies artificial contaminating genes. Upset plot shows the identification of GCGs from candidate GCGs in each cluster. GCGs are highlighted in red, and the artificial contaminating genes are labeled. **C** scCDC effectively corrects the artificial contamination. The expression of the artificial contaminating genes in uncontaminated, artificially contaminated, and corrected data are shown in the UMAP plots

Noting that scCDC requires the pre-clustering of cells, we further tested if scCDC is applicable to data that do not have clear cell cluster separation. First, we simulated an uncontaminated pancreas developmental trajectory dataset by scDesign3 [35] and artificially added contamination of four genes: *Ins2*, *Ins1*, *Nnat*, and *Iapp* (Fig. 6A). Based on default pre-clustering in Seurat, the cells were grouped into eleven clusters, from which scCDC identified six GCGs (Fig. 6B and Additional file 1: Fig. S14). Notably, the top four GCGs were the artificial contaminating genes (Fig. 6B). Moreover, scCDC successfully corrected the counts of the four GCGs (Fig. 6C). In contrast, DecontX failed to correct the data effectively (Fig. 6C). Second, we applied scCDC to a scRNA-seq dataset of developmental pituitary glands [36]. scCDC identified cell-type marker genes *TSHB*, *POMC*, and *HBG1* as GCGs and successfully corrected their contamination. In contrast, DecontX provided insufficient correction for *POMC* and *HBG1* (Additional file 1: Fig. S15). These results confirmed the robustness of scCDC in correcting contaminated data when cell clusters are less distinct.

### Application of scCDC improved cell-type marker gene profiling and gene network construction

**Fig. 7:**
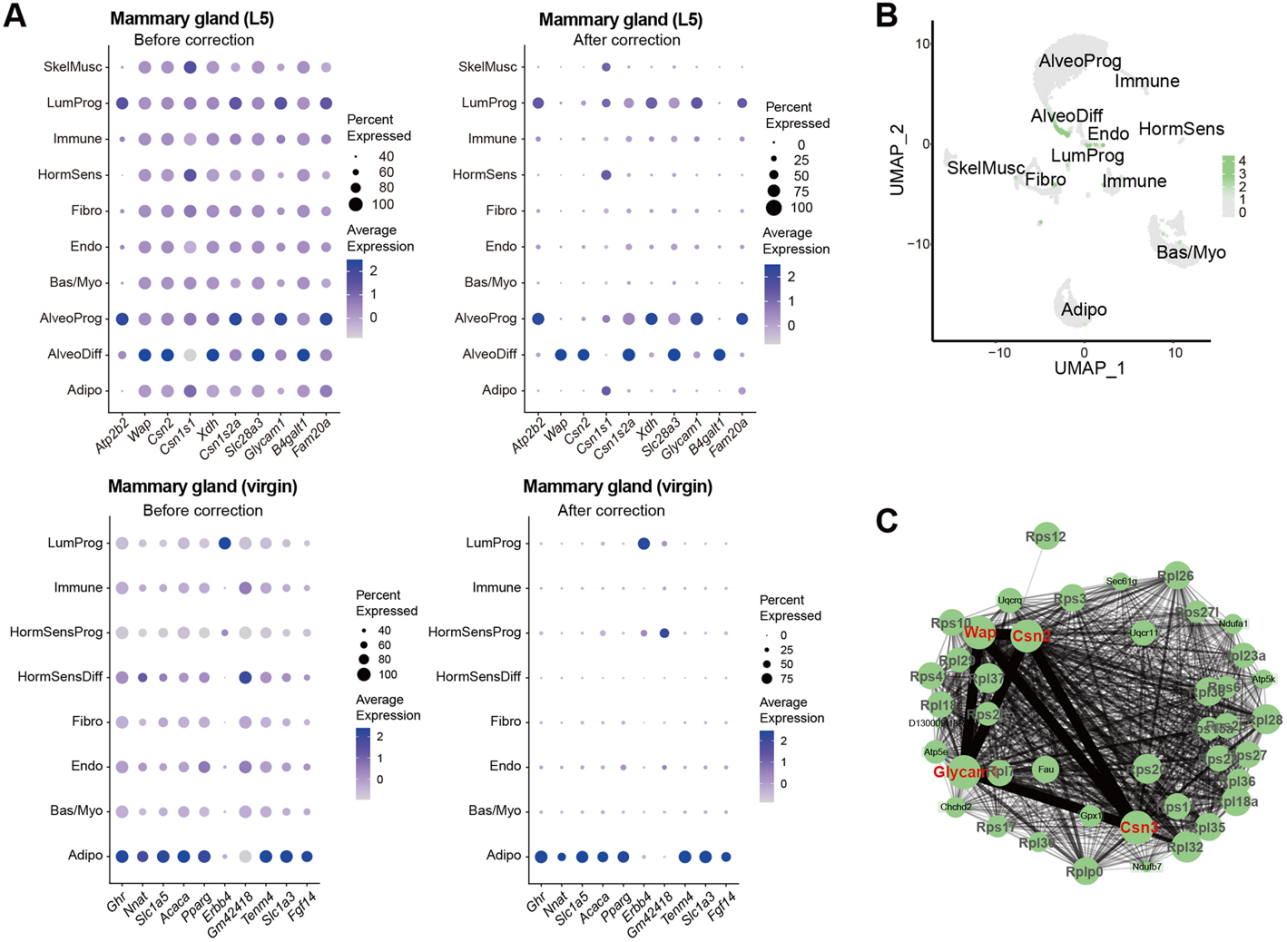
Contamination detection and correction of scCDC improves cell-type marker gene profiling and network construction in the snRNA-seq datasets of mammary glands. **A** Dotplots show the expression of selected GCGs in L5 mammary gland and virgin mammary gland before (left) and after correction (right), respectively. **B**, **C** The significant gene network module associated with GCGs after correction is identified in L5 mammary gland after application of scCDC. **B** The mean expression of identified AlveoDiff specific module genes by scWGCNA analysis after scCDC correction. **C** The AlveoDiff specific gene network in (**B**) is presented. GCGs are highlighted in red; ribosomal genes are highlighted in gray

Eventually, we examined if downstream analysis could benefit from the correction by scCDC in our datasets. Owing to its robustness, correction by scCDC unmasked the expression patterns of some GCGs as cell-type marker genes, thus facilitating the identification of cell types. For instance, in the lactating mammary gland (L5) snRNA-seq dataset we produced, scCDC revealed the unique expression of milk protein-coding genes, such as *Wap* and *Csn2*, in AlveoDiff (differentiated alveolar) cells (Fig. 7A). In the virgin mammary gland snRNA-seq dataset, scCDC showed the exclusive expression of adipocyte markers *Ghr*, *Acaca*, *Slc1a5*, *Nnat*, and luminal progenitor marker *Erbb4* (Fig. 7A).

Cell-type-specific gene network is informative in scRNA-seq analysis, which demands accurate cell-type-specific gene profiling. Therefore, we examined if corrected cell marker profiling by scCDC also improves gene network construction. We applied single-cell weighted gene co-expression network analysis (scWGCNA) [37] to the lactating mammary gland snRNA-seq dataset before and after scCDC’s correction (Additional file 6: Table S5). Although the GCGs *Csn2*, *Csn3*, *Wap*, and *Glycam1* are well-known lactation-specific genes regulated by the same transcriptional machinery [38], they were not identified in any network modules before scCDC’s correction. In contrast, these four lactation-specific genes were identified in a network module of AlveoDiff cells after scCDC’s correction (Fig. 7B,C and Additional file 6: Table S5). Notably, a dozen of ribosomal protein-coding genes were also enriched in the module, in line with the biological fact that translation machinery is extensively active to produce a large amount of milk proteins in AlveoDiff cells at lactation [39, 40]. The results indicate that scCDC’s decontamination helped scWGCNA identify gene co-expression modules masked by ambient RNA contamination.

We also examined if the correction by scCDC facilitated marker gene profiling and gene network construction in the pancreas dataset. Similar to the observations in our in-house mammary gland datasets, scCDC unmasked the expression patterns of cell-type marker genes. For example, scCDC revealed the exclusive expression of *Ins1* and *Ins2* in Beta cells and *Gcg* in Alpha cells (Additional file 1: Fig. S16A). Additionally, in the pancreas scRNA-seq dataset, many cell-type marker genes found as GCGs (such as Alpha-cell marker *Gcg* and Delta-cell marker *Sst*) were not identified in network modules before scCDC’s correction. Only after scCDC’s correction, *Sst* and *Gcg* were identified as central genes of Delta cells’ and Alpha cells’ network modules, respectively. Noticeably, *Irx1* and *Irx2*, the two genes incorrectly wiped out by scAR, were also identified in the Alpha cell module as expected (Additional file 1: Fig. S16B and Additional file 6: Table S5). Taken together, scCDC significantly improved cell-type-specific marker profiling and downstream co-expression network construction.

## Discussion

Here, we developed a computational method, scCDC, to identify GCGs and correct the counts of GCGs, without requiring experimental spike-in controls or empty droplets. Our results indicate that ambient RNA contamination warrants attention, and scCDC effectively identified GCGs and corrected their contamination in scRNA-seq and snRNA-seq data. Compared to the existing computational methods, scCDC avoids the under-correction issue of DecontX, CellBender, and SoupX-automated on highly contaminating genes and the over-correction on other genes by SoupX-manual and scAR, via the detection of GCGs (Table 1), ensuring robust correction for varying levels of contamination.

Among the existing computational methods, SoupX, CellBender, and scAR estimated the contaminative count distribution from empty droplets. However, these three methods have two limitations. First, it is too simplistic to assume that ambient RNA levels have the same distribution in empty droplets and in cell- or nucleus-containing droplets. The two reasons are (1) empty droplets only contain ambient RNAs randomly distributed in the cell suspension, but cell- or nucleus-containing droplets may also contain ambient RNAs specifically attached to or absorbed by cells or nucleus; (2) unlike cell- or nucleus-containing droplets, in empty droplets, the lack of endogenous RNAs may lead to more amplification of ambient RNAs and thus over-estimation of the contamination, e.g., the over-correction by SoupX and scAR on the scRNA-seq datasets. Second, these three methods are inapplicable to the processed gene-by-cell count matrices, which are common in public datasets and do not contain empty-droplet data.

In contrast, scCDC avoids these limitations by estimating the distribution of contaminated counts from real cells or nuclei, so scCDC can be applied to processed count matrices. Although DecontX can also be applied to processed count matrices, the correction efficacy of DecontX is low on highly contaminating genes. We speculate that the DecontX algorithm’s convergence and iteration setting require further optimization. However, scCDC also has its own limitation in that it may not be capable of identifying certain lowly contaminating genes as GCGs and, therefore, does not offer correction for these genes. For datasets with both highly and lowly contaminating genes, we recommend a combined use of scCDC and DecontX to harness the complementary advantages of both methods to achieve an effective correction for all genes.

What mainly distinguishes scCDC from the existing methods is that scCDC detects GCGs and only corrects the expression counts of GCGs. This gene-specific strategy, which was also used in scImpute for the imputation problem, minimizes data alteration to avoid the over-correction issue of SoupX and scAR [41]. In correcting the counts of GCGs, scCDC, SoupX-manual, and scAR are all effective methods, correcting the median expression of GCGs in eGCG − cells to around zero in most datasets (Fig. 4A). Nevertheless, none of the methods could clear all the counts of GCGs in eGCG − cells, leaving certain contaminative counts of GCGs in a small population of eGCG − cells (Additional file 3: Table S2), which may slightly affect cell clustering and other analyses. Of note, we were able to design scCDC to clear the counts of GCGs in eGCG − cells aggressively. However, this strategy will alter the natural count distribution of GCGs in the entire dataset and may hinder the combined use of scCDC with other methods.

It is noted that scCDC and DecontX require the pre-clustering of cells, an issue we discussed in the Method Appendix (Additional file 1). Notably, identifying known cell types is not significantly affected by ambient RNA contamination, at least in the datasets we have tested. This is verified by examination of cluster ARI before and after iterative correction by scCDC (Additional file 5: Table S4). And a number of cell-type annotation tools (Azimuth [42], SingleR [43], Cell Blast [44], SciBet [45]) and databases (CellMarker [46], PanglaoDB [47]) have been developed to help define cell types in a supervised way. For example, the NIH HuBMAP consortium has released Azimuth, which provides reference cell types for many human tissues (https://azimuth.hubmapconsortium.org). Moreover, novel tools like scDesign3 can be used to justify the preclustering accuracy. In contrast to DecontX and scCDC, SoupX, CellBender, and scAR do not require cell pre-clustering (Table 1). However, we noticed that manually pre-defining contaminating genes after preclustering strikingly improved the correction accuracy of SoupX in all datasets we tested. In the automated setting, SoupX failed to provide sufficient correction, consistent with the result in a recent report [48]. These results again suggest the necessity of cell pre-clustering before contamination correction.

Similar to scRNA-seq and snRNA-seq data, single-cell proteomics data were also found to have contamination [11]. Accordingly, decontamination methods such as dbs were developed [49]. Although we focused on correcting the contamination in scRNA-seq and snRNA-seq data in this study, scCDC is also applicable to single-cell proteomics data in theory. The performance of scCDC on single-cell proteomics data can be benchmarked in a future study.

## Conclusions

Contamination by ambient RNAs is ubiquitous in single-cell and single-nuclei RNA-seq assays. We proposed scCDC as a novel computational method to detect global contamination-causing genes and correct these genes’ expression data. The gene-specific correction strategy enables scCDC to correct highly contaminating genes and be less likely to over-correct lowly/non-contaminating genes, compared to the existing computational methods. Decontamination by scCDC improves marker gene identification and gene network construction.

## Methods

### Calculation of cell-cluster-specific gene entropy divergences in scCDC

For gene $g$ in cell cluster $c$, the *entropy* is defined as

$$E_{g,c}=-\sum_{n\in V}p_{n,g,c}{\text{log}}_2(p_{n,g,c}),$$

where $V$ is the set of unique values in ${v}_{g,c}$, a vector of gene $g$’s counts in the cells in cluster $c$; ${p}_{n,g,c}$ is the frequency of the count value $n$ in ${v}_{g,c}$, defined as

$$p_{n,g,c}=\frac{\#\text{ of occurrences of value }n\text{in }v_{g,c}}{\text{length of }v_{g,c}}.$$

For cell cluster $c$, the following procedure is used to calculate the cell-cluster-specific *expected entropy-expression curve*, inspired by the ROGUE score in [50].

1. Calculate each gene g’s mean expression in cell cluster c as

$$M_{g,c}=\text{log}\left(\text{(average of}v_{g,c})+1\right)\text{,}$$

And calculate ${E}_{g,c}$ defined above.

2. For $b=1, \dots , 10$, in the $b$-th subsampling run, do the following.
  i. Randomly sample 80% of genes.
  ii. Use the R function smooth.spline() to fit a curve between the sampled genes’ entropy values (*y*; response variable) and mean expression values (*x*; explanatory variable), using the following R code:
    $$\text{smooth}.\text{spline}(\text{M}_\text{c}^\text{b},\text{E}_\text{c}^\text{b},\text{ spar}\hspace{0.17em}=\hspace{0.17em}1),$$
    where ${M}_{c}^{b}$ is a vector containing the randomly sampled genes’ ${M}_{g,c}$ values, and ${E}_{c}^{b}$ is a vector containing the randomly sampled genes’ ${E}_{g,c}$ values. Denote the fitted curve by function ${\widehat{f}}^{b}$ that maps a gene’s mean expression to entropy.
  iii. For each sampled gene $g$, calculate the residual ${r}_{g,c}^{b}={\widehat{f}}^{b}\left({M}_{g,c}\right)-{E}_{g,c}$, i.e., the difference between the gene’s fitted entropy from step ii and the actual entropy. Pool all residuals into a vector ${r}_{c}^{b}$. Assuming all residuals follow a normal distribution, define gene $g$ as an outlier if its residual falls into the top 1% tail of the fitted normal distribution, i.e., using R code, if
    $$1-\text{ pnorm}(\text{r}\_(\text{g},\text{c})^\text{b},\text{ mean}\hspace{0.17em}=\hspace{0.17em}\text{mean}(\text{r}\_\text{c}^\text{b}),\text{ sd}\hspace{0.17em}=\hspace{0.17em}\text{sd}(\text{r}\_\text{c}^\text{b})) \le 0.01$$
  iv. Remove the outlier genes detected in step iii and refit the curve as in step ii.
  v. Detect outlier genes as in step iii based on the refitted curve in step iv.
  vi. Remove the outlier genes detected in step v and refit the curve as in step ii.
  vii. Output the curve from step vi.

3. Calculate the expected entropy-expression curve by averaging the 10 curves from the subsampling runs. Specifically, for each gene $g$ , its expected entropy is the average of the 10 fitted entropy values.

Finally, the *entropy divergence* of $g$ in cell cluster $c$ is defined as

$${\Delta E}_{g,c}={\widehat E}_{g,c}-E_{g,c^,}$$

where ${\widehat{E}}_{g,c}$ is the expected entropy of gene $g$ in cell cluster $c$, calculated based on gene $g$’s average expression and the expected entropy-expression curve in cell cluster $c$. Since we expect that gene $g$’s ambient RNAs would deflate its entropy ${E}_{g,c}$, a large and positive ${\Delta E}_{g,c}$ would indicate severe contamination of gene $g$ in cell cluster $c$.

### GCG identification in scCDC

Small cell clusters (with fewer than 100 cells) are not considered in this GCG identification step. Figure 2B illustrates the GCG identification procedures described below.

1. Among the considered cell clusters, in every cluster $c$, the genes with “significantly” large entropy divergences would be identified as the *candidate GCGs* of cluster $c$. Specifically, we fit a normal distribution of all genes’ entropy divergences ${\Delta E}_{g,c}$’s, denoted by the vector ${\Delta E}_{c}$. Then we calculate a pseudo-*p*-value of gene $g$, denoted by $p{p}_{g}$, as

$p{p}_{g}=1-$ pnorm(${\Delta E}_{g,c}$, mean = mean(${\Delta E}_{c}$), sd = sd(${\Delta E}_{c}$)), and set a 0.05 threshold on the adjusted pseudo-*p*-values based on the Benjamini–Hochberg procedure. That is, any gene $g$ whose post-adjustment $p{p}_{g}\le 0.05$ would be called a candidate GCG in cluster $c$, if gene $g$ is expressed in at least 80% of the cells in cluster $c$.

2. Across the considered cell clusters, the genes found as candidate GCGs in at least 50% (referred to as the *restriction factor*, which can be user-specified; the selection of an appropriate restriction factor is discussed in the Method Appendix) of the clusters and have non-zero counts in at least 20% cells in each cell cluster would be found as the *GCGs*. In other words, the GCGs are the genes that are stably found as candidate GCGs in many clusters.

### Estimation of a GCG’s contaminative count distribution in scCDC

Each GCG’s contaminative count distribution is estimated by the GCG’s counts in the cells that are not expected to express the GCG endogenously (i.e., *eGCG − cells*; illustrated in Fig. 2B). To identify a GCG’s eGCG − cells, we take the following procedure. First, we filtered out cell clusters of less than 50 cells and identified the cluster in which the GCG has the lowest mean expression, calling this cluster an eGCG − cluster. Second, we use the GCG’s expression level as the only feature in a binary classification setting: distinguishing the eGCG − cluster from another cluster, so we can compute the area under the ROC curve (AUROC) to indicate the similarity of the other cluster to the eGCG − cluster (the larger the AUROC, the higher the similarity); the AUROC computation is done using the “pROC” (v1.17.0.1) package [51]. All clusters with AUROC values of less than 0.9 (a tuning parameter; see Table 2) are pooled with the eGCG − cluster and defined as the *eGCG − cells*. A GCG’s *eGCG* + *cells* are defined as the remaining cells, which are expected to have the GCG endogenously expressed.

**Table 2:** Tuning parameters in the functions of scCDC

Function	Parameter	Description	Default
Contamination detection	restrict_factor	The minimum proportion of cell clusters in which a GCG is found as a candidate GCG	0.5
	min.cells	The minimum cell number of the cell clusters used for finding candidate GCGs	100
Contamination correction	auc_thres	The threshold of the AUROC used to define eGCG − clusters	0.9
	min.cell	The threshold to filter the cell populations with an insufficient number of cells	50

### Contamination ratio of a GCG by scCDC

The contamination ratio $\left(C\right)$ of gene $g$ is calculated by the total UMI count of gene $g$ in eGCG − cells divided by the total UMI count of all genes in eGCG − cells:

$${R}_{g}= \frac{\sum_{j\in {N}_{g}}{X}_{g,j}}{\sum_{g{\prime}\in G}\sum_{j\in {N}_{g}}{X}_{g{\prime},j}}$$

where ${X}_{g,j}$ represents the observed UMI count of gene $g$ in cell $j$, ${N}_{g}$ represents the eGCG − cells of gene $g$, and $G$ represents all genes in the data.

### Correction of a GCG’s contaminative counts by scCDC

Given a GCG, scCDC uses the Youden index-based method to find a threshold $c$ so that the GCG’s contaminative counts in all cells would be corrected by subtracting $c$. To find $c$, we generate the ROC curve for classifying the eGCG − cells (class 0) and the least eGCG + cells (the eGCG + cell cluster in which the GCG has the lowest AUROC value against the GCG − cluster with the lowest expression of GCG). Based on the ROC curve, we calculate the Youden Index $(J)$ [52] of a given threshold $c$:

$${J}_{c}={Se}_{c}+ {Sp}_{c}-1,$$

where ${Se}_{c}$ is the sensitivity at the threshold $c$, and ${Sp}_{c}$ is the specificity at the threshold $c$. Then we find the threshold $c$ by maximizing ${J}_{c}$. Given the threshold $c$, we correct the GCG’s count in every cell by subtracting $c$, with a truncation at zero so that the GCG would not have negative counts.

In summary, scCDC has four tuning parameters listed in Table 2.

### Count correction by SoupX, DecontX, CellBender and scAR

SoupX (v1.5.2), DecontX in Celda (v1.10.0), CellBender (v0.3.0), and scAR (v0.4.3) were employed for count correction.

For SoupX, both the raw feature matrix and filtered feature matrix generated by Cellranger (v6.0.1) are used to create the Soup Channel object, followed by the standard correction workflow in the tutorial [5]. The “automated” and “manual” modes are applied, respectively. The identified GCGs are provided as the “non-expressed genes,” whose RNAs in specified cells are treated as ambient, in the “manual” mode.

For DecontX, the correction is applied to the filtered feature matrix. The default procedure, referred to as the “default” mode, is performed. Alternatively, the pre-clustering information obtained from Seurat [53] is provided manually in the “pre-clustered” mode.

For CellBender, the correction uses the raw feature matrix with the remove-background function, following the tutorial (https://cellbender.readthedocs.io/en/latest/getting_started/remove_background/index.html).

For scAR, both the raw feature matrix and filtered feature matrix are used based on the tutorial (https://scar-tutorials.readthedocs.io/en/latest/tutorials/scAR_tutorial_mRNA_denoising.html). The filtering scale is applied to the filtered feature matrix for each of the datasets, as listed in Additional file 7: Table S6.

### Generation of simulated single-cell datasets

To generate the simulated PBMC single-cell dataset, we first obtained a real PBMC dataset “pbmcsca.SeuratData” from the SeuratData R package (https://github.com/satijalab/seurat-data). We then sub-selected the dataset generated by the 10 × Chromium (v2) technology under the experiment “pbmc2,” using the “meta. data” information from “pbmcsca.SeuratData.” Next, we filtered out the ERCC spike-in’s, the mitochondrial genes, and the gene *MALAT1*, and we select five cell types (B cells, CD14 + monocytes, natural killer cells, CD4 + T cells, and cytotoxic T cells). Using the filtered and sub-selected real dataset from above, we applied the simulator scDesign2 [34, 54] to fit one multivariate probabilistic model to each of the five cell types.

The resulting gene expression matrix is stored as the file sce_10x_pbmc2_hca_corrected.rds.

The sce_10x_pbmc2_hca_corrected.rds file and the code for reproducing it are available at https://zenodo.org/record/6395574#.YrXp5JPMKEt. In particular, the rds file is under Code summary.zip/Fig. 7 and supplementary S3 to S10/imputation_comparison_0614/Data_gen/data/; the code is under Code summary.zip/Data simulation/. The rds file can be generated by sequentially executing the seven steps in the code directory.

To generate the simulated pancreas data with discrete cell types, we first obtained a real pancreas dataset based on the procedures in the “General single-cell and single-nuclei data processing” section. We selected four major cell types (Alpha, Beta, Gamma, and Delta cells) and sequentially filtered out non-Beta cells whose both *Ins1* and *Ins2*’s count expression is below 1, non-Alpha cells whose *Gcg*’s count expression is below 1, and non-Delta cells whose *Sst*’s count expression is below 1 to obtain a dataset with unambiguous cell clusters. Using this filtered real dataset, we then applied the scDesign2 [34, 54] to fit one multivariate probabilistic model to each of the four cell types.

To generate the simulated pancreas data that follow a continuous trajectory, we first obtained a real pancreas dataset from scDesign3’s [38] Zenodo repository (https://zenodo.org/record/7750930). The data file is PANCREAS_sce.rds, a preprocessed and filtered dataset of pancreatic endocrinogenesis from scVelo [55]. It contains the top 1000 highly variable genes and four cell types that form a single trajectory, as well as the Slingshot [56] inferred cell pseudotime values, which are further normalized into the interval [0, 1]. We then applied scDesign3 [35] to fit one multivariate probabilistic model for all the cells using the pseudotime values as the cell covariates. Finally, we generated the simulated dataset using the scDesign3 fitted model with cell pseudotime values uniformly distributed in [0, 1].

### Artificial contamination of simulated single-cell datasets

To simulate a contaminated PBMC dataset, we blended the simulated uncontaminated count matrix with artificial contaminative counts of three marker genes of CD14 + monocytes, *S100A9*, *S100A8*, and *LYZ*. In Additional file 1: Fig. S4A, the artificial contaminative counts of each gene were generated following a NB distribution, whose mean was the gene’s average original count and whose size was 10. Alternatively in Additional file 1: Fig. S4A, B, the three genes’ artificial contaminative counts were generated from a NB distribution with a fixed mean (0.5, 1, 1.5, 2, 2.5, or 3, indicating the contamination level) and a size of 1, 10, 50, or 100. Then for each of the three genes, we added to each of its original counts an artificial contaminative count, which was randomly picked from the generated ones.

We also generated a contaminated pancreas dataset by generating artificial contaminative counts of top 500 contaminating genes, which had the largest total counts in the empty droplets of the real pancreas dataset [6]. Specifically, we used the average count of *Ins2* in non-Beta cells, in which *Ins2* should not be expressed, as the baseline contamination level. Then for each of the 500 contaminating genes, we calculate its contamination level by multiplying the baseline contaminative level with the ratio of (the gene’s total count in the empty droplets)/(*Ins2*’s total count in the empty droplets). Finally, corresponding to the low, medium, or high contamination, each contaminating gene’s contaminative counts were sampled from a NB distribution, whose mean was 0.3, 1, or 3 times the gene’s contamination level and whose size was 10.

An additional contaminated pancreas trajectory dataset is generated by randomly blending the original raw count matrix with an artificial contaminative count matrix composed of four marker genes of Beta cells, *Ins1*, *Ins2*, *Iapp*, and *Nnat*. The contaminative count matrix is generated following NB distributions using the mean of the average count of the original raw count matrix and a size of 30. Then a contaminative count is randomly selected from the matrix and added to each original raw count.

### Single-nuclei RNA-seq of mammary glands

Eight-week-old female C57BL/6N mice were timed mated. Abdominal and thoracic mammary tissues from nulliparous mice (virgin) and mice at lactation day 5 (L5) were harvested and lymph nodes in abdominal mammary tissues were removed. Mammary tissues were snap-frozen in liquid nitrogen followed by nuclei extraction and single-nuclei RNA sequencing (snRNA-seq) on a 10X Genomics platform in Lianchuan Biology Technology Co.

### General single-cell and single-nuclei data processing

Cellranger (v6.0.1) was used to map raw reads to mouse or human reference genomes and obtain raw and filtered count matrixes of genes. Seurat (v4.0.3) was used for data filtration, principal component analysis (PCA), dimension reduction, clustering, marker gene identification, and data visualization. Specifically, for each dataset, cells with insufficient genes, molecules, and high mitochondria gene percentage were first filtered. The data were then normalized, and top variable genes were identified. Scaling, dimension reduction, and clustering were then performed. The specific parameters for filtering, dimension reduction, and clustering used in each dataset are provided in Additional file 7: Table S6.

When benchmarking for pre-clustering, the top 1000 variable genes identified by SeuratVST [53], scPNMF (v1.0) [57], and Scater (v1.20.1) [58] were used for dimension reduction, respectively.

Visualization of clusters and identification of marker genes was done in Seurat. Weighted gene co-expression network analysis (WGCNA) was done using the scWGCNA (v1.0.0) package [37].

### Supplementary Information

Supplementary Material 1. Supplementary figures and Methods Appendix. This file contains the supplemental figures in the manuscript. In addition, a Methods Appendix is provided to assist users to understand the default setting of scCDC.

Supplementary Material 2: Supplementary Table 1. Summary of analyzed scRNA-seq and snRNA-seq datasets in this study.

Supplementary Material 3: Supplementary Table 2. Summary of scCDC correction results in the analyzed datasets.

Supplementary Material 4: Supplementary Table 3. Summary of under- and over-correction by different methods in the analyzed sn- and scRNAseq datasets.

Supplementary Material 5: Supplementary Table 4. Identified GCGs and clustering ARI after iterative runs in simulated, mammary gland and PBMC datasets.

Supplementary Material 6: Supplementary Table 5. Gene network modules identified by WGCNA before and after scCDC correction in pancreas and mammary gland datasets.

Supplementary Material 7: Supplementary Table 6. Summary of parameters used in the analysis of different datasets.

Supplementary Material 8. Review history.

## Data Availability

The scCDC R package is available at https://github.com/ZJU-UoE-CCW-LAB/scCDC [59]. The processed datasets and the code scripts used to generate the figures are available on Zenodo (10.5281/zenodo.6905189) [60]. The published single-cell and single-nuclei datasets were downloaded from the GEO, Synapse databases, 10 × Genomics, and Human Cell Atlas with the accession numbers listed in Additional file 2: Table S1 (syn21694522 [61], GSM4878207 [62], CRA007450 [63], GSM5073381 [64], GSE151048 [65], GSM4230078 [66], SRR10751504 [67], GSM2177570 [68], GSM3489185 [69], GSM3587009 [70], GSM5024089 [71], FCAImmP7528289 [72], GSE142653 [73], https://support.10xgenomics.com/single-cell-gene-expression/datasets/2.1.0/pbmc4k [74], https://www.10xgenomics.com/resources/datasets/1-k-1-1-mixture-of-fresh-frozen-human-hek-293-t-and-mouse-nih-3-t-3-cells-v-3-chemistry-3-standard-3-0-0 [75]). The single-nuclei RNA-seq data of mouse mammary glands are deposited in the Genome Sequence Archive at the National Genomics Data Center, China National Center for Bioinformation / Beijing Institute of Genomics, Chinese Academy of Sciences (GSA: CRA007450) [63]. The total RNA-seq data in mouse lactating and virgin mammary glands are from the GEO database with accession numbers: GSE115370 [76, 77] and GSE52016 [78, 79], respectively. The total RNA-seq data from mouse pancreatic islets were downloaded from GEO: GSE148809 [80, 81]. The genes encoding secretory proteins were predicted in SignalP 4.0 [82], and the list of protein-coding genes were obtained from the Refseq database [83].
