# Supplementary material for: scCDC: a computational method for gene-specific contamination detection and correction in single-cell and single-nucleus RNA-seq data
Source: Genome Biol. 2024 May 23;25:136. doi: 10.1186/s13059-024-03284-w (PMC11112958; doi:10.1186/s13059-024-03284-w)
Supplement: Supplementary file 1 — Supplementary Material 1. Supplementary figures and Methods Appendix. This file contains the supplemental figures in the manuscript. In addition, a Methods Appendix is provided to assist users to understand the default setting of scCDC. [file 13059_2024_3284_MOESM1_ESM.docx]

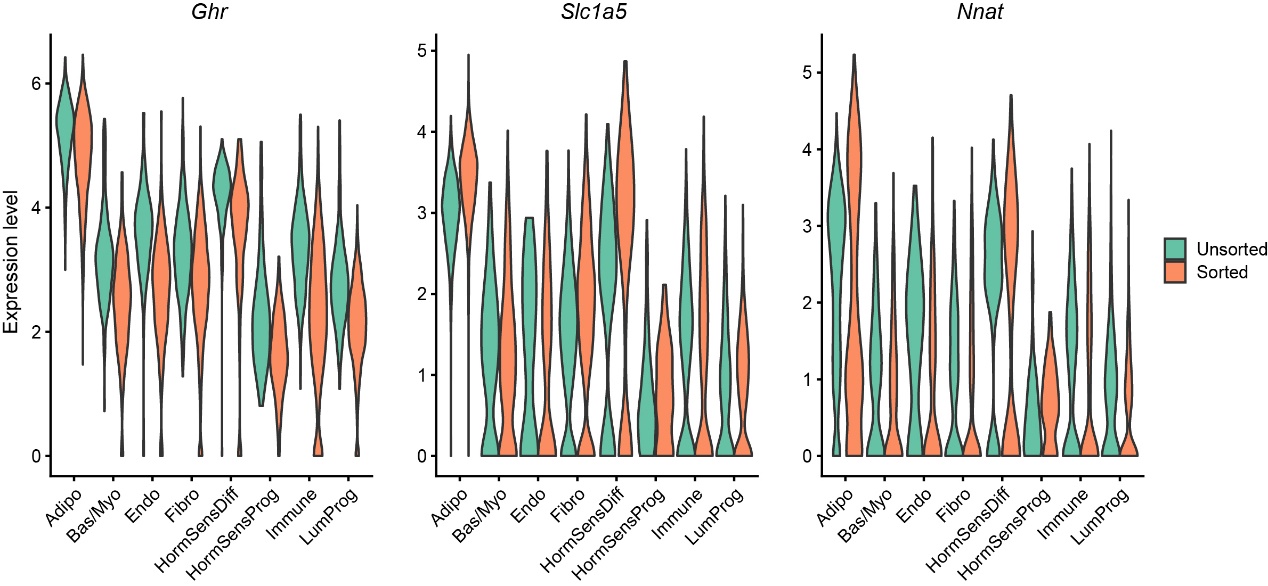


**Supplementary Figure 1.** Flow cytometry cell sorting barely reduced the contamination in the snRNA-seq data of virgin mammary glands. The violin plots show the distributions of the normalized expression levels of three selected marker genes that should be expressed exclusively in one cell type but were detected in many cell types (listed in the horizontal axis).

**
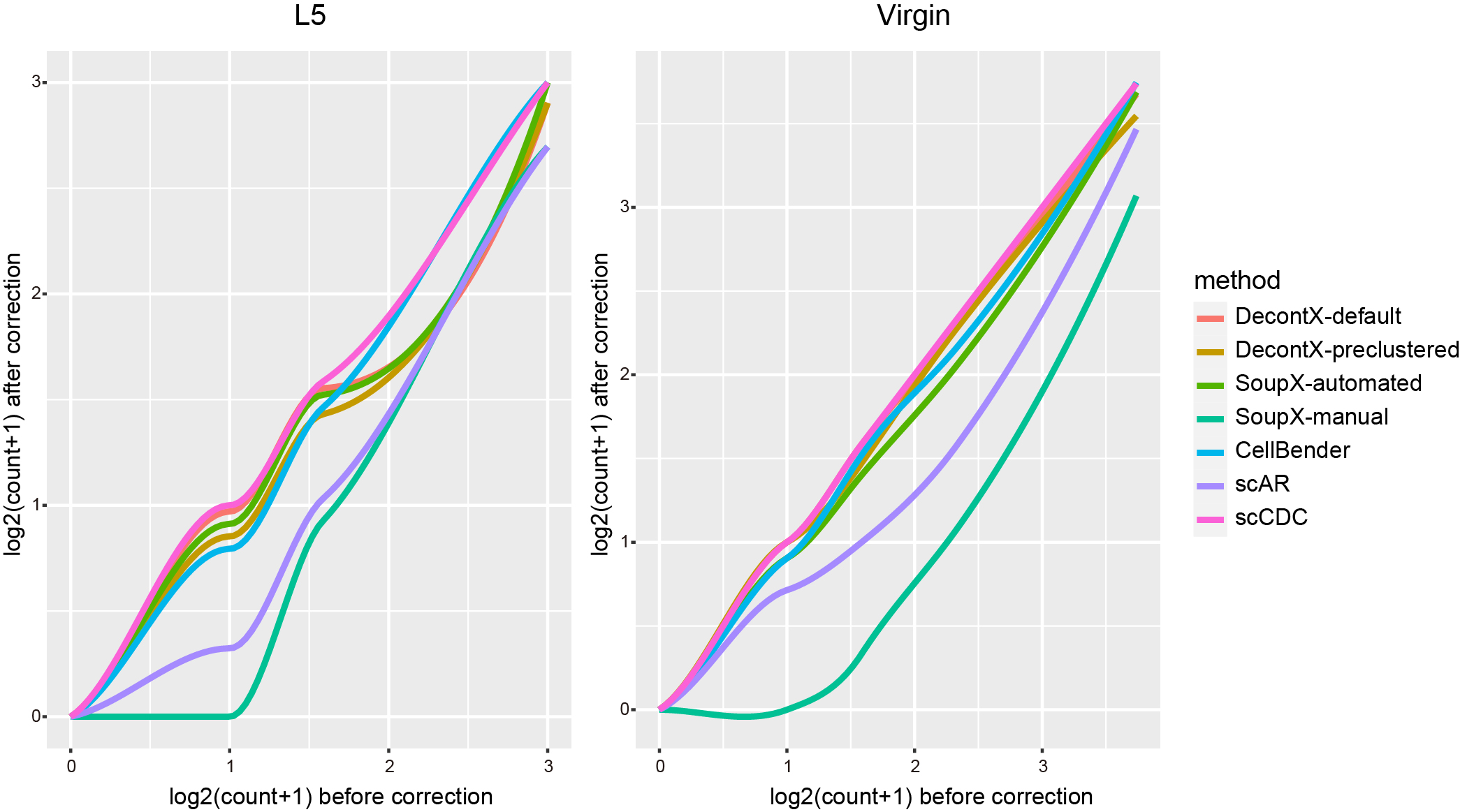
**

**Supplementary Figure 2.** Over-correction by SoupX and scAR in the mammary gland snRNA-seq datasets. For each of 66 well-known housekeeping genes in each dataset, we calculated the 95^th^ percentile of the gene’s expression levels (log2(count+1) values) in all cells before any correction and the 95^th^ percentile of the gene’s corrected expression levels in all cells by each decontamination method. Then we compared the two sets of 95^th^ percentile (each set contains 66 gene’s 95^th^ percentiles) using a Q-Q plot for each decontamination method on each dataset (L5 mammary glands: left; virgin mammary glands: right).


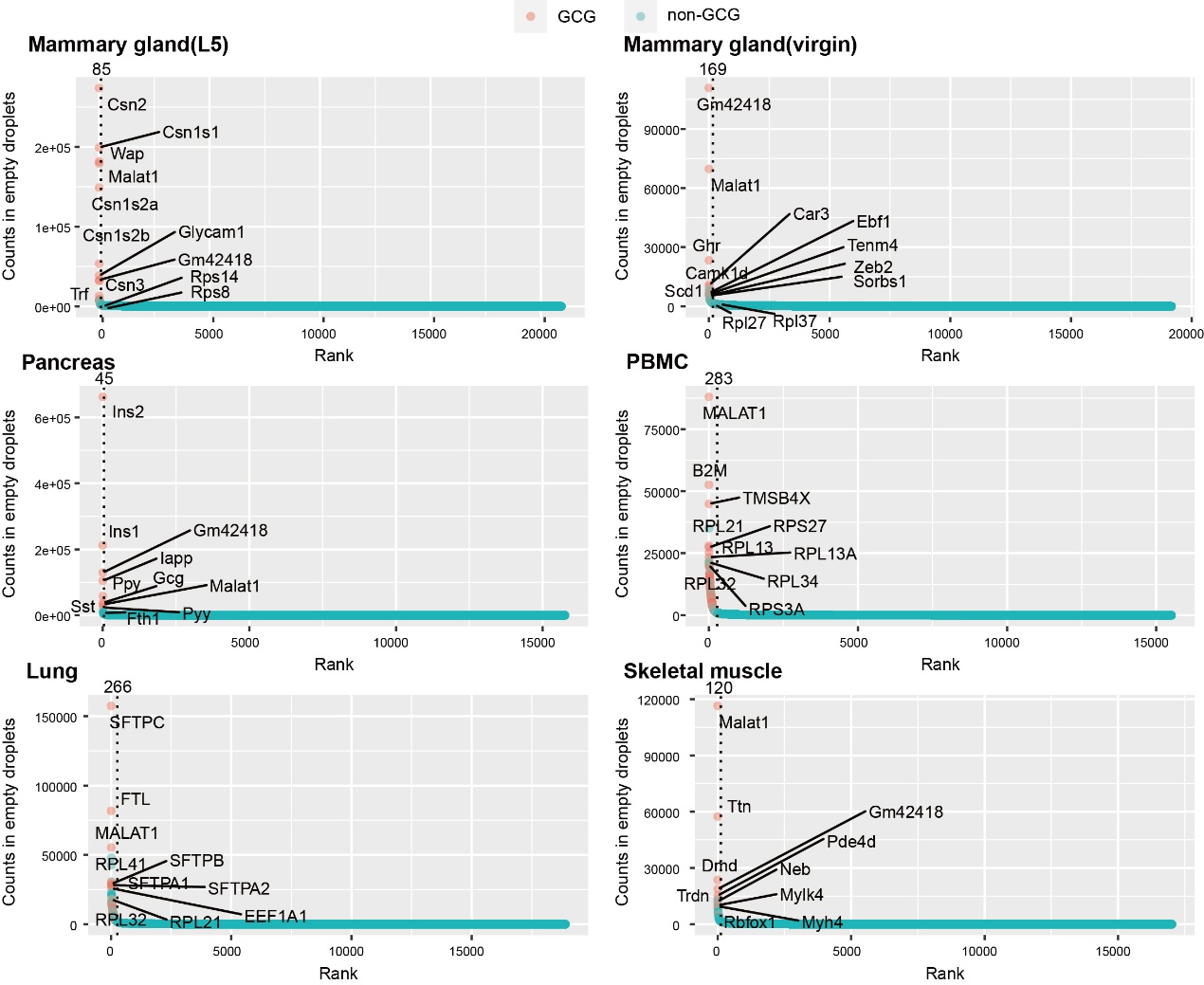


**Supplementary Figure 3.** GCGs identified by scCDC are genes contributing the most abundant ambient RNAs. The plots show the counts vs. rank of genes in empty droplets in the indicated datasets. The dashed lines separate the ‘super-contaminating’ genes and the other genes (details in Method Appendix. See Additional File 1).


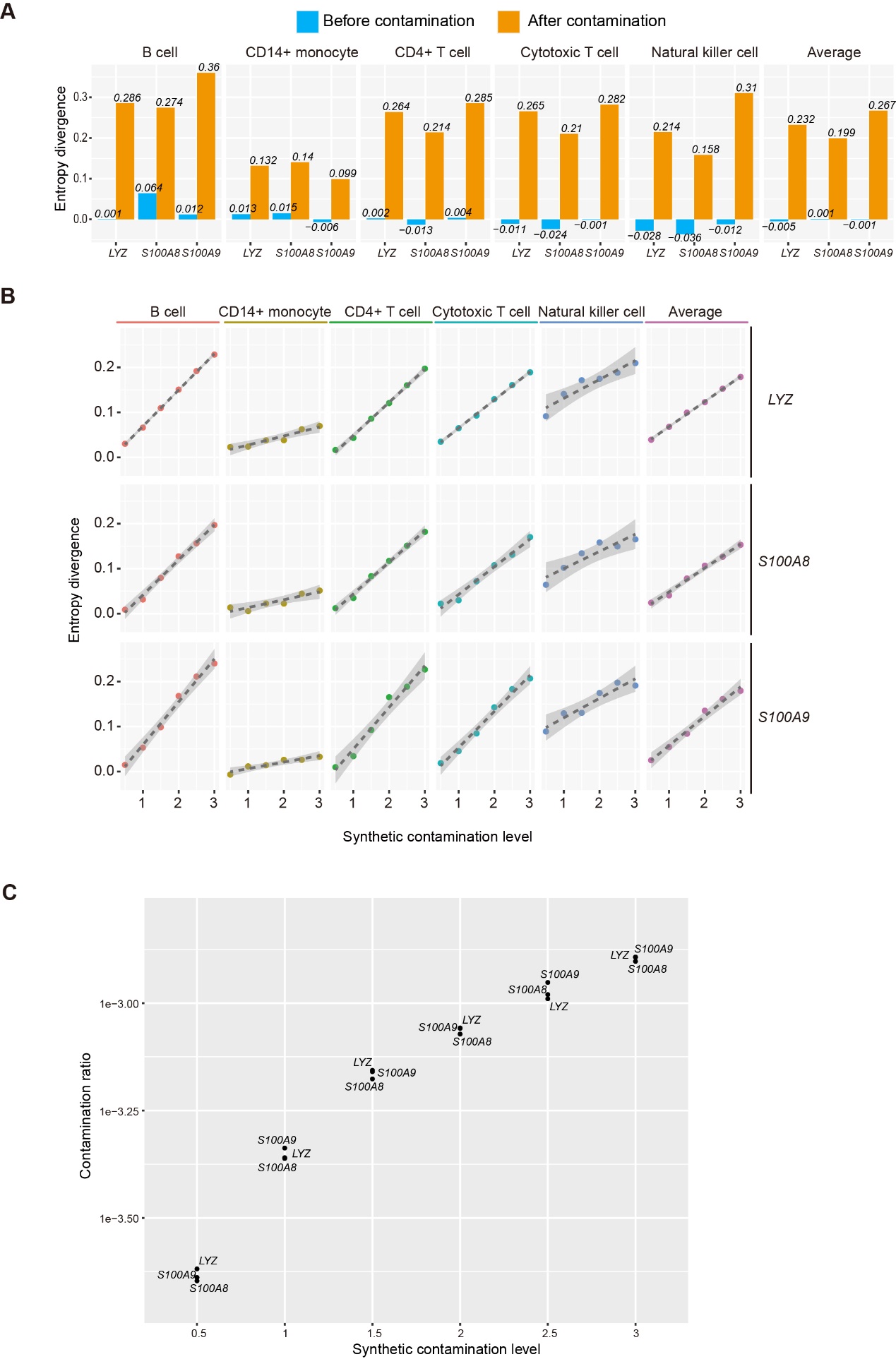


**Supplementary Figure 4.** Simulation confirms the contamination detection functionality of scCDC. (A) Artificial contamination leads to increased Entropy divergency. The bar plots show the entropy divergence from the simulated curves for the three artificially contaminated genes in simulated PBMC data. (B) Entropy divergency positively correlates with synthetic contamination level. The scatter plots show the positive correlation between the entropy divergence and the artificial contamination level. Different contamination level was obtained by adding simulated contaminative counts with indicated mean values (0.5, 1.0, 1.5, 2.0, 2.5, 3.0). For each mean, various distribution size (1, 10, 50, 100) were employed, and the average entropy divergence were calculated and shown. (C) The contamination ratio confirms the synthetic contamination level. The scatter plot shows the correlation between the contamination ratio of the indicated genes and the artificial contamination level as in (B).


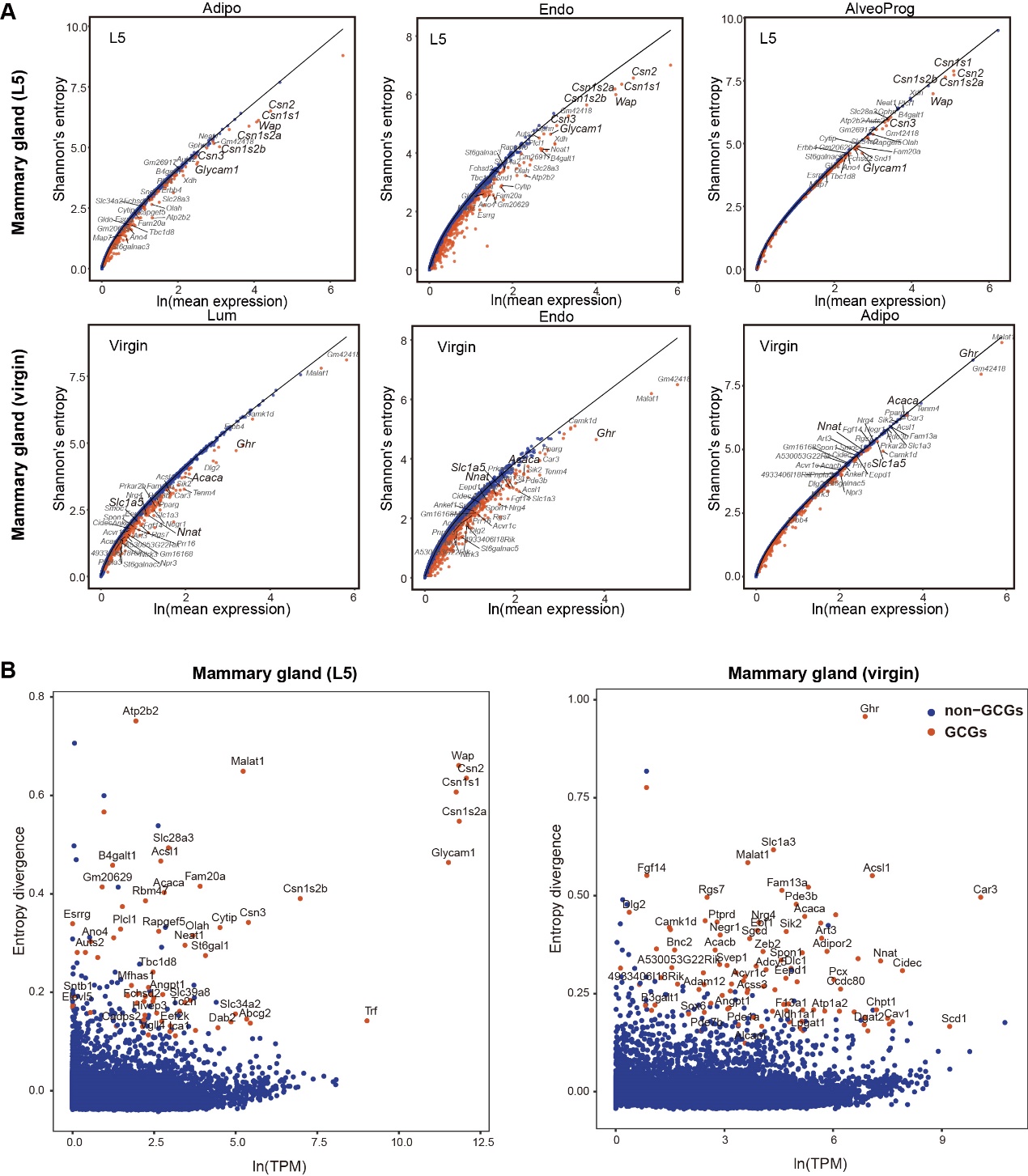


**Supplementary Figure 5.** Contamination detection in the snRNA-seq datasets of mammary gland. (A) The entropy-expression curves of indicated cells. Identified GCGs were labeled on the plot. (B) GCGs are highly-expressed genes in mammary glands revealed by bulk RNA-seq. Scatter plot of the relationship between the mean entropy divergence and the TPM (x-axis, log scale) of all the genes. GCGs are highlighted in red.


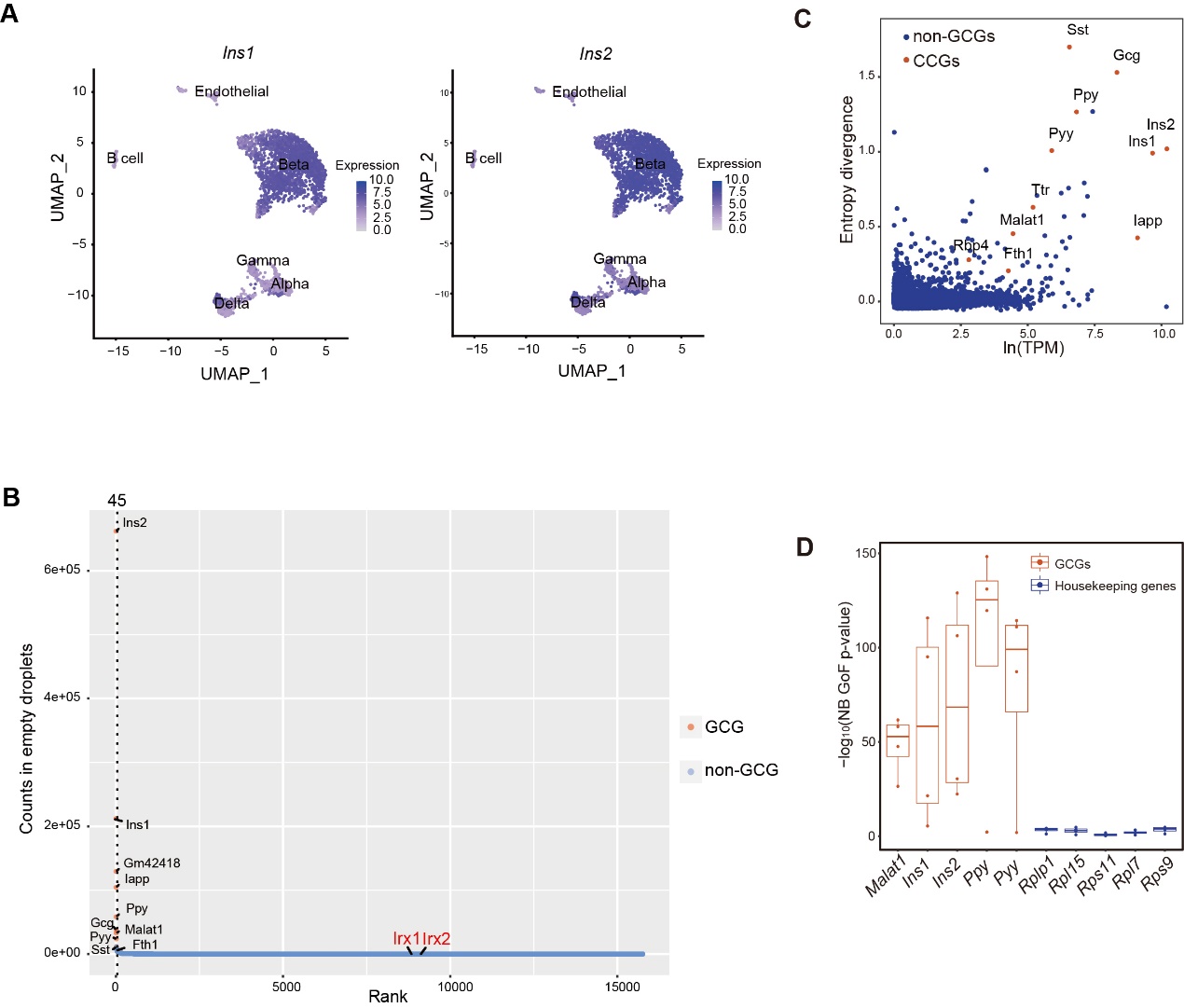


**Supplementary Figure 6.** Contamination detection by scCDC in the single-cell RNA-seq dataset of mouse pancreas. (A) Global contamination is detected in the dataset. The expression of Beta cell markers *Ins1* and *Ins2* in the cells are shown in the UMAP plots. (B) GCGs identified by scCDC are genes contributing the most abundant ambient RNAs. The plot shows the counts vs. rank of genes in the empty droplets. GCGs and *Irx1* and *Irx2* genes are labeled. The dashed line separates the ‘super-contaminating’ genes and the other genes (details in Method Appendix. See Additional File 1). (C) GCGs identified by scCDC are highly-expressed genes revealed by bulk RNA-seq. Scatter plot shows the relationship between the mean entropy divergence and the TPM (x-axis, log scale) of all the genes. GCGs are highlighted in red. (D) Distribution of GCGs is significantly deviated from NB distribution. Box plot of the *p*-values of NB distribution goodness-of-fit test of GCGs and housekeeping genes.


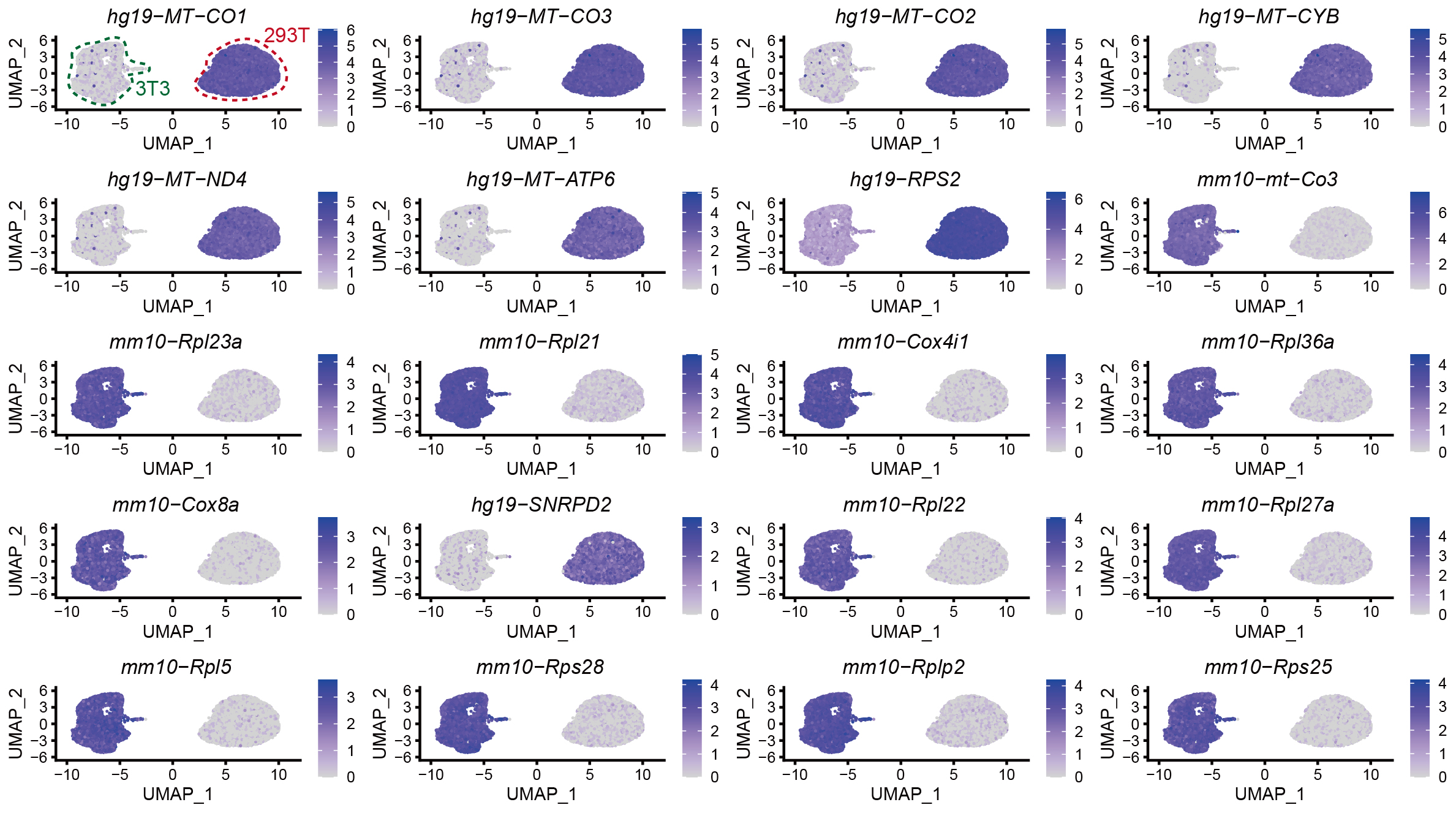


**Supplementary Figure 7.** Contamination of GCGs is low in the ‘barnyard’ dataset of mixed human 293T and mouse 3T3 cells. The expression of selective GCGs by scCDC in the cells are shown in the UMAP plots. The two cell clusters are highlighted.


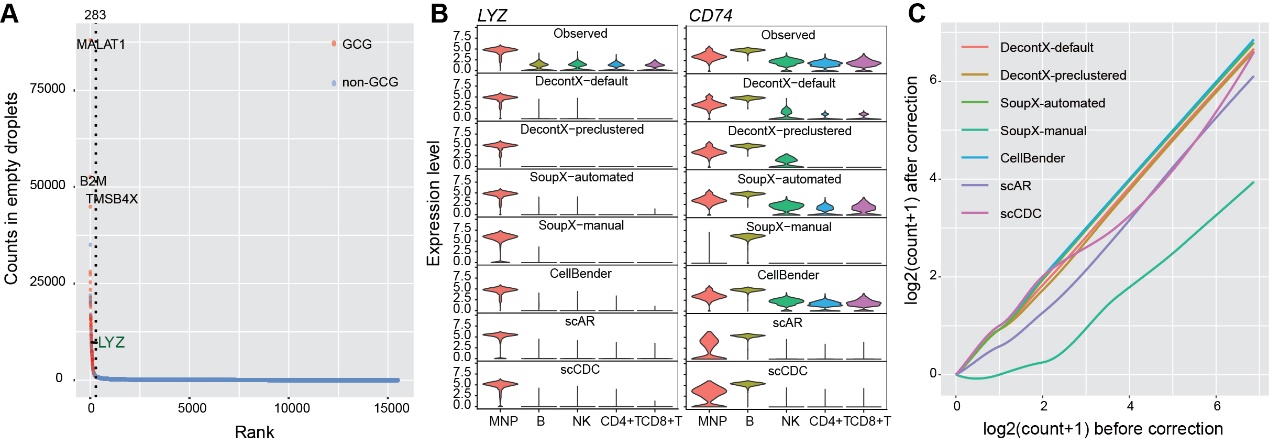


**Supplementary Figure 8.** Comparison of the performance of scCDC, DecontX, SoupX, CellBender, and scAR on the PBMC scRNA-seq dataset. (A) GCGs identified by scCDC are genes contributing the most abundant ambient RNAs. The plot shows the counts vs. rank of genes in the empty droplets. GCGs are labeled. The dashed line separates the ‘super-contaminating’ genes and the other genes (details in Method Appendix. See Additional File 1). (B) The violin plots show the normalized expression levels of *LYZ* and *CD74* before and after correction using indicated methods in the PBMC-4K dataset. (C) Over-correction by SoupX and scAR in the PBMC scRNA-seq dataset. Q-Q plots of the 95^th^ percentile value of counts of 66 housekeeping genes in all the cells after correction by the indicated methods compared with original value of counts.


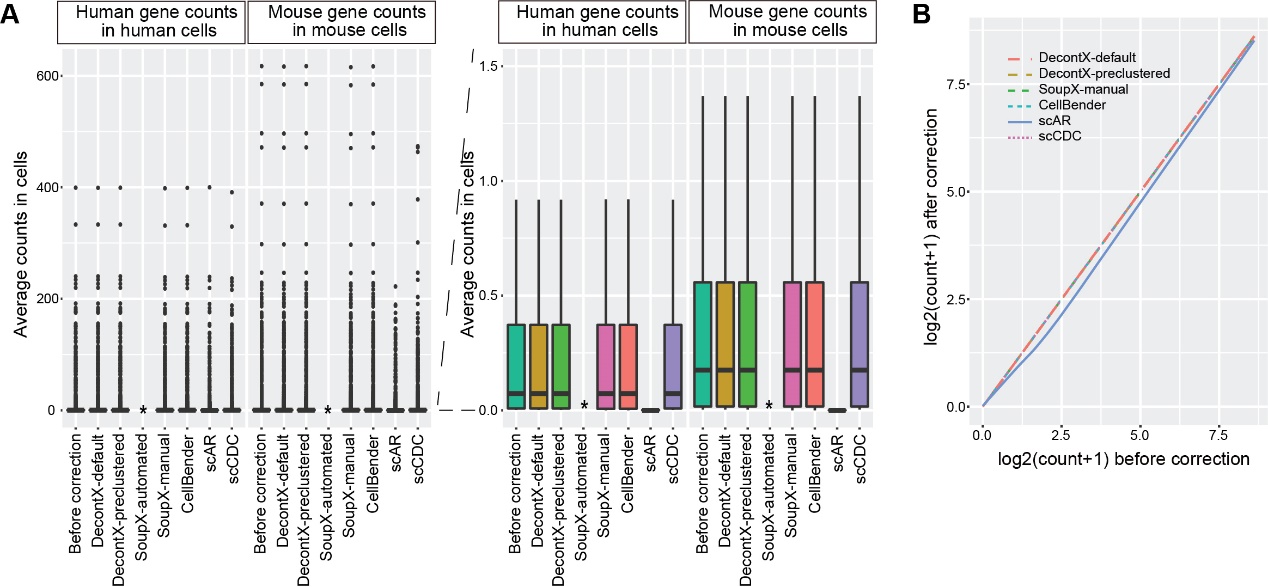


**Supplementary Figure 9.** Over-correction of non-contaminating genes by scAR in the ‘barnyard’ scRNA-seq data of mixed human 293T and mouse 3T3 cells. (A) The box plots show the average counts of endogenous genes in the indicated cells. *, no output. (B) Q-Q plots of the 95th percentile value of counts of 66 housekeeping genes in all the cells after correction by the indicated methods compared with the original value of counts.


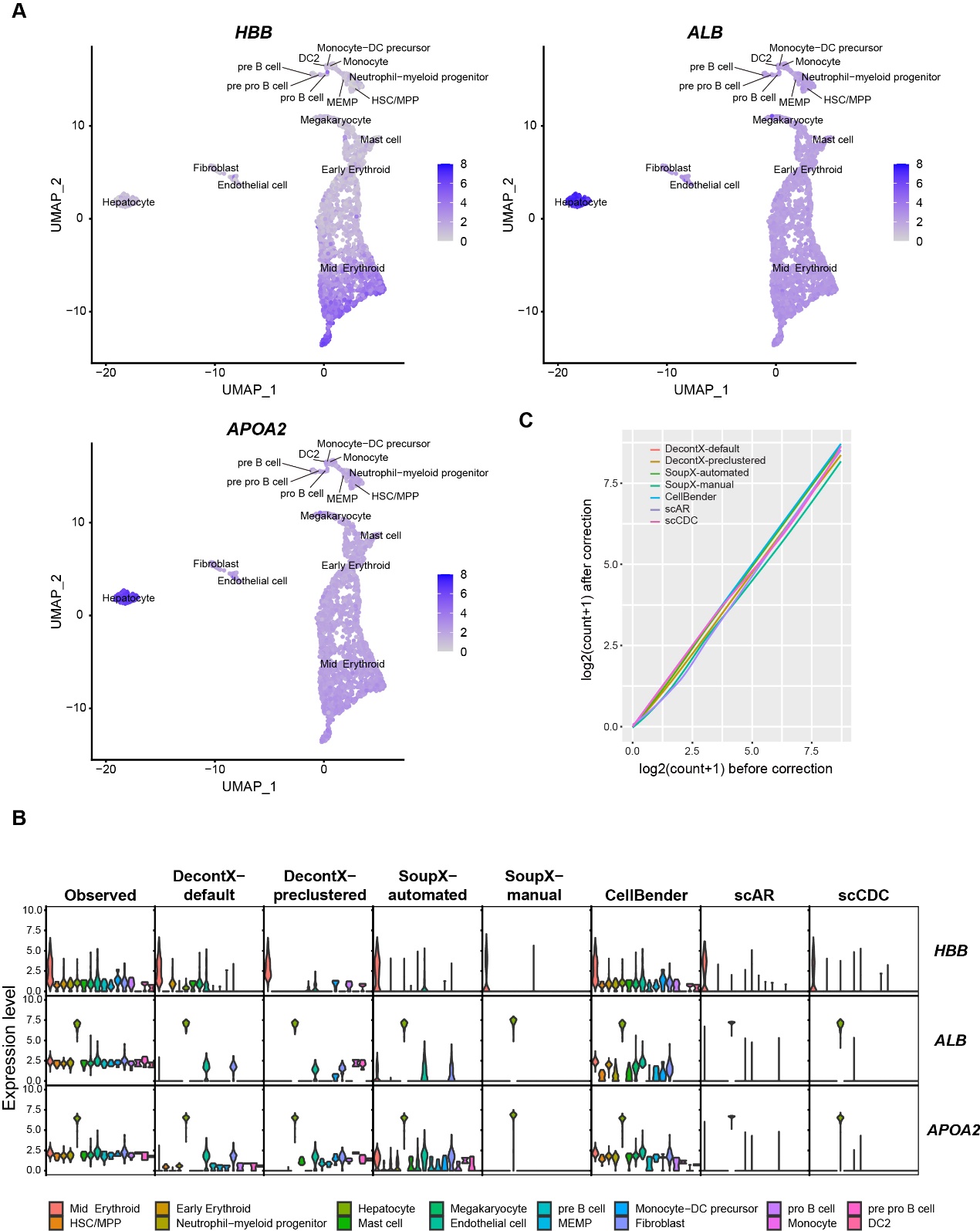


**Supplementary Figure 10.** Comparison of scCDC, DecontX, SoupX, CellBender, and scAR on the scRNA-seq data in the human liver. (A) Global contamination is detected in the dataset. The expression of erythroid marker *HBB* and hepatocyte markers *ALB* and *APOA2* in the cells are shown in the UMAP plots. (B) DecontX and SoupX-manual under-correct the contamination. The violin plots show the normalized expression levels of the indicated GCGs before and after correction using the indicated methods by the default Seurat (V3).(C) scAR moderately over-correct the data. Q-Q plots of the 95th percentile value of counts of 66 housekeeping genes in all the cells after correction by the indicated methods compared with original value of counts.


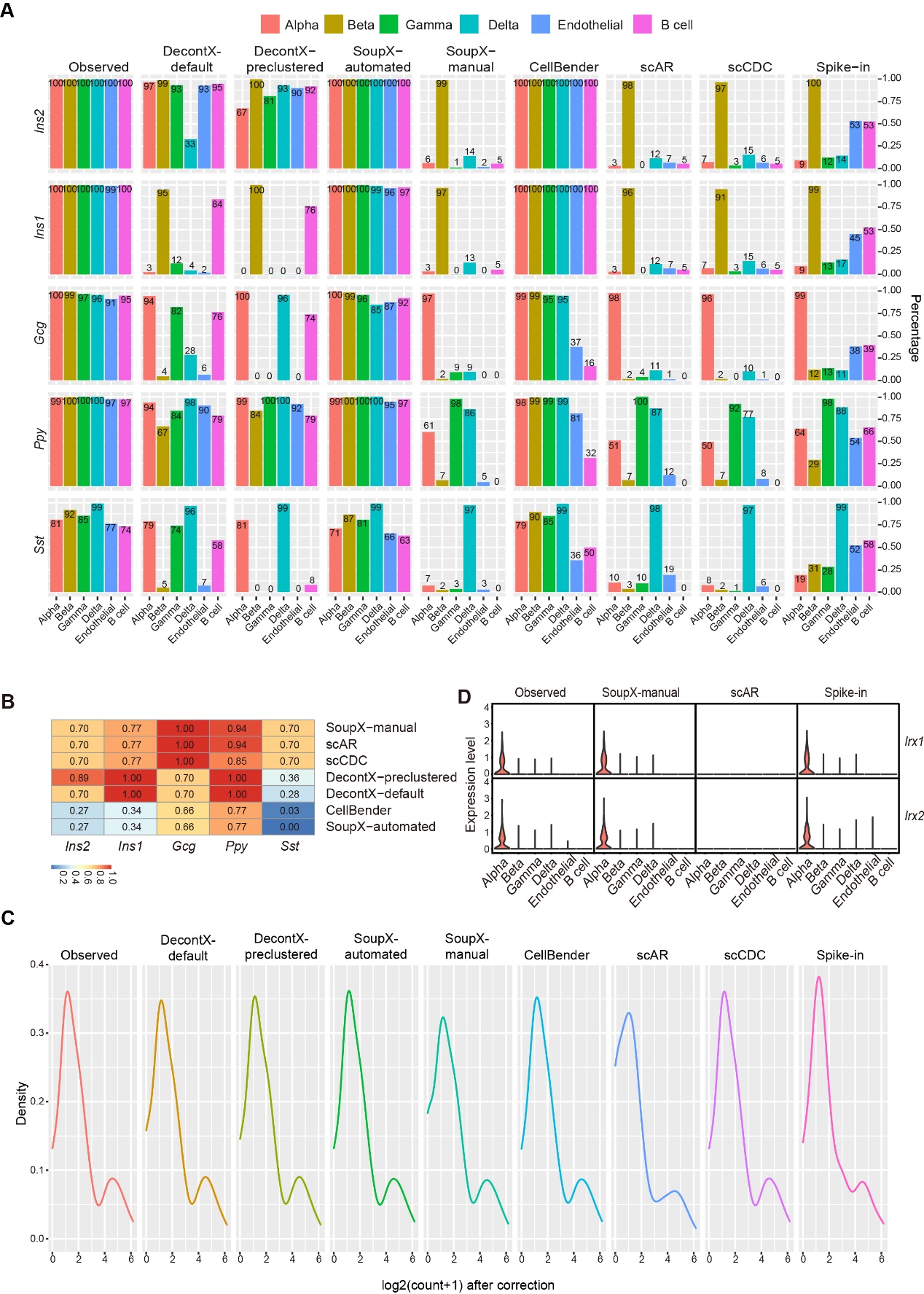


**Supplementary Figure 11.** Comparison of scCDC, DecontX, SoupX, CellBender, and scAR on the scRNA-seq data in mouse pancreas islet with spike-in strategy. (A) DecontX and SoupX-manual under-correct contamination caused by GCGs. The barplots show the percentage of cells expressing GCGs in the cell clusters before and after correction using the indicated methods. (B) The correction results by scCDC, SoupX-manual and scAR highly correlates with the one corrected using spike-in strategy. The heatmap shows *Spearman*’s correlation between the mean expression levels of GCGs in the cell clusters in pancreas data after correction by different computational methods and by spike-in approach. (C-D) Over-correction by SoupX and scAR in pancreas scRNA-seq dataset. (C) The violin plots show the normalized expression levels of the indicated GCGs before and after correction using the indicated methods by the default Seurat (V3). (D) Density plots of the 95^th^ percentile value of counts of 66 housekeeping genes in all the cells before and after correction by the indicated methods in the pancreas scRNA-seq datasets.


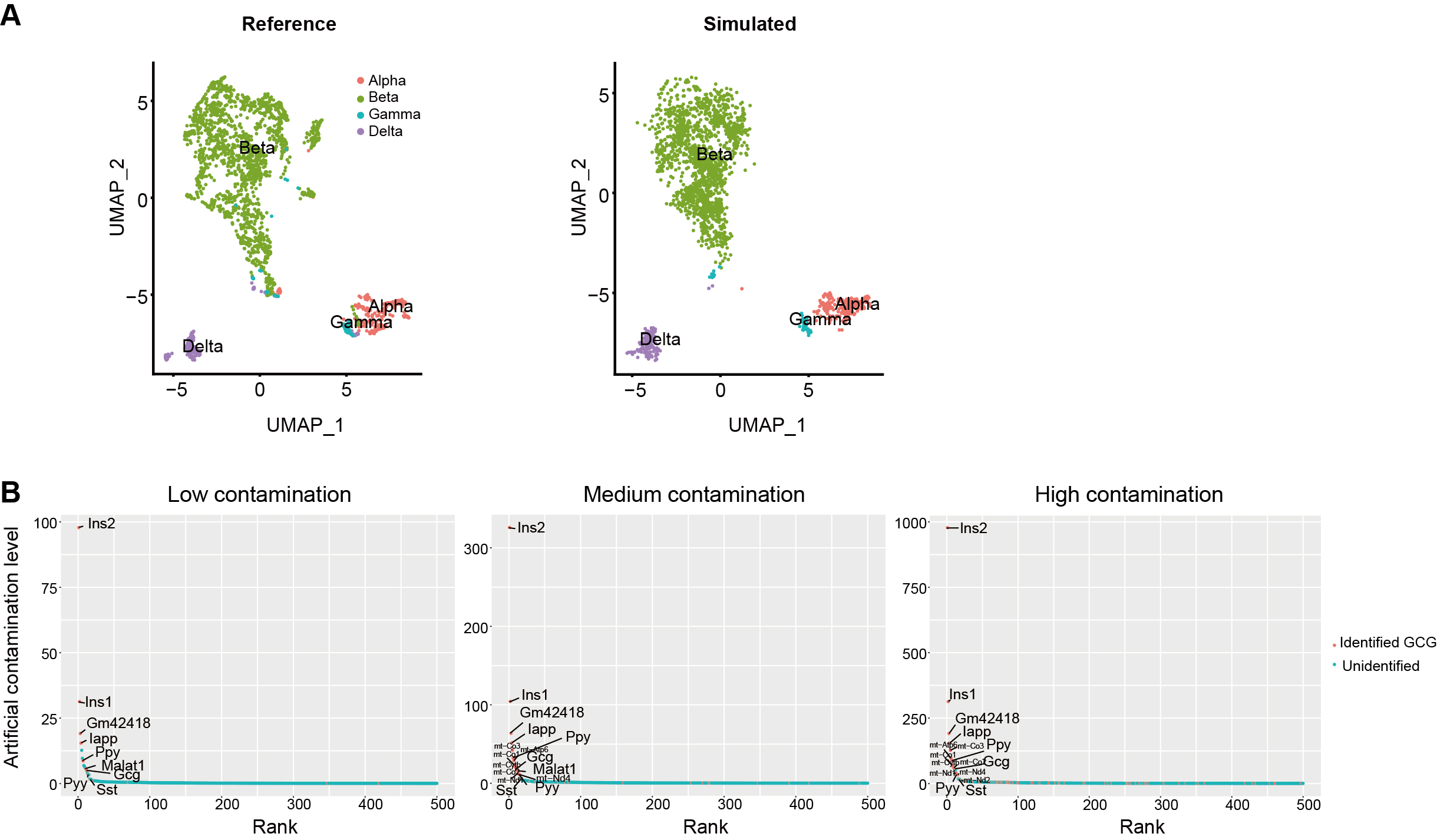


**Supplementary Figure 12.** scCDC identifies contamination in simulated pancreas data. (A) Cell clustering is barely affected by contamination. The cell clusters of filtered and spike-in corrected (Reference) and simulated uncontaminated datasets are shown in UMAP plots. (B) Top artificial contaminating genes are identified as GCGs. The plots show the artificial contamination vs. rank of 500 genes in the simulations in Figure 5. The GCGs identified by scCDC are highlight in red.


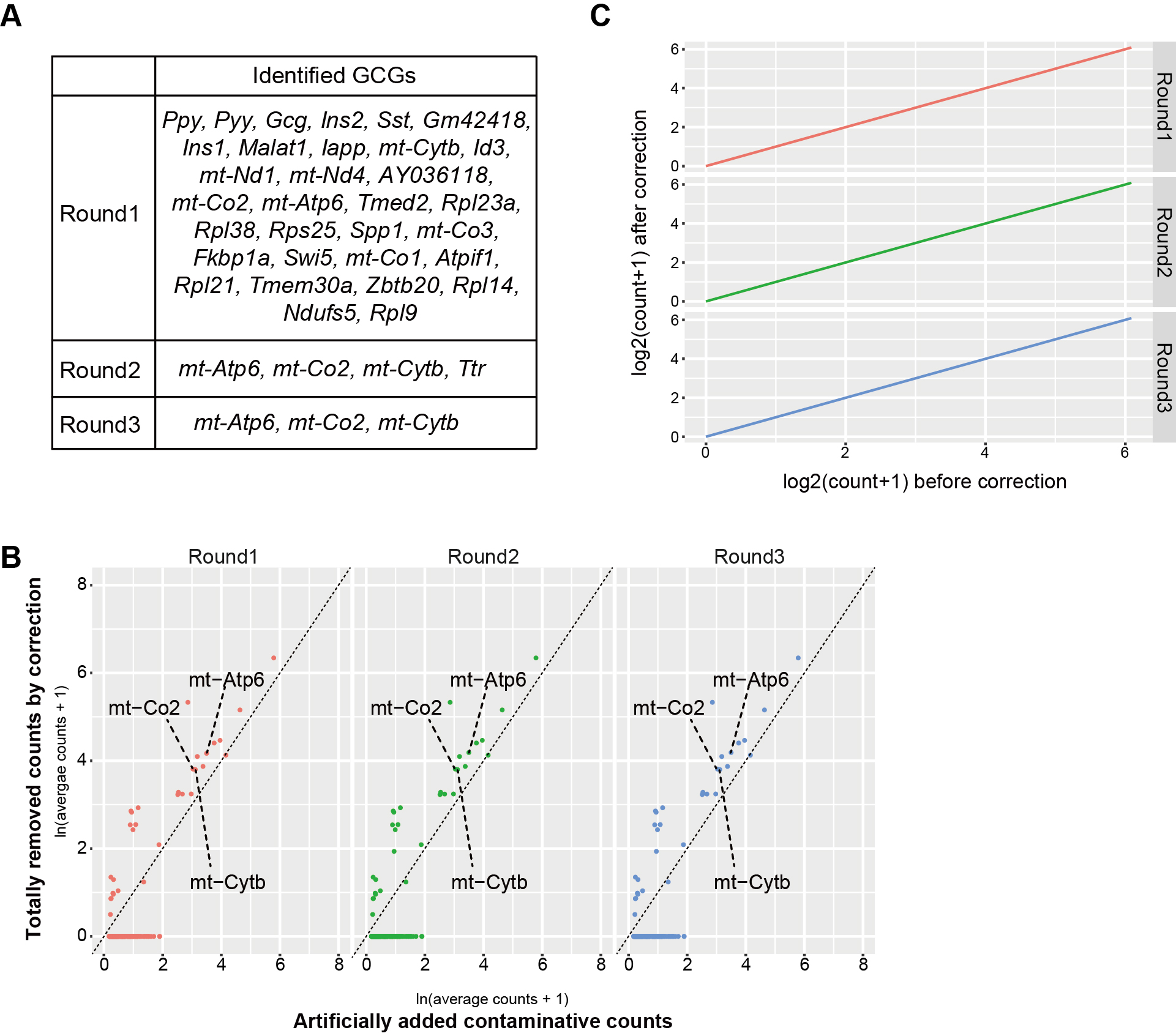


**Supplementary Figure 13.** One-time application of scCDC is sufficient for contamination correction in the simulated scRNA-seq dataset. The simulated scRNA-seq dataset with medium artificial contamination in Figure 5 was treated by scCDC iteratively. (A) The majority of GCGs are identified in the first run when scCDC is iteratively applied to the simulated pancreas dataset. The table presents the GCGs identified by scCDC in three iterative runs. (B-C) Iterative application of scCDC does not lead to over-correction. (B) No obvious correction is detected in GCGs. Scatter plots show the artificial contaminative counts vs. corrected counts of the 500 artificial contaminating genes in three iterative rounds of scCDC correction. (C) No obvious correction is detected in uncontaminated genes. Q-Q plots of the 95th percentile value of counts of 66 housekeeping genes in all the cells after each round of correction compared to original value of counts.


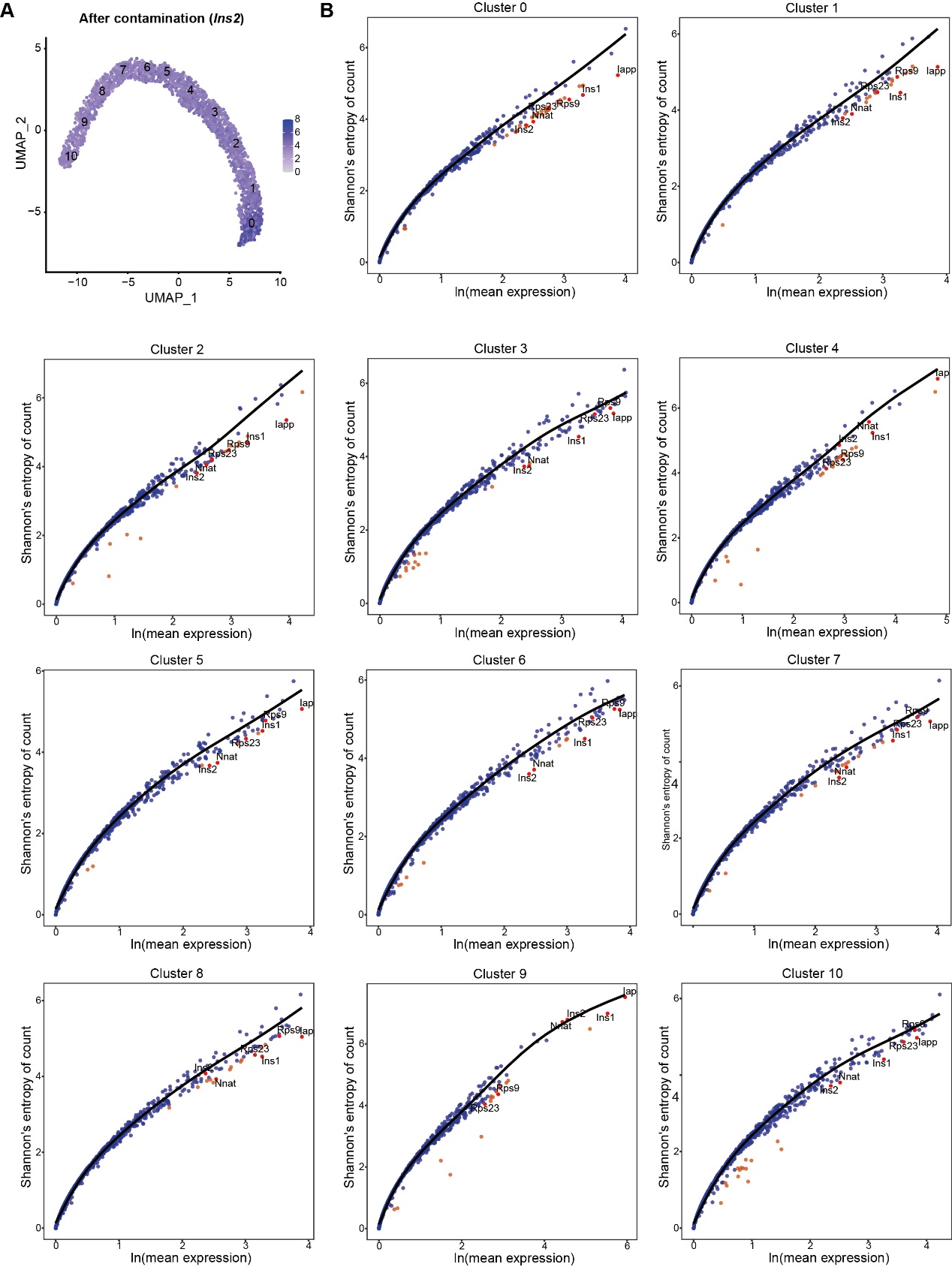


**Supplementary Figure 14.** scCDC identifies GCGs in simulated scRNA-seq trajectory dataset with contamination. (A) The UMAP shows cell clustering result in the simulated dataset by the default Seurat (V3). (B) The entropy-expression curves of indicated clusters. Identified candidate GCGs in each cluster are highlighted in orange and GCGs are highlight in red and labeled on the plots.


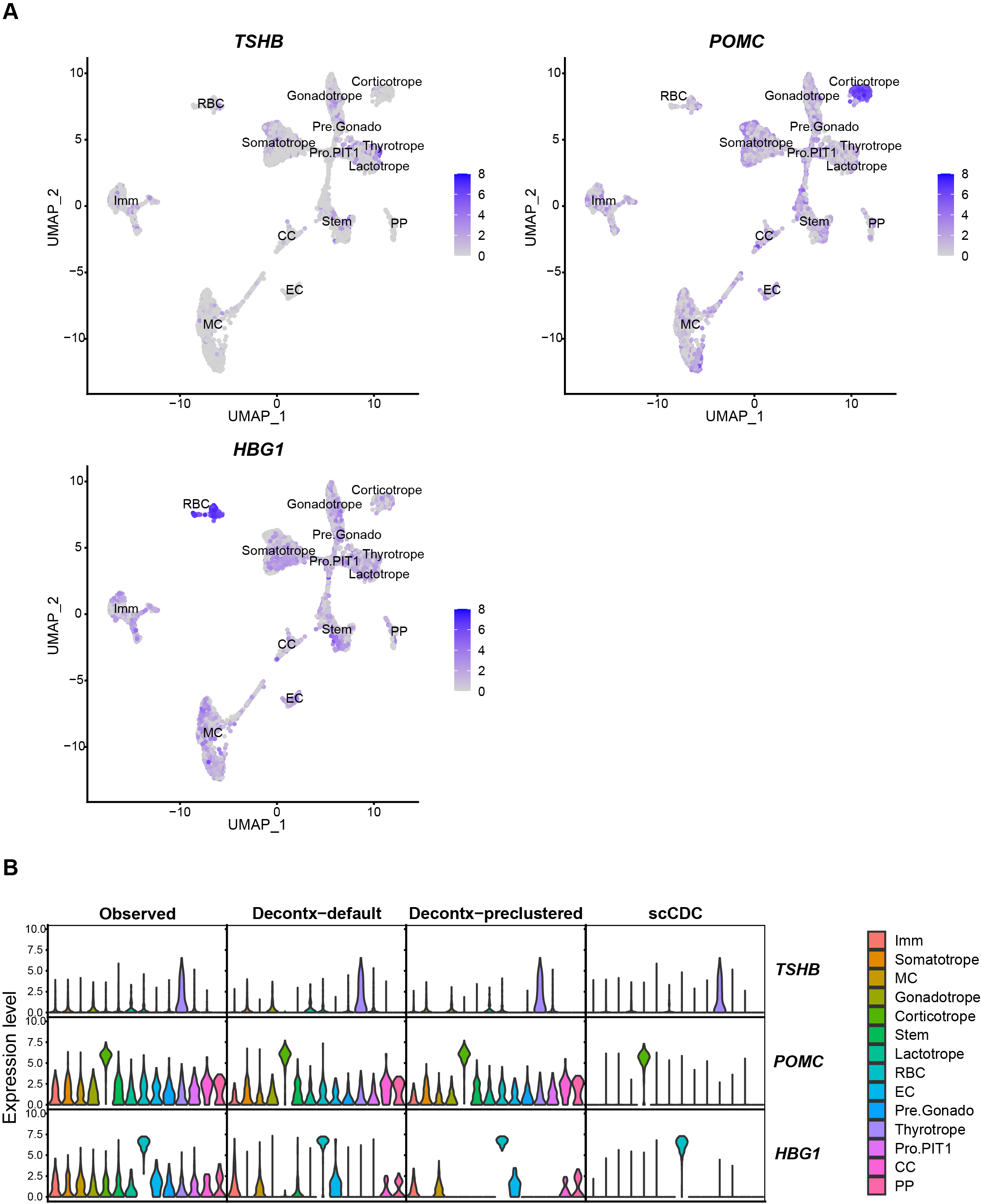


**Supplementary Figure 15.** scCDC efficiently corrects contamination in the scRNA-seq data in pituitary development. (A) The expression of thyrotrope marker *TSHB*, corticotrope marker *POMC* and RBC marker *HBG1* in the cells are shown in the UMAP plots. (B) The violin plots show the normalized expression levels of *TSHB*, *POMC* and *HBG1* before and after correction using the indicated methods by the default Seurat (V3).


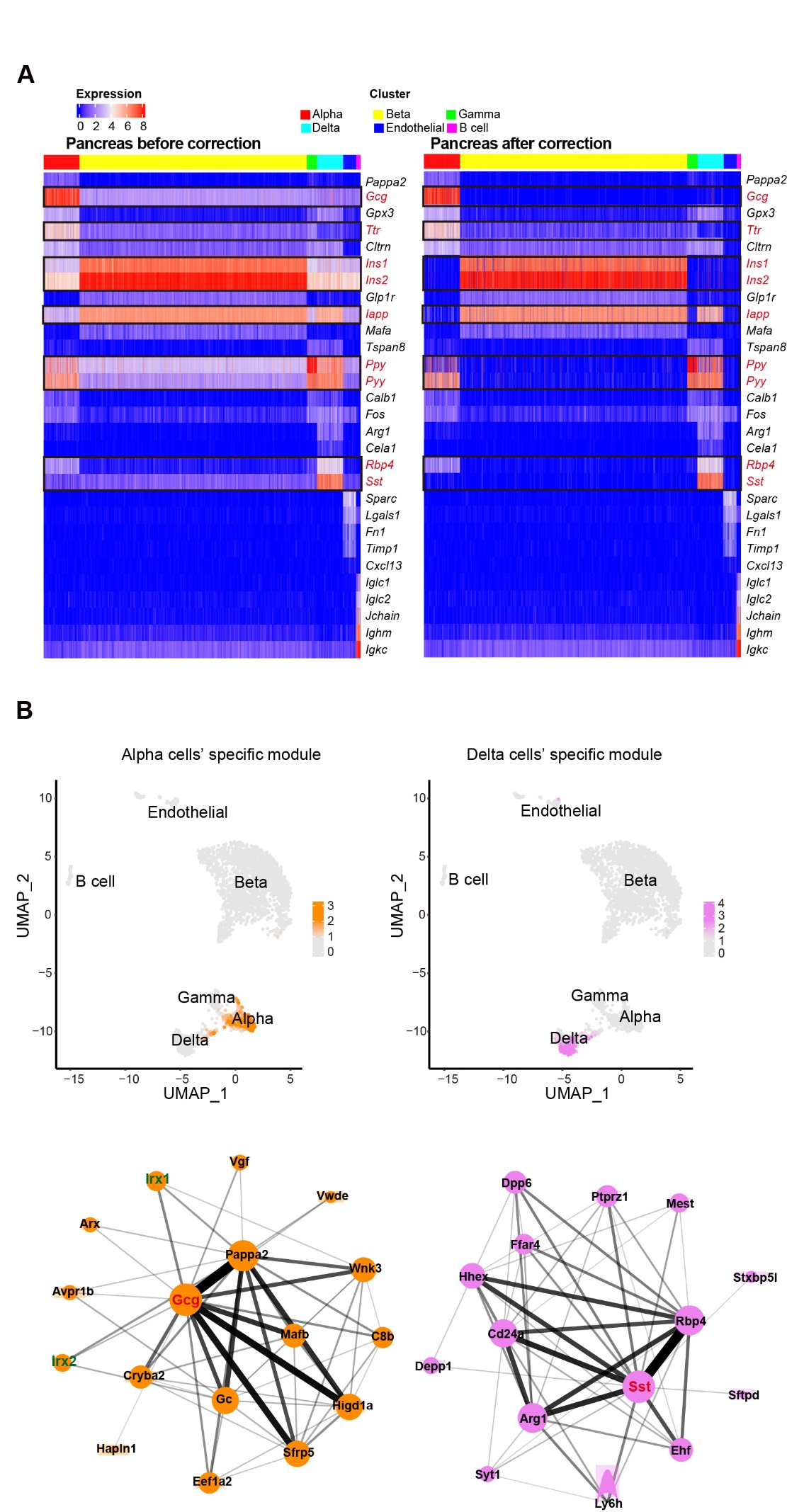


**Supplementary Figure 16.** scCDC improves marker gene profiling and gene network analysis in the mouse pancreas dataset. (A) Comparison of marker gene expression before (left) and after (right) correction in pancreas data is shown in heatmap. GCGs are highlighted in red. (B) The significant gene network modules associated with GCGs after correction were identified in pancreas. The mean expression of module genes are shown in UMAP plots and the gene network are also presented. GCGs are highlighted in red; over-corrected genes by scAR are highlighted in green.


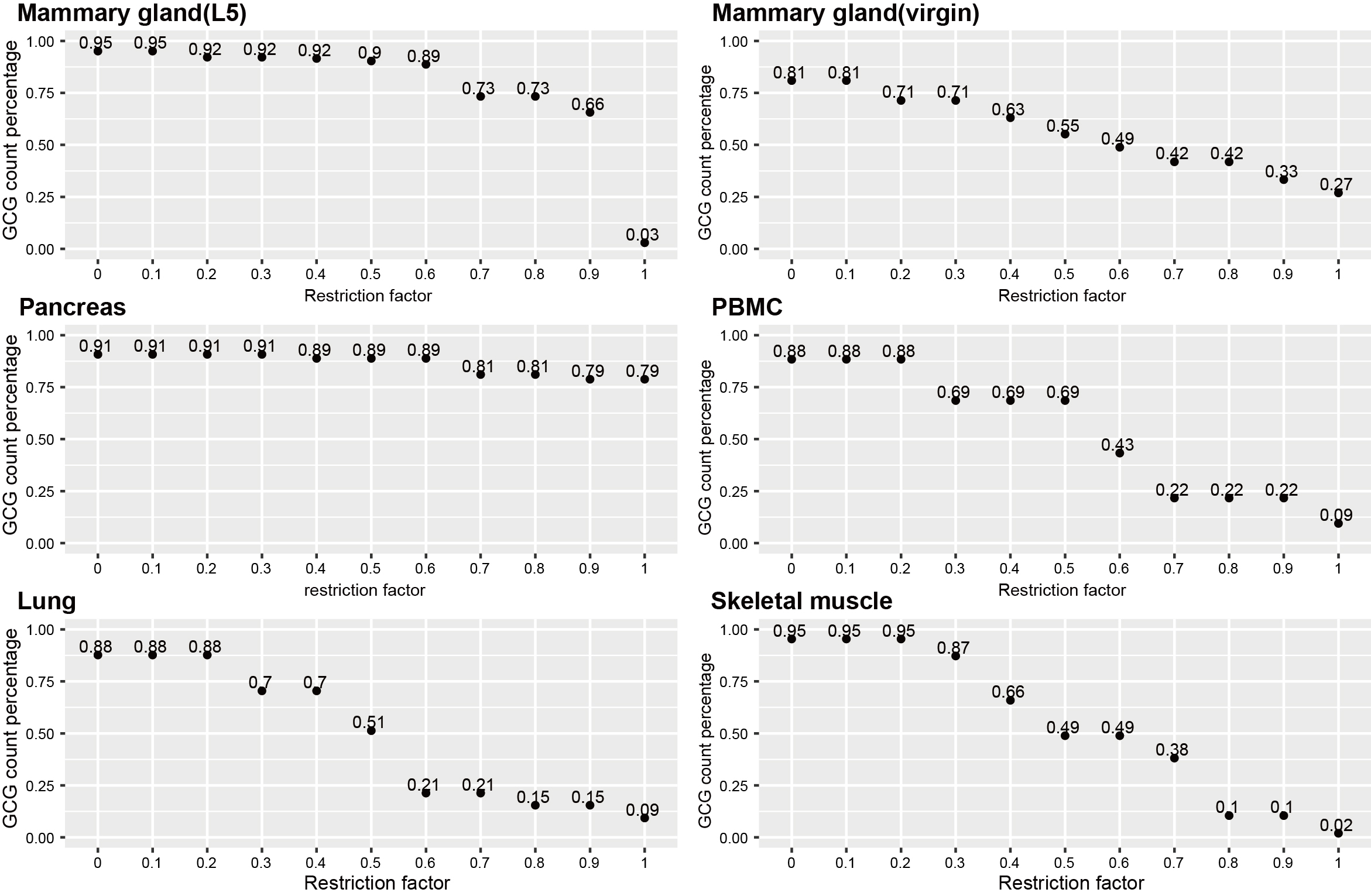


**Supplementary Figure 17.** The correlation between restriction factor and contamination identification in different datasets. The plots show the percentage of the counts of identified GCGs in the total counts of ‘super-contaminating’ genes in empty droplets at different restriction factor setting in the indicated datasets.


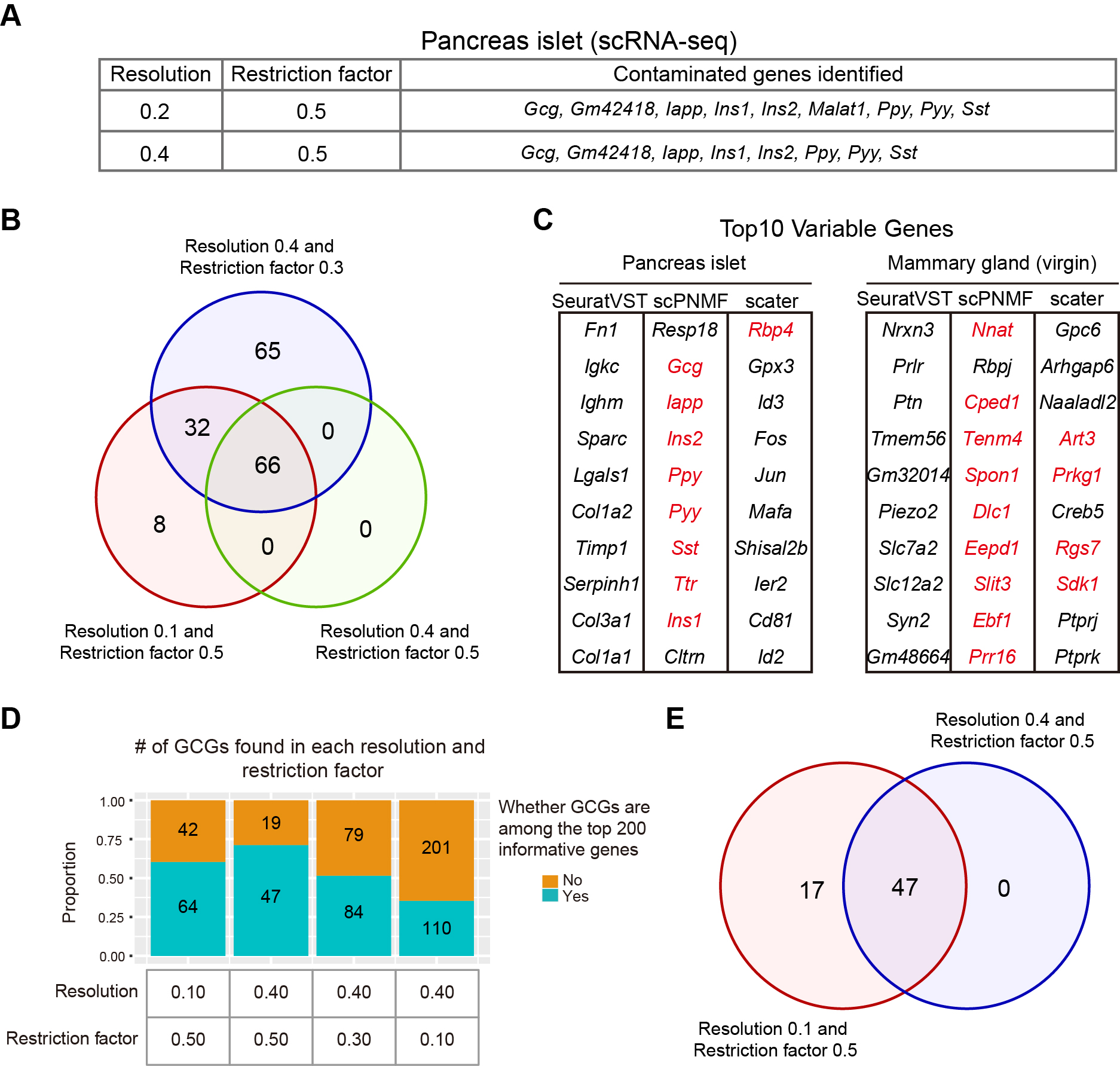


**Supplementary Figure 18.** scPNMF assists pre-clustering setting and GCG identification in contaminated datasets. (A) The list of GCGs identified by scCDC after pre-clustering with different resolutions in the mouse pancreas islet data. (B) Venn diagram shows the intersection of GCGs that are detected under different resolutions and restriction factors in the virgin mouse mammary gland snRNA-seq data. (C) Top 10 variable genes identified by SeuratVST, scPNMF, and Scater methods in pancreas islet scRNA-seq and virgin mammary gland snRNA-seq data. GCGs defined by scCDC are highlighted. (D) The barplot shows the percentage of scPNMF-determined cluster-informative genes in GCGs identified with indicated settings in the virgin mammary gland dataset as in (B). (E) Venn diagram shows the intersection of GCGs that are simultaneously identified as top 200 informative genes by scPNMF. Different groups of GCGs are detected under different resolutions and restriction factors.

**Method Appendix**

**The default restriction factor setting**

The counts of genes in the empty droplets in various datasets demonstrated that the contamination is dominated by a small group of genes (Supplementary Figure 3). Similar to this phenomenon, the epigenomic studies on enhancer markers revealed dominative enhancer signals (histone mark H3K27ac signal and mediator binding) on a small group of enhancers, namely super-enhancers [1]. Adopted the algorithm used in super-enhancer identification, we defined the ‘super-contaminative’ genes in empty droplets. Basically, the counts versus rank of detected genes in empty droplets were plotted and then an inflection point was determined. The genes above the inflection point were defined as ‘super-contaminative’ genes. We reasoned that the setting of restriction factor should enable finding more ‘super-contaminative’ genes while maximizing the power of restriction factor. Because the number of GCGs by scCDC increases when the restriction factor decreases, an optimal restriction factor would be set when further decreasing restriction factor does not significantly benefit identifying more contaminative counts contributed by ‘super-contaminative’ genes. Based on this idea, we calculated the percentage of counts of GCGs identified by scCDC in those of ‘super-contaminative’ genes in six datasets explored in this study (Supplementary Figure 17). We found that the restriction factor of 0.5 is suitable for most of the datasets. Therefore, we set 0.5 as default.

**Sensitivity analysis of scCDC to the resolution of cell pre-clustering**

Pre-clustering is a crucial step for the performance of DecontX [2]. Similar to DecontX, scCDC requires a pre-clustering step. To investigate the impacts of pre-clustering resolution on scCDC’s performance, we clustered the cells in the pancreas scRNA-seq dataset with different resolutions by the Louvain algorithm in Seurat V3 [3]. When the number of clusters increased from 9 to 10 (Louvain resolution from 0.2 to 0.4), the number of identified GCGs decreased from 9 to 8. To be noted, all the 8 genes were in the list of the 9 genes (Supplementary Figure 18A).

In the virgin mammary gland snRNA-seq dataset, increasing the Louvain resolution from 0.1 to 0.4 decreased the number of identified GCGs from 106 to 66, with 66 GCGs in common (Supplementary Figure 18B). A possible reason is that more clusters made fewer genes satisfy the default 50% restriction factor (i.e., GCGs must have statistically significant entropy divergences in more than 50% of the cell clusters; details in Methods). Hence, when the restriction factor was reduced to 30% at the Louvain resolution 0.4, more GCGs were detected (Supplementary Figure 18B). Notably, the GCGs identified in the settings were significantly overlapped (Supplementary Figure 18B). Taken together, these results indicated that the GCG identification by scCDC is sensitive to the pre-clustering resolution and an optimal restriction factor should be selected given a pre-clustering resolution.

**scPNMF assists scCDC in selecting the restriction factor given the pre-clustering resolution**

Therefore, we pursued to find a method to assist selecting the restriction factor in scCDC when a pre-clustering is performed with a given resolution. Because single-cell analysis largely relies on cluster-informative genes as shown in Figure 7, the targets of contamination correction should focus on these genes. Therefore, we reasoned that an appropriate pre-clustering setting is ought to maximize the proportion of cluster-informative genes in GCGs. However, in the contaminated pancreas and virgin mammary gland datasets, the widely used algorithms Seurat and Scater failed to identify GCGs as top informative genes, although most of which were known marker genes of various cell types. Recently, we developed scPNMF to unsupervisedly select cluster-informative genes in scRNA-seq data [4]. Unlike Seurat and Scater, scPNMF successfully identified the GCGs as the top 10 informative genes (Supplementary Figure 18C). Next, we examined the proportion of top 200 cluster-informative genes in the GCGs of the virgin mammary gland dataset, when different resolution and restriction factor were set in pre-clustering. The result showed that the combination of resolution/restriction factor of 0.4/0.5 led to maximal proportion of cluster-informative genes in identified GCGs (Supplementary Figure 18D). As expected, most of the GCGs were overlapped (Supplementary Figure 18E). These data indicated that scPNMF is a robust method in cluster-informative gene identification in contaminated datasets. It is suggested that scPNMF-identified top 200 cluster-informative genes can assist scCDC in selecting the restriction factor given a pre-clustering resolution.

**References:**

1. Whyte WA, Orlando DA, Hnisz D, Abraham BJ, Lin CY, Kagey MH, Rahl PB, Lee TI, Young RA: **Master transcription factors and mediator establish super-enhancers at key cell identity genes.** *Cell* 2013, **153:**307-319.

2. Yang S, Corbett SE, Koga Y, Wang Z, Johnson WE, Yajima M, Campbell JD: **Decontamination of ambient RNA in single-cell RNA-seq with DecontX.** *Genome biology* 2020, **21:**57.

3. Stuart T, Butler A, Hoffman P, Hafemeister C, Papalexi E, Mauck WMr, Hao Y, Stoeckius M, Smibert P, Satija R: **Comprehensive Integration of Single-Cell Data.** *Cell* 2019, **177:**1888-1902.e1821.

4. Song D, Li K, Hemminger Z, Wollman R, Li JJ: **scPNMF: sparse gene encoding of single cells to facilitate gene selection for targeted gene profiling.** *Bioinformatics (Oxford, England)* 2021, **37:**i358-i366.
