# Supplementary material for: scCDC: a computational method for gene-specific contamination detection and correction in single-cell and single-nucleus RNA-seq data
Source: Genome Biol. 2024 May 23;25:136. doi: 10.1186/s13059-024-03284-w (PMC11112958; doi:10.1186/s13059-024-03284-w)
Supplement: Supplementary file 8 — Supplementary Material 8. Review history. [file 13059_2024_3284_MOESM8_ESM.docx]

Review History

**First round of review**

**Reviewer 1**

**Were you able to assess all statistics in the manuscript, including the appropriateness of statistical tests used?**

**Yes:** see comments

**Were you able to directly test the methods?**

Yes

**Comments to author:**

From the manuscript, it appears scCDC was developed to address an issue encountered during the analysis of a specific snRNA-seq dataset generated from mouse mammary glands at the virgin and lactation stages. The authors observed cell-type marker genes in cell types in which they were not expected to be expressed. To address this issue, the authors applied existing ambient RNA correction methods but found the expression of these specific genes was not corrected to the degree expected. From this, the authors conclude these genes represent global contamination-causing genes, referred to as 'GCGs'. GCGs are defined as the most abundant ambient RNAs, and the authors develop a method to identify and correct just these genes.

As described in the SoupX, CellBender, scAR and DecontX software, the goal of removing ambient mRNA contamination from scRNA-seq datasets is to identify and remove the contribution of all cell-free RNAs from the gene expression profile of each cell. This is necessary for the biological interpretation of the resulting data, as downstream analysis methods assume a droplet contains only the RNA from a single cell. In my opinion, this goal is aligned with the biological reality of ambient RNA contamination. Ambient RNA released from dead, dying, or damaged cells are constituted by thousands of expressed genes, not the select few GCGs the authors focus on. This view is supported by a simple analysis of a 'barnyard' experiment in which cells from more than one species are mixed. The figure below describes the analysis of such a dataset, containing a half-half mixture of human HEK293T and mouse NIH3T3 cells. The results produced by scCDC identify just 9 genes as GCGs. These are the most abundant genes expressed from each (contaminating) species, as illustrated in the attached plot barnyard.pdf. Simply removing the expression of these 9 genes (from the offending species) does little to correct the ambient RNA contamination contributed by the thousands of other contaminating genes.

In my opinion, reframing the goal of addressing ambient RNA contamination to removing the contribution of a small number of GCGs leads to a flawed experimental methodology. When comparing the performance of scCDC against other methods, the authors compare the impact on just the chosen GCGs. However, the goal of the other four software is to remove the contribution of ambient contamination. A more appropriate performance evaluation metric would be to compare the total level of ambient RNA contamination remaining after applying each method. This leads to another criticism, which is the lack of appropriate experimental controls.

Throughout the study, the authors focus on a small number of marker genes that are not sufficiently validated to represent ambient RNA contamination. For instance, it is possible that the expression of these marker genes in unexpected cell types simply represents a low level of basal transcription. In this case, it is unclear that scCDC is correctly removing the contributions of these genes. The authors include an analysis of data from Sanchez et al. which included spike-in cells but again focus only on GCGs. To accurately benchmark the performance of scCDC, appropriate controls are required to determine whether only ambient RNA is being removed.


● The authors comment in the background section, page 2 lines 54-58, that ambient RNA is likely to present more of a problem in snRNA-seq compared to scRNA-seq datasets. The authors follow up on this comment with the analysis presented in Fig 5F. This argument would be strengthened if the authors could provide a comparison between cells and nuclei from the same sample type.
● The authors comment that ambient RNA contamination of snRNA-seq datasets must be removed post-hoc (page 3, line 6). It would be useful if the authors could comment on the impact of sorting, which is frequently employed to remove ambient RNA from single nuclei suspensions prior to capture.
● The authors speculate that because SoupX, CellBender, scAR and DecontX do not distinguish GCGs this may lead to over-correction (page 3, lines 39-44). This statement should be elaborated on to explain why the authors believe this to be an issue and provide a rationale for why they have chosen to focus on a small number of genes (GCGs).
● When discussing the results in Fig 1E (page 3: lines 42-44 and page 5: lines 34-49) the authors claim SoupX and scAR over-corrected the expression of housekeeping genes. This is unclear, as the authors do not demonstrate these genes do not contribute to the pool of ambient RNA. Further, it would improve readability if the text in the background section was moved to the results section.
● The authors describe an assumption of scCDC that "most genes produce little or no ambient RNAs". This is a key assumption and should be justified in the main text.
● A description of the empirical results that informed the choice of a restriction factor parameter of 80% should be provided
● A simulated experiment is described on pages 5-6, in which counts from three genes; LYZ, S100A8 and S100A9 are simulated as ambient RNA contamination. In addition to scCDC, the authors apply DecontX. As DecontX was developed to deal with (biologically relevant) global ambient RNA contamination, this does not appear to be a valid comparison.

**Reviewer 2**

**Were you able to assess all statistics in the manuscript, including the appropriateness of statistical tests used?**

**Yes:** The statistical method is rigorous

**Were you able to directly test the methods?**

Yes

**Comments to author:**

The paper addresses the problem of detecting systematic contamination of ambient RNA molecules in droplet-based single-cell RNA sequencing (scRNA-seq) and single-nucleus RNA sequencing (snRNA-seq) assays. Existing computational methods to correct this contamination do not distinguish the contamination-causing genes, leading to either under- or over-correction of the contamination. Therefore, the authors developed scCDC, the first method that detects the contamination-causing genes and corrects only the expression levels of these genes. The authors benchmarked scCDC against existing methods on synthetic and real scRNA-seq and snRNA-seq datasets and claimed that it achieved the best contamination correction accuracy with minimal data alteration. The paper has a focused and important task and presents technical novelty.
While the general idea is attractive, this paper still has several limitations: some statements lack supporting evidence, the assumption of cell identity information is too strong, and some details should be added. Below are my comments:

Major Comments:

1. My major concern is that the method requires pre-clustering, which is not trivial for many tissues. Although some discussion was made in supplemental materials, it requires further justification. In addition, this additional step will limit the use of the method in practice compared to other existing methods.

2. The first assumption of the method is that most genes produce little or no ambient RNAs. It is not well justified in the paper. Is it common for most scRNA-seq or snRNA-seq experiments or only for mouse mammary gland datasets that were shown?

3. For simulated data, the authors show that the method's detection ability is correlated to the contamination level, which is expected. I wonder what would happen if the data or the real situation was not under your assumption "cell type information is given". What would happen if cell types are not well separated i.e. heterogeneous tissue from developmental biology? What would happen if some cells are mis-classified in real data analysis where "true" cell type labels are not available before you remove the contaminated data. Although the authors provide pre-clustering results with different resolutions, in synthetic data, they could provide more information like ARI/NMI to assess the clustering accuracy or use ROGUE to assess the clustering purity. More results are needed to show the robustness of the method.

4. The benchmarking against other existing methods is mostly evaluated in synthetic data or in-house mouse mammary glands. It is interesting to see the performance of the proposed methods in the benchmarking datasets presented in competing methods such as DecontX and SoupX.

5. The authors describe the motivation of developing such algorithm to remove ambient RNA by introducing the sequencing results of snRNA-seq of L5 mouse mammary gland (Section 1 in Results), however, details are lacking to support the statement. For example, some references are needed to claim "Wap and Csn2, which encode the whey acidic protein and the casein protein respectively, should be expressed exclusively in the differentiated alveolar epithelial cells (AlveoDiff) during lactation".

6. Bulk sequencing results and the corresponding snRNA-seq results are a bit confusing. The paper claims Was gene should be exclusively expressed in AlveoDiff cells, but the bulk sequencing results suggest that Was is abundant in this tissue. So, does it mean a very small cell population (AlveoDiff in this example), could contribute such a global contamination with such a high expression level. Why can't this gene be uniformly expressed in other cells like AlveoProg? There may exist bias for snRNA-seq to select cells, so the amount of AlveoDiff is very small? Cell sorting like Fluorescence-activated cell sorting (FACS) could be used to explore this issue.

7. I appreciate that the authors tested the method on 12 datasets and listed all cell-type-specific GCGs assuming the proposed method works properly. However, I am not convinced about the conclusion that contamination is more severe in snRNAseq
data than in scRNA-seq data based on Wasserstein distances across different tissues. The tissue heterogeneity should be considered. Even for the same tissue, mammary glands before and after lactating, may be different.

Minor Comments:

8. Benchmarking codes should be publicly available.
9. Some typos should be fixed.
10. Figure 4b, the Y-axis is the average entropy divergence across all cell types. This needed to be clarified.

**Authors Response**

**Point-by-point responses to the reviewers’ comments:**

**Reviewer #1:** From the manuscript, it appears scCDC was developed to address an issue encountered during the analysis of a specific snRNA-seq dataset generated from mouse mammary glands at the virgin and lactation stages. The authors observed cell-type marker genes in cell types in which they were not expected to be expressed. To address this issue, the authors applied existing ambient RNA correction methods but found the expression of these specific genes was not corrected to the degree expected. From this, the authors conclude these genes represent global contamination-causing genes, referred to as 'GCGs'. GCGs are defined as the most abundant ambient RNAs, and the authors develop a method to identify and correct just these genes.

Reviewer

As described in the SoupX, CellBender, scAR and DecontX software, the goal of removing ambient mRNA contamination from scRNA-seq datasets is to identify and remove the contribution of all cell-free RNAs from the gene expression profile of each cell. This is necessary for the biological interpretation of the resulting data, as downstream analysis methods assume a droplet contains only the RNA from a single cell. In my opinion, this goal is aligned with the biological reality of ambient RNA contamination. Ambient RNA released from dead, dying, or damaged cells are constituted by thousands of expressed genes, not the select few GCGs the authors focus on. This view is supported by a simple analysis of a 'barnyard' experiment in which cells from more than one species are mixed. The figure below describes the analysis of such a dataset, containing a half-half mixture of human HEK293T and mouse NIH3T3 cells. The results produced by scCDC identify just 9 genes as GCGs. These are the most abundant genes expressed from each (contaminating) species, as illustrated in the attached plot barnyard.pdf. Simply removing the expression of these 9 genes (from the offending species) does little to correct the ambient RNA contamination contributed by the thousands of other contaminating genes.

Response

In this revised manuscript, we follow the suggestion and benchmark the performance of all the methods in the 'barnyard' experiment of mixed human and mouse cells, and demonstrate that scCDC successfully identifies the top contamination-causing genes, and its correction sufficiency is secondary to DecontX, and outperforms the other methods in this specific dataset. These results are included on Page 10 and presented in Figure 5A and Supplementary Figure 5. **Notably, the cross-contamination, especially the human gene contamination in mouse cells is low in general in this dataset. In highly contaminated datasets, such as scRNA-seq in pancreas and liver, snRNA-seq in mammary gland, DecontX, SoupX-automated and CellBender could not provide sufficient correction. In contrast, scCDC provides sufficient correction of contamination-causing genes but not over-corrects the other genes** (Figure 1D-E, Figure 5C-E and Supplementary Figure 9).

Although we agree with the reviewer that the ambient RNAs can come from any genes, our analysis of multiple datasets, including the mixed-species dataset the reviewer analyzed, suggests that the ambient RNAs are dominated by a small number of genes. We examined the counts of all genes in the empty droplets in these datasets and clearly showed that **the ambient RNAs in the ‘soup’ are actually from a small group of genes, from dozens to hundreds, dominantly; while scCDC successfully identified the majority of these genes**. The data are now shown in Supplementary Figure 3, 5 &6 (See below).

The marginal contamination from the other genes may barely affect downstream analysis. As the majority of genes contribute little to the contamination (as we have shown in our analysis of 6 datasets in Supplementary Figure 3, see below), global contamination correction is prone to over-correct these genes, as demonstrated in the benchmarking analysis in pancreas, PBMC, mammary gland and simulated datasets in Figures 1, 5 and 6 and Supplementary Figure 2, 6&8.

Based on these observations, we propose this new strategy of identifying and only correcting GCGs. Compared to current methods, our approach avoids under-correction of the dominating contamination-causing genes and over-correction of marginal/non-contaminated genes. We notice that our conservative strategy may not be able to identify some lowly contaminative genes and only slightly change cell clustering result. Considering that DecontX has good performance on correcting lowly-contaminated genes, integration of scCDC and DecontX is an option for future exploration. We have included these in the discussion on Page 17.

Reviewer

In my opinion, reframing the goal of addressing ambient RNA contamination to removing the contribution of a small number of GCGs leads to a flawed experimental methodology. When comparing the performance of scCDC against other methods, the authors compare the impact on just the chosen GCGs. However, the goal of the other four software is to remove the contribution of ambient contamination. A more appropriate performance evaluation metric would be to compare the total level of ambient RNA contamination remaining after applying each method. This leads to another criticism, which is the lack of appropriate experimental controls.

Response

We appreciate this suggestion. Notably, it is impossible to evaluate the total level of ambient RNA contamination in most of the real datasets, due to the lack of ground truth. Therefore, we examined the total level of remaining contamination in the dataset of mixed human and mouse cells and the simulated datasets we generated. As stated above, scCDC’s correction sufficiency is secondary to DecontX and outperforms the other methods in the dataset of mixed cells (Figure 5A and Supplementary Figure 5). Of note, scAR showed over-correction of lowly-expressed genes (Supplementary Figure 5C).

In the simulated PBMC dataset, scCDC corrects all the artificial contaminative counts as shown in Figure 3D.

In addition, we newly simulate an uncontaminated pancreas single-cell dataset mimicking the real datasets and then simulated low, medium and high level of contamination of 500 genes to test the robustness of the methods and answer the question why DecontX’s performance varies in datasets. In the low contamination scenario, DecontX-preclustered successfully removes the artificial contaminative counts, although the decontamination of the top contaminative genes is less sufficient. Applied on the medium and high contamination datasets, DecontX exhibits obvious under-correction. These results indicate that **DecontX is sensitive to contamination level, providing sufficient correction for lowly-contaminative genes, but insufficient correction for highly-contaminative genes**. Interestingly, DecontX also removes a small amount of counts of uncontaminated genes when the contamination level is high, suggesting a possible over-correction on these genes. **In contrast, scCDC is insensitive to contamination level and provides robust corrections of identified GCGs.** And scCDC does not alter the counts of uncontaminated genes as designed. Of note, scCDC identified all the top artificial contaminative genes, but not all the 500 genes as GCGs, possibly due to the contamination level of these genes being relatively low, and the entropy divergence alteration of these genes being small. Because the other methods require empty-droplets data, we cannot examine their performance in the simulated datasets. These data are now included in the result from Page 13, and presented in Figure 6 (See below) and Supplementary Figure11.

Reviewer

Throughout the study, the authors focus on a small number of marker genes that are not sufficiently validated to represent ambient RNA contamination. For instance, it is possible that the expression of these marker genes in unexpected cell types simply represents a low level of basal transcription. In this case, it is unclear that scCDC is correctly removing the contributions of these genes. The authors include an analysis of data from Sanchez et al. which included spike-in cells but again focus only on GCGs. To accurately benchmark the performance of scCDC, appropriate controls are required to determine whether only ambient RNA is being removed.

Response

The contamination in our dataset and public pancreas one is **not just a low level of basal transcription of the genes**. For example, the **exclusive expression of milk protein-coding genes, like *Wap* and *Csn2* in differentiated alveolar cells have been demonstrated by staining of the tissue using Wap-LacZ reporter mouse line and RNA in situ hybridization assays** in previous studies [1, 2]. Similarly, the **exclusive expression of *Ins1* and *Ins2* in Beta cells are also demonstrated** decades ago [3]. These evidences are well acknowledged and written in the textbooks. Whereas the contamination caused by these genes is corrected insufficiently by DecontX and CellBender.

Following the reviewer’s suggestion, we also simulate and artificially contaminate a pancreas dataset mimicking the real dataset but with ground truth. We now show that scCDC completely removes contaminative counts of identified GCGs. The correction may be slightly over in some genes. These results are included in the manuscript from Page 13 and Figure 6.

Reviewer

The authors comment in the background section, page 2 lines 54-58, that ambient RNA is likely to present more of a problem in snRNA-seq compared to scRNA-seq datasets. The authors follow up on this comment with the analysis presented in Fig 5F. This argument would be strengthened if the authors could provide a comparison between cells and nuclei from the same sample type.

Response

Following the suggestion, now we compare the snRNA-seq data generated in this study and published scRNA-seq dataset in lactating mammary gland, and we show the ratio of GCG expression in eGCG- vs. eGCG+ cells, which represents the proximity of expression from ambient and endogenous RNAs, is higher in snRNA-seq than that in scRNA-seq data (Supplementary Figure 10, see below). This result supports our comment that ambient RNA is likely to present more of a problem in snRNA-seq.

Reviewer

The authors comment that ambient RNA contamination of snRNA-seq datasets must be removed post-hoc (page 3, line 6). It would be useful if the authors could comment on the impact of sorting, which is frequently employed to remove ambient RNA from single nuclei suspensions prior to capture.

Response

Following this suggestion, we have generated another snRNA-seq dataset in virgin mammary gland, employing intensive sorting in the experiment. We show that the sorting step can mildly improve the result, but the contamination still exists. The data is now included on Page 4-5 and shown in Supplementary Figure 1 (See below).

Reviewer

The authors speculate that because SoupX, CellBender, scAR and DecontX do not distinguish GCGs this may lead to over-correction (page 3, lines 39-44). This statement should be elaborated on to explain why the authors believe this to be an issue and provide a rationale for why they have chosen to focus on a small number of genes (GCGs).

Response

The reason we focus on GCGs and avoids over-correction is that potential **over-correction of non-contamination-causing genes, especially the ones expressed at low levels, will probably lead to dropout of informative genes or incorrect profiling these genes in cell clusters.** Therefore, downstream analysis like WGCNA would be significantly affected. This rationale is included in the introduction on Page 3 now.

For example, the *Irx1* and *Irx2* are Alpha cell marker genes [4]. However, the counts of *Irx1* and *Irx2* are completely wiped out by scAR (Figure 5E). The dropout of these two marker genes would lead to incomplete gene network identification, as *Irx1* and *Irx2* are supposed to be in the Alpha cell specific network module in WGCNA shown in Figure 7C. In addition, we found that over-correction, especially by SoupX-manual and scAR occurs on a lot of housekeeping genes, including ribosomal protein-coding genes (Supplementary Figure 2, 6B & 8B), which also leads to insufficient identification of gene networks. As we show in Figure 7D, the WGCNA in lactating mammary gland data showed a regulatory module consisting of milk-protein coding genes and ribosome genes in L5 mammary AveoDiff. This is in line with the biological fact that translation machinery is extensively active to produce large amount of milk proteins in the differentiated alveolar cells at lactation [5, 6]. However, the majority of these genes’ counts are overcorrected or even cleared out by SoupX and scAR, leading to the identification of incomplete gene network.

In addition, scAR showed over-correction of lowly-expressed genes in the ‘barnyard’ dataset of mixed human and mouse cells (Supplementary Figure 5). In our simulated pancreas dataset, DecontX tends to over-correct lowly-expressed genes when the contamination level is high (Figure 6C, see above).

Reviewer

When discussing the results in Fig 1E (page 3: lines 42-44 and page 5: lines 34-49) the authors claim SoupX and scAR over-corrected the expression of housekeeping genes. This is unclear, as the authors do not demonstrate these genes do not contribute to the pool of ambient RNA. Further, it would improve readability if the text in the background section was moved to the results section.

Response

It is possible that some housekeeping genes may also contribute to the pool of ambient RNA. Actually, the data in empty droplets also suggest that some housekeeping genes are also contamination-causing genes, and can be identified by scCDC (Supplementary Figure 3). However, certain amount of housekeeping genes are in the group of marginal/non- contaminative group, whose counts are significantly over-corrected. For example, *Rps14* and *Rps8* in lactating mammary gland and *Rpl27* and *Rpl37* are over-corrected by SoupX and scAR ash shown in Figure 1E, while they are unlikely contamination-causing genes as shown in Supplementary Figure 3.

Thanks to the suggestion of moving the text in the background section to the results section. However, since these data are our motivation of developing scCDC, we think it is proper to keep them in the background.

Reviewer

The authors describe an assumption of scCDC that “most genes produce little or no ambient RNAs”. This is a key assumption and should be justified in the main text.

Response

We thank the reviewer for the suggestion. Now we show **the counts of genes in the ‘soup’, represented by the data in empty droplets, are dominantly contributed by a small group of genes** (Supplementary Figure 3, see above), which supports our assumption that “most genes produce little or no ambient RNAs”.

Reviewer

A description of the empirical results that informed the choice of a restriction factor parameter of 80% should be provided.

Response

Thanks to the reviewer’s comment, based on our comprehensive new analysis from 6 datasets, we have refined the restriction factor value to 50%, so scCDC can detect more GCGs that have caused the contamination. The restriction factor is also an open parameter and could be adjusted by users. The default value is based on the empirical results in multiple datasets that enable finding more ‘super-contaminative’ genes while maximizing the power of restriction factor. The details are included in the method appendix in Additional file 1 now.

Reviewer

A simulated experiment is described on pages 5-6, in which counts from three genes; LYZ, S100A8 and S100A9 are simulated as ambient RNA contamination. In addition to scCDC, the authors apply DecontX. As DecontX was developed to deal with (biologically relevant) global ambient RNA contamination, this does not appear to be a valid comparison.

Response

We agree that it is inappropriate to compare DecontX and scCDC in the dataset. Now we remove the result from DecontX.

**Reviewer #2:** The paper addresses the problem of detecting systematic contamination of ambient RNA molecules in droplet-based single-cell RNA sequencing (scRNA-seq) and single-nucleus RNA sequencing (snRNA-seq) assays. Existing computational methods to correct this contamination do not distinguish the contamination-causing genes, leading to either under- or over-correction of the contamination. Therefore, the authors developed scCDC, the first method that detects the contamination-causing genes and corrects only the expression levels of these genes. The authors benchmarked scCDC against existing methods on synthetic and real scRNA-seq and snRNA-seq datasets and claimed that it achieved the best contamination correction accuracy with minimal data alteration. The paper has a focused and important task and presents technical novelty.

While the general idea is attractive, this paper still has several limitations: some statements lack supporting evidence, the assumption of cell identity information is too strong, and some details should be added. Below are my comments:

Response

We are grateful to this reviewer appreciating the importance and novelty of our work.

Major Comments:

Reviewer

1. My major concern is that the method requires pre-clustering, which is not trivial for many tissues. Although some discussion was made in supplemental materials, it requires further justification. In addition, this additional step will limit the use of the method in practice compared to other existing methods.

Response

We agree that pre-clustering is not trivial for many tissues. However, cell clustering is a necessary step in most of the single-cell data analysis. Although the methods like DecontX and SoupX do not forcibly require preclustering step, it is noticed that **preclustering in DecontX and manually pre-defining contaminative genes after preclustering in SoupX strikingly improved the performance of the two methods** in all the tests we have done (Figure 1, 5 and Supplementary Figure 9). In the default and automated setting, DecontX and SoupX failed to give sufficient correction, which is consistent with the result in a very recent report [7]. These results again suggest the necessity of supervised, at least half-supervised cell clustering before contamination correction. To be noted, the identification of known cell types is not significantly affected by ambient RNA contamination, at least in the datasets we have tested. And a number of cell-type annotation tools (Azimuth [8], SingleR [9], Cell Blast [10], SciBet [11]) and databases (CellMarker [12], PanglaoDB [13]) have been developed to help classify cell types. For example, the NIH HuBMAP consortium has released Azimuth, which provides reference cell types for many human tissues (https://azimuth.hubmapconsortium.org). Moreover, novel tools like scDesign3 can be used to justify the preclustering accuracy. We have included this in the discussion section on Page 17 now.

Reviewer

2. The first assumption of the method is that most genes produce little or no ambient RNAs. It is not well justified in the paper. Is it common for most scRNA-seq or snRNA-seq experiments or only for mouse mammary gland datasets that were shown?

Response

We examined the counts of all genes in the empty droplets in multiple datasets and clearly showed that **the ambient RNAs in the ‘soup’ are actually from a small group of genes, from dozens to hundreds, dominantly; while scCDC successfully identified the majority of these genes.** The data are now included on Page 6 and shown in the Supplementary Figure 3 (see above). The marginal contamination from the other genes may barely affect downstream analysis. Based on these observations, we propose this new strategy of identifying and only correcting GCGs.

Reviewer

3. For simulated data, the authors show that the method's detection ability is correlated to the contamination level, which is expected. I wonder what would happen if the data or the real situation was not under your assumption "cell type information is given". What would happen if cell types are not well separated i.e. heterogeneous tissue from developmental biology? What would happen if some cells are mis-classified in real data analysis where "true" cell type labels are not available before you remove the contaminated data. Although the authors provide pre-clustering results with different resolutions, in synthetic data, they could provide more information like ARI/NMI to assess the clustering accuracy or use ROGUE to assess the clustering purity. More results are needed to show the robustness of the method.

Response

We thank the reviewer for the suggestion. To test if our method is robust when cell types are not well separated, we applied scCDC and DecontX in the published dataset from the experiment in pituitary development, which contained complex and not-well separated clusters. Our data showed that scCDC works well in the dataset and identifies GCGs of *TSHB*, *POMC* and *HBG1*, and corrected their contamination. The data is shown in Supplementary Figure 14 (see below).

We also simulated an uncontaminated pancreas developmental trajectory dataset without any cell clustering information by scDesign3, and artificially added contamination. After default pre-clustering in Seurat, scCDC also successfully corrected the data (Supplementary Figure 13, see below). These data demonstrate the robustness of scCDC.

We also follow the suggestion by clustering our synthetic PBMC dataset at different clustering resolutions and then calculating ARI to assess the clustering accuracy. While the ARI varies as expected, the correction by scCDC does not change. The reason is that scCDC identifies eGCG- clusters and estimates the contamination contribution in the grouped eGCG- clusters, which reduces the influence of clustering resolution. These data are included in the result on Page 9 and Supplementary Table 1.

Reviewer

4. The benchmarking against other existing methods is mostly evaluated in synthetic data or in-house mouse mammary glands. It is interesting to see the performance of the proposed methods in the benchmarking datasets presented in competing methods such as DecontX and SoupX.

Response

Following the suggestion, we now benchmark the methods in more datasets, including the datasets from human-mouse mixed cells, PBMC-4K and liver tissue. In the human-mouse mixed cell dataset, scCDC is secondary to DecontX and outperforms the other methods (Figure 5A). In the PBMC-4K dataset, every method successfully corrects the contamination of LYZ, but SoupX and scAR showed over-correction of housekeeping genes (Figure 5B and Supplementary Figure 6B). In the liver dataset, scCDC, SoupX-manual and scAR provided sufficient correction; while under-correction was found in DecontX and CellBender corrected data (Supplementary Figure 9), which is similar to the findings in the mammary gland and pancreas data.

Reviewer

5. The authors describe the motivation of developing such algorithm to remove ambient RNA by introducing the sequencing results of snRNA-seq of L5 mouse mammary gland (Section 1 in Results), however, details are lacking to support the statement. For example, some references are needed to claim "Wap and Csn2, which encode the whey acidic protein and the casein protein respectively, should be expressed exclusively in the differentiated alveolar epithelial cells (AlveoDiff) during lactation".

Response

The experimental evidences were well documented. Previous studies employing genetic reporter mouse strain and RNA in situ hybridization demonstrate that *Wap* and *Csn2* are expressed exclusively in the differentiated alveolar epithelial cells (AlveoDiff) during lactation. The references [1, 2] are added in the result on Page 4 now.

Reviewer

6. Bulk sequencing results and the corresponding snRNA-seq results are a bit confusing. The paper claims Wap gene should be exclusively expressed in AlveoDiff cells, but the bulk sequencing results suggest that Wap is abundant in this tissue. So, does it mean a very small cell population (AlveoDiff in this example), could contribute such a global contamination with such a high expression level. Why can't this gene be uniformly expressed in other cells like AlveoProg? There may exist bias for snRNA-seq to select cells, so the amount of AlveoDiff is very small? Cell sorting like Fluorescence-activated cell sorting (FACS) could be used to explore this issue.

Response

Thanks to the comment. We also examined the published scRNA-seq dataset in lactating mammary gland, showing that the AlveoDiff is a small population (Supplementary Figure 10A), which is consistent with our snRNA-seq dataset. **As shown in the previous studies using transgenic mouse lines, the milk protein-encoding genes like *Wap* and *Csn2* are exclusively expressed in differentiated alveolar cells but not in the AlveoProg cells.** The reason why the small population of AlveoDiff cells could contribute such a global contamination with such a high expression level is that these **milk protein-coding genes are controlled by extremely strong mammary-specific super-enhancers driven by hormones and transcribed at an extremely high level during lactation**. In addition, the mRNAs of these specific genes are also more stable than other genes in lactation, resulting in the phenomenon that the mRNAs from around 10 milk protein-encoding genes occupy 90% of total mRNAs of all genes in the lactating mammary gland. The extreme abundance of these genes’ RNAs in the tissue leads to the dominant contamination. These works have been published in *Nat Genet* and *Exp Mol Med* [14, 15].

Reviewer

7. I appreciate that the authors tested the method on 12 datasets and listed all cell-type-specific GCGs assuming the proposed method works properly. However, I am not convinced about the conclusion that contamination is more severe in snRNAseq data than in scRNA-seq data based on Wasserstein distances across different tissues. The tissue heterogeneity should be considered. Even for the same tissue, mammary glands before and after lactating, may be different.

Response

We agree that the tissue heterogeneity should be considered. To strengthen our conclusion, now we compare the snRNA-seq data generated in this study and published scRNA-seq dataset in lactating mammary gland, and we show that the ratio of GCG expression in eGCG- vs. eGCG+ cells is higher in snRNA-seq than that in scRNA-seq data (Supplementary Figure 10B, see below), suggesting the amount of ambient RNAs is closer to that of endogenous RNAs in snRNA-seq data. This result supports our hypothesis.

Minor Comments:

Reviewer

8. Benchmarking codes should be publicly available.

Response

All the codes in this manuscript have been uploaded to Zenodo and will be publicly available if the manuscript is accepted.

Reviewer

9. Some typos should be fixed.

Response

We are sorry for the typos. Now we have corrected all the typos we have found.

Reviewer

10. Figure 4b, the Y-axis is the average entropy divergence across all cell types. This needed to be clarified.

Response

This is clarified in the legends now.

**References**

1.	Hennighausen LG, Sippel AE: Characterization and cloning of the mRNAs specific for the lactating mouse mammary gland. Eur J Biochem 1982, 125:131-141.

2.	Robinson GW, McKnight RA, Smith GH, Hennighausen L: Mammary epithelial cells undergo secretory differentiation in cycling virgins but require pregnancy for the establishment of terminal differentiation. Development 1995, 121:2079-2090.

3.	Deltour L, Leduque P, Blume N, Madsen O, Dubois P, Jami J, Bucchini D: Differential expression of the two nonallelic proinsulin genes in the developing mouse embryo. Proc Natl Acad Sci U S A 1993, 90:527-531.

4.	Petri A, Ahnfelt-Ronne J, Frederiksen KS, Edwards DG, Madsen D, Serup P, Fleckner J, Heller RS: The effect of neurogenin3 deficiency on pancreatic gene expression in embryonic mice. J Mol Endocrinol 2006, 37:301-316.

5.	Rios AC, Fu NY, Jamieson PR, Pal B, Whitehead L, Nicholas KR, Lindeman GJ, Visvader JE: Essential role for a novel population of binucleated mammary epithelial cells in lactation. Nat Commun 2016, 7:11400.

6.	Lemay DG, Ballard OA, Hughes MA, Morrow AL, Horseman ND, Nommsen-Rivers LA: RNA sequencing of the human milk fat layer transcriptome reveals distinct gene expression profiles at three stages of lactation. PLoS One 2013, 8:e67531.

7.	Janssen P, Kliesmete Z, Vieth B, Adiconis X, Simmons S, Marshall J, McCabe C, Heyn H, Levin JZ, Enard W, Hellmann I: The effect of background noise and its removal on the analysis of single-cell expression data. Genome Biol 2023, 24:140.

8.	Hao Y, Hao S, Andersen-Nissen E, Mauck WM, 3rd, Zheng S, Butler A, Lee MJ, Wilk AJ, Darby C, Zager M, et al: Integrated analysis of multimodal single-cell data. Cell 2021, 184:3573-3587 e3529.

9.	Aran D, Looney AP, Liu L, Wu E, Fong V, Hsu A, Chak S, Naikawadi RP, Wolters PJ, Abate AR, et al: Reference-based analysis of lung single-cell sequencing reveals a transitional profibrotic macrophage. Nat Immunol 2019, 20:163-172.

10.	Cao ZJ, Wei L, Lu S, Yang DC, Gao G: Searching large-scale scRNA-seq databases via unbiased cell embedding with Cell BLAST. Nat Commun 2020, 11:3458.

11.	Li C, Liu B, Kang B, Liu Z, Liu Y, Chen C, Ren X, Zhang Z: SciBet as a portable and fast single cell type identifier. Nat Commun 2020, 11:1818.

12.	Hu C, Li T, Xu Y, Zhang X, Li F, Bai J, Chen J, Jiang W, Yang K, Ou Q, et al: CellMarker 2.0: an updated database of manually curated cell markers in human/mouse and web tools based on scRNA-seq data. Nucleic Acids Res 2023, 51:D870-D876.

13.	Franzen O, Gan LM, Bjorkegren JLM: PanglaoDB: a web server for exploration of mouse and human single-cell RNA sequencing data. Database (Oxford) 2019, 2019.

14.	Shin HY, Willi M, HyunYoo K, Zeng X, Wang C, Metser G, Hennighausen L: Hierarchy within the mammary STAT5-driven Wap super-enhancer. Nat Genet 2016, 48:904-911.

15.	Zeng X, Lee HK, Wang C, Achikeh P, Liu C, Hennighausen L: The interdependence of mammary-specific super-enhancers and their native promoters facilitates gene activation during pregnancy. Exp Mol Med 2020, 52:682-690.

**Second round of review**

**Reviewer 1**

1. Supp Figure 3 shows gene count data in empty droplets from public data. The exact number of genes is unclear from the figure, although the manuscript notes up to hundreds of genes. Based on the genes labelled, they appear to vary considerably between tissue types. What is the rationale that only genes in empty droplets can be in ambient RNA background? Other global methods are shown to detect other transcripts (not flagged as GCGs). An alternative explanation is that there is bias with some ‘true ambient transcripts more likely to interact with cell surface membranes. This has been shown before in the analysis of cancer single-cell data (Wu et al. Nat Gen 2020).
2. I would recommend considering the narrative (and demonstration) of the benefits of scCDC over other ambient RNA detection methods. In the new comparison of the barnyard experiments (mixed mouse and human cells), scCDC comes off as 3rd best to DecontX and scAR, and just marginally better than other methods. It’s stated that other methods ‘over-correct’, but the results (sup fig 6a, fig 5b) show a slight over-correction of two methods, in one dataset*, and others, which also appear to perform better than scCDC. * it’s stated that this is happening in many genes from mammary gland data (fig 1), but just a few are shown and don’t appear to be compared to scCDC performance.
3. The ‘barnyard’ experiment is the closest to the gold standard for benchmarking – and here, scCDC does not perform best. For the single species-only data (PBMCs, Pancreas, liver, mammary gland), there is no clear way to determine what is ambient vs not. Comparing the performance of scCDC to other methods against GCGs identified from scCDC will lead to a biased interpretation, as true ambient RNA not detected as GCGs will be missed. As mentioned above, using benchmarking in an openly competitive way should help identify instances where scCDC offers an advantage over other tools.
4. Has the code been updated? I retested the barnyard experiment and got the same results as before – only 9 genes were detected as GCGs, and hundreds of ambient genes missing from the GCG list.

**Reviewer 2**

In the revision, authors addressed most of my comments. Generally, the quality of the manuscript has been improved. However, there are still some remaining concerns that need further clarification and evidence below.

1. The logic of considering the pre-clustering is too strong is that, if we first cluster on a contaminated dataset and then correct it, the contamination will affect the pre-clustering. Though the authors have listed some evidence to argue the impact is small, it is interesting to see what would happen if we iteratively run your method on a contaminated dataset? Will the performance be improved, or over-correction would happen? Some discussion is needed.

2. The authors emphasize the potency of scCDC, which offers several advantages over alternative methods. More discussion on the limitations of the method is needed. For instance, scCDC is effective in reducing redundant expression values, yet it doesn't entirely eliminate the highest values present in other cell types. An in-depth analysis or discussion of the impact of these high-value contaminations would enhance the understanding of the method's performance. Additionally, providing a summary of the performance results for all datasets listed in supplementary table 2 would be highly beneficial.

4. It is interesting to show the mean entropy curve for the developmental dataset such as simulated pancreas datasets. It would be more convincing for me to see how the contaminated genes were identified from this kind of data.

5. I suggest authors further polish this manuscript. Some sentences are not well-connected in reading.

**Authors Response**

**Point-by-point responses to the reviewers’ comments:**

Reviewer #1:

1.	Supp Figure 3 shows gene count data in empty droplets from public data. The exact number of genes is unclear from the figure, although the manuscript notes up to hundreds of genes. Based on the genes labeled, they appear to vary considerably between tissue types. What is the rationale that only genes in empty droplets can be in ambient RNA background? Other global methods are shown to detect other transcripts (not flagged as GCGs). An alternative explanation is that there is bias with some 'true ambient transcripts more likely to interact with cell surface membranes. This has been shown before in the analysis of cancer single-cell data (Wu et al. Nat Gen 2020).

Answer: To clarify, in the Supplementary Figure 3 (see below), we ranked all genes based on their counts in empty droplets and verified that the GCGs detected by scCDC have the largest counts in empty droplets. On top of each panel, we have added the number of GCGs. We did not mean to say that only the genes detected in empty droplets have ambient RNAs. Instead, we deem it reasonable to say that the genes with large counts in empty droplets are more likely to have severe contaminations and are thus GCGs. We note that the RNAs in empty droplets are helpful in estimating the background ambient RNA levels—an assumption used in several existing methods, such as SoupX, scAR and CellBender.

**Supplementary Figure 3.** A small group of genes mainly contributes ambient RNAs in empty droplets. The plots show the counts vs. ranks of genes in empty droplets in the indicated datasets. The dashed lines separate the ‘super-contaminative’ genes and the other genes (details in Method Appendix. See Additional File 1).

2.	I would recommend considering the narrative (and demonstration) of the benefits of scCDC over other ambient RNA detection methods. In the new comparison of the barnyard experiments (mixed mouse and human cells), scCDC comes off as 3rd best to DecontX and scAR and just marginally better than other methods. It's stated that other methods 'over-correct', but the results (sup fig 6a, fig 5b) show a slight over-correction of two methods, in one dataset*, and others, which also appear to perform better than scCDC. * it's stated that this is happening in many genes from mammary gland data (fig 1), but just a few are shown and don't appear to be compared to scCDC performance.

Answer: In response to the reviewer's suggestion of demonstrating the benefits of scCDC, we have applied scCDC and four existing methods (DecontX, CellBender, SoupX, and scAR) to fifteen real datasets. This analysis revealed the under-correction of highly-contaminating GCGs, including cell-type marker genes, by DecontX, SoupX-automated, and CellBender. Additionally, we have observed the over-correction of lowly-/non-contaminating genes, particularly housekeeping genes, by SoupX-manual and scAR. The scCDC addressed these limitations, providing robust correction on highly-contaminating GCGs and avoiding over-correction on the other genes (Figure 3F, Figure 4 and Supplementary Table 3, the figures are included on page 5-6 of this letter). We also disagree with some of the reviewer’s conclusions. Supplementary Figure 8A (see page 7 of this letter) shows that scAR clearly exhibited over-correction in the “barnyard” dataset. Moreover, in Supplementary Figure 7C, Figure1E and Supplementary Figure 2 (see page 7-8 of this letter), we demonstrate the severe, not slight, over-correction of many housekeeping genes by SoupX and scAR in the PBMC and mammary gland datasets. The over-correction by SoupX and scAR was also observed in other datasets in our analysis (Supplementary Table 3). In comparison, scCDC, DecontX, and CellBender did not show over-correction of these genes.

**Figure 3.** Contamination detection by scCDC in snRNA-seq and scRNA-seq datasets. Notably, the ‘barnyard’ dataset shows overall low contamination level compared to other datasets in real tissues. (E) scCDC identifies highly-contaminating genes in the ‘barnyard’ scRNA-seq dataset of mixed human 293T cells and mouse 3T3 cells. The scatter plots show the average counts vs. rank of cross-species contaminating genes in the indicated cells. The average cross-species contaminative counts of GCG and non-GCGs are shown in the boxplot on the right. (F) Contamination ratios of GCGs vary widely in different datasets. Box plots show the contamination ratios of GCGs in the indicated datasets.

**Figure 4.** Correction on GCGs by scCDC, DecontX, SoupX, CellBender, and scAR in real datasets. scCDC is the most robust method of correcting highly-contaminating GCGs. (A) The box plots show the medium expression of GCGs in eGCG- cells before and after correction in the indicated datasets. (B) DecontX outperforms in the single-cell RNA-seq dataset of mixed human 293T cells and mouse 3T3 cells; while scCDC performance is secondary to DecontX’s. Box plots show the counts of GCGs before and after correction by the indicated method in the ‘barnyard’ scRNA-seq dataset of mixed human 293T cells and mouse 3T3 cells. Of note, SoupX-automated fails to run on the data. *, no output. (C) scCDC efficiently corrects highly-contaminating genes in the snRNA-seq dataset in the lactating mammary gland. The violin plots show the normalized expression levels of the indicated GCGs before and after correction using scCDC. (D) DecontX and SoupX-automated displayed under-correction in the scRNA-seq data in mouse pancreas islet with spike-in strategies. The violin plots show the normalized expression levels of the indicated GCGs before and after correction using the indicated methods by the default Seurat (V3).

**Supplementary Figure 8A.** Over-correction of non-contaminating genes by scAR in the ‘barnyard’ scRNA-seq data of mixed human 293T and mouse 3T3 cells. (A) The box plots show the average counts of endogenous genes in the indicated cells. *, no output.

**Supplementary Figure 7C.** Over-correction by SoupX and scAR in the PBMC scRNA-seq dataset. Q-Q plots of the 95^th^ percentile value of counts of 66 housekeeping genes in all the cells after correction by the indicated methods compared with the original value of counts.

**Figure 1E.** Over-correction of housekeeping genes by SoupX-manual and scAR in our in-house contaminative mammary gland snRNA-seq datasets. The violin plots show the normalized expression levels of the selected housekeeping genes before and after correction using the indicated methods.

**Supplementary Figure 2.** Overcorrection by SoupX and scAR in snRNA-seq mammary gland datasets. Q-Q plots of the 95^th^ percentile value of counts of 66 housekeeping genes in all the cells after correction by the indicated methods compared with the original value of counts in virgin (right) and lactating (left) mammary gland snRNA-seq datasets.

3.	The 'barnyard' experiment is the closest to the gold standard for benchmarking - and here, scCDC does not perform best. For the single species-only data (PBMCs, Pancreas, liver, mammary gland), there is no clear way to determine what is ambient vs not. Comparing the performance of scCDC to other methods against GCGs identified from scCDC will lead to a biased interpretation, as true ambient RNA not detected as GCGs will be missed. As mentioned above, using benchmarking in an openly competitive way should help identify instances where scCDC offers an advantage over other tools.

Answer: We argue that the “barnyard” dataset only represents a slight contamination case (as we show in Figure 3E-F, see page 5 of this letter), on which most existing methods have good performance in removing the contamination (Figure 4, see page 6 of this letter). However, when applied to datasets with highly-contaminating genes, we observed the limitations of current methods. Using the real datasets from different tissues, we show the under-correction or over-correction performance of the existing methods based on biological knowledge (Figure 1D-E, Supplementary Figure 2,7 and 10, see page 9-13 of this letter). Acknowledging that these real datasets should not be regarded as ground truths, we used simulated datasets to confirm that DecontX was good at correcting lowly-contaminating genes but failed to provide sufficient correction on highly-contaminating ones. In contrast, scCDC exhibited robust correction on highly-contaminating genes but may omit some lowly-contaminating ones. Furthermore, we show that the combined use of DecontX and scCDC would leverage the complementary advantages of the two methods. The results are shown in Figure 5 (see page 14 of this letter).

**Figure 1.** Performance evaluation of existing methods on correcting contaminated mammary gland snRNA-seq data. DecontX, SoupX-automated and CellBender over-corrects contaminating cell-type marker genes, while SoupX-manual and scAR over-corrects housekeeping genes. (D-E) The violin plots show the normalized expression levels of the selected marker genes (D) and housekeeping genes (E) before and after correction using the indicated methods by the default Seurat (V3). Adipo, adipocytes; AlveoProg, alveolar progenitors; AlveoDiff, differentiated alveolar cells; Bas/Myo, basal cells/myoepithelial cells; Endo, endothelial cells; Fibro, fibroblasts; HormSens, hormone sensing cells; HormSensDiff, differentiated hormone sensing cells; HormSensProg, hormone sensing progenitors; Immune, immune cells; LumProg, luminal progenitors; SkelMusc, skeleton muscle cells.

**Supplementary Figure 7.** Comparison of the performance of scCDC, DecontX, SoupX, CellBender, and scAR on the PBMC scRNA-seq dataset. DecontX, SoupX-automated and CellBender over-corrects contaminating the cell-type marker gene *CD74*, while SoupX-manual and scAR over-corrects housekeeping genes. (A) GCGs identified by scCDC are genes contributing the most abundant ambient RNAs. The plot shows the counts vs. rank of genes in the empty droplets. GCGs are labeled. The dashed line separates the ‘super-contaminating’ genes and the other genes (details in Method Appendix. See Additional File 1). (B) The violin plots show the normalized expression levels of *LYZ* and *CD74* before and after correction using indicated methods in the PBMC-4K dataset. (C) Over-correction by SoupX and scAR in the PBMC scRNA-seq dataset. Q-Q plots of the 95^th^ percentile value of counts of 66 housekeeping genes in all the cells after correction by the indicated methods compared with original value of counts.

**Supplementary Figure 9.** Comparison of scCDC, DecontX, SoupX, CellBender, and scAR on the scRNA-seq data in the human liver. DecontX, SoupX-automated and CellBender over-corrects contaminating cell-type marker genes, while SoupX-manual and scAR over-corrects housekeeping genes. (A) Global contamination is detected in the dataset. The expression of erythroid marker *HBB* and hepatocyte markers *ALB* and *APOA2* in the cells are shown in the UMAP plots. (B) DecontX and SoupX-manual under-correct the contamination. The violin plots show the normalized expression levels of the indicated GCGs before and after correction using the indicated methods by the default Seurat (V3).(C) scAR moderately over-correct the data. Q-Q plots of the 95th percentile value of counts of 66 housekeeping genes in all the cells after correction by the indicated methods compared with original value of counts.

**Supplementary Figure 10.** Comparison of scCDC, DecontX, SoupX, CellBender, and scAR on the scRNA-seq data in mouse pancreas islet with spike-in strategy. DecontX, SoupX-automated and CellBender over-corrects contaminating cell-type marker genes, while SoupX-manual and scAR over-corrects housekeeping genes. ­(A) DecontX and SoupX-manual under-correct contamination caused by GCGs. The barplots show the percentage of cells expressing GCGs in the cell clusters before and after correction using the indicated methods. (B) The correction results by scCDC, SoupX-manual and scAR highly correlates with the one corrected using spike-in strategy. The heatmap shows *Spearman*’s correlation between the mean expression levels of GCGs in the cell clusters in pancreas data after correction by different computational methods and by spike-in approach. (C-D) Over-correction by SoupX and scAR in pancreas scRNA-seq dataset. (C) The violin plots show the normalized expression levels of the indicated GCGs before and after correction using the indicated methods by the default Seurat (V3). (D) Density plots of the 95^th^ percentile value of counts of 66 housekeeping genes in all the cells before and after correction by the indicated methods in the pancreas scRNA-seq datasets.

**Figure 5.** Simulation confirmed the effectiveness of scCDC in correcting highly-contaminating genes and suggested combined use of scCDC and DecontX. (A) The diagram shows the simulation strategy of artificial contamination with different levels. Artificial contamination of 500 genes at low, medium and high levels is added to the uncontaminated simulated data. (B) Scatter plots show the artificially added contaminative counts vs. corrected counts of the 500 genes in (A). (C) Scatter plots show the counts before and after correction of the genes that are not artificially contaminated. (D) The violin plots show the normalized expression levels of indicated top GCGs before and after correction by different computational methods.

4.	Has the code been updated? I retested the barnyard experiment and got the same results as before - only 9 genes were detected as GCGs, and hundreds of ambient genes missing from the GCG list.

Answer: We double-checked our code and confirmed that 290 GCGs were identified by scCDC in the barnyard experiment dataset. Some examples are shown in the figure below.

**Unpublished data:** GCGs are identified by scCDC in the ‘barnyard’ dataset. The UMAP plots show the expression levels of 20 representative GCGs in the cells.

Reviewer #2: In the revision, authors addressed most of my comments. Generally, the quality of the manuscript has been improved. However, there are still some remaining concerns that need further clarification and evidence below.

1. The logic of considering the pre-clustering is too strong is that, if we first cluster on a contaminated dataset and then correct it, the contamination will affect the pre-clustering. Though the authors have listed some evidence to argue the impact is small, it is interesting to see what would happen if we iteratively run your method on a contaminated dataset? Will the performance be improved, or over-correction would happen? Some discussion is needed.

Answer:

We thank the reviewer for the comment. However, we would like to note that pre-clustering is a necessary step in most single-cell data analysis tasks. Although the methods like DecontX and SoupX do not require a mandatory pre-clustering step, we found that using supervised pre-clustering significantly improved the performance of DecontX and SoupX in our results (Figures 1D, 4A, 4B and 4D, see page 17-18 of this letter). Note that we also suggested an approach to help determine the resolution parameter used in pre-clustering (method appendix in Additional File 1).

As suggested, we also use the simulated data to investigate if an iterative application of scCDC (i.e., applying scCDC again to its corrected data from the previous iteration) could improve the decontamination accuracy,. Our results from three iterations showed that the number of GCGs dramatically decreased after the first iteration and became stable (Supplementary Figure 12A, see page 19 of this letter). Of note, few genes were identified as GCGs in more than one iteration, and their corrected expression remained stable after the first iteration (Supplementary Figure 12B). Meanwhile, housekeeping genes were not found as GCGs in all iterations, as expected (Supplementary Figure 12C). Similar results were observed when scCDC was iteratively applied to our in-house dataset of lactating mammary glands (Supplementary Table 4). These results suggest that a one iteration of scCDC (GCG detection followed by correction) is sufficient.

**Figure 1D** The violin plots show the normalized expression levels of the selected marker genes before and after correction using the indicated methods by the default Seurat (V3) in the snRNA-seq datasets in mammary glands.

**Figure 4.** Correction of GCGs by scCDC, DecontX, SoupX, CellBender, and scAR in real datasets. scCDC is the most robust method correcting highly-contaminating genes. (A) The box plots show the medium expression of GCGs in eGCG- cells before and after correction in the indicated datasets. (B) DecontX outperforms in the single-cell RNA-seq dataset of mixed human 293T cells and mouse 3T3 cells; while scCDC performance is secondary to DecontX’s. Box plots show the counts of GCGs before and after correction by the indicated method in the ‘barnyard’ scRNA-seq dataset of mixed human 293T cells and mouse 3T3 cells. Of note, SoupX-automated fails to run on the data. *, no output. (C) scCDC efficiently corrects highly-contaminating genes in the snRNA-seq dataset in the lactating mammary gland. The violin plots show the normalized expression levels of the indicated GCGs before and after correction using scCDC. (D) DecontX and SoupX-automated displayed under-correction in the scRNA-seq data in mouse pancreas islets with spike-in strategies. The violin plots show the normalized expression levels of the indicated GCGs before and after correction using the indicated methods by the default Seurat (V3).

**Supplementary Figure 12.** One-time application of scCDC is sufficient for contamination correction in the simulated scRNA-seq dataset. The simulated scRNA-seq dataset with medium artificial contamination in Figure 6 was treated by scCDC iteratively. (A) The majority of GCGs are identified in the first run when scCDC is iteratively applied to the simulated pancreas dataset. The table presents the GCGs identified by scCDC in three iterative runs. (B-C) The iterative application of scCDC does not lead to over-correction. (B) No obvious correction is detected in GCGs. Scatter plots show the artificial contaminative counts vs. corrected counts of the 500 artificial contaminative genes in three iterative rounds of scCDC correction. (C) No obvious correction is detected in uncontaminated genes. Q-Q plots of the 95th percentile value of counts of 66 housekeeping genes in all the cells after each round of correction compared to the original value of counts.

2. The authors emphasize the potency of scCDC, which offers several advantages over alternative methods. More discussion on the limitations of the method is needed. For instance, scCDC is effective in reducing redundant expression values, yet it doesn't entirely eliminate the highest values present in other cell types. An in-depth analysis or discussion of the impact of these high-value contaminations would enhance the understanding of the method's performance. Additionally, providing a summary of the performance results for all datasets listed in supplementary table 2 would be highly beneficial.

Answer:

We thank the reviewer for the suggestion. As suggested, we discussed the limitations of our method in the revised manuscript now. We pointed out that scCDC may not be able to identify some lowly-contaminating genes as GCGs and does not provide correction for them. To overcome this limitation, scCDC may be applied in combination with other methods, such as DecontX. We also discussed the possible highest values left after correction. In correcting the counts on GCGs, scCDC, SoupX-manual, and scAR are all effective methods, correcting the median expression of GCGs in eGCG- cells to around zero in most datasets (Figure 4A, see page 18 of this letter). Nevertheless, none of the methods could clear all the counts of GCGs in eGCG- cells, leaving certain contaminative counts of GCGs in a small population of eGCG- cells (Supplementary Table 2), which may slightly affect cell clustering and other analyses. Of note, we were able to design scCDC to clear the counts of GCGs in eGCG- cells aggressively. However, this strategy will alter the natural count distribution of GCGs in the entire dataset and may hinder the combined use of scCDC with other methods. We have included these in the discussion now. Regarding the suggestion of summarizing the performance results, we now provide the median value and proportion of clearance of GCGs in eGCG- clusters in all the datasets listed in Supplementary Table 2 (Supplementary Table 1 after manuscript reorganization). The data are included in the Supplementary Table 4. In addition, in the Supplementary Table 3 we summarized the observed under-correction of GCGs and the over-correction of housekeeping genes and lowly-contaminating GCGs by each of the five methods in all the datasets we have tested.

Note that Reviewer 2 had no Comment 3.

4. It is interesting to show the mean entropy curve for the developmental dataset, such as simulated pancreas datasets. It would be more convincing for me to see how the contaminated genes were identified from this kind of data.

Answer: We now provide the data showing all the mean entropy curves and the process of GCG identification in the dataset in Supplementary Figure 13 and Figure 6B.

**Supplementary Figure 13.** scCDC identifies GCGs in simulated scRNA-seq trajectory dataset with contamination. (A) The UMAP shows cell clustering result in the simulated dataset by the default Seurat (V3). (B) The entropy-expression curves of indicated clusters. Identified candidate GCGs in each cluster are highlighted in orange, and GCGs are highlighted in red and labeled on the plots.

**Figure 6B.** scCDC successfully identifies artificial contaminative genes. Upset plot shows the identification of GCGs from candidate GCGs from each cluster. GCGs are highlighted in red, and the artificial contaminative genes are labeled.

5. I suggest authors further polish this manuscript. Some sentences are not well-connected in reading.

Answer: We thank the reviewer for the suggestion. We have made substantial revisions to our manuscript. As a result, we have reorganized the content and polished the sentences to enhance the overall flow and readability of the document.

**Third round of Review**

**Reviewer 2**

Authors have done a good job addresing my remaining comments.
